# Atomic Insights into the Competitive Edge of Nanosheets Splitting Water

**DOI:** 10.1021/jacs.4c10312

**Published:** 2024-09-25

**Authors:** Lorenz J. Falling, Woosun Jang, Sourav Laha, Thomas Götsch, Maxwell W. Terban, Sebastian Bette, Rik Mom, Juan-Jesús Velasco-Vélez, Frank Girgsdies, Detre Teschner, Andrey Tarasov, Cheng-Hao Chuang, Thomas Lunkenbein, Axel Knop-Gericke, Daniel Weber, Robert Dinnebier, Bettina V. Lotsch, Robert Schlögl, Travis E. Jones

**Affiliations:** † Fritz Haber Institute of the Max Planck Society, Berlin 14195, Germany; ‡ School of Natural Sciences, Technical University, Munich 85748, Germany; § Integrated Science & Engineering Division, Underwood International College, Yonsei University, Incheon 21983, Republic of Korea; ∥ Department of Chemistry, National Institute of Technology Durgapur, Durgapur 713209, West Bengal, India; ⊥ Max Planck Institute for Solid State Research, Stuttgart 70569, Germany; # Leiden Institute of Chemistry, Leiden University, 2300 Leiden, RA, Netherlands; 7 Experiments Division, ALBA Synchrotron Light Source, Cerdanyola del Vallés, Barcelona 08290, Spain; 8 Department of Physics, Tamkang University, New Taipei City 251301, Taiwan; 9 Wallenberg Initiative Materials Science for Sustainability, Chemistry and Chemical Engineering, Chalmers University of Technology, Gothenburg 41296, Sweden; 10 Theoretical Division, Los Alamos National Laboratory, Los Alamos, New Mexico 87545, United States

## Abstract

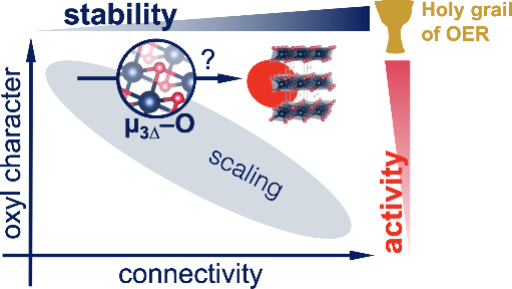

The oxygen evolution reaction (OER) provides the protons for many electrocatalytic power-to-X processes, such as the production of green hydrogen from water or methanol from CO_2_. Iridium oxohydroxides (IOHs) are outstanding catalysts for this reaction because they strike a unique balance between activity and stability in acidic electrolytes. Within IOHs, this balance varies with the atomic structure. While amorphous IOHs perform best, they are least stable. The opposite is true for their crystalline counterparts. These rules-of-thumb are used to reduce the loading of scarce IOH catalysts and retain the performance. However, it is not fully understood how activity and stability are related at the atomic level, hampering rational design. Herein, we provide simple design rules (Figure 12) derived from the literature and various IOHs within this study. We chose crystalline IrOOH nanosheets as our lead material because they provide excellent catalyst utilization and a predictable structure. We found that IrOOH signals the chemical stability of crystalline IOHs while surpassing the activity of amorphous IOHs. Their dense bonding network of pyramidal trivalent oxygens (μ_3Δ_-O) provides structural integrity, while allowing reversible reduction to an electronically gapped state that diminishes the destructive effect of reductive potentials. The reactivity originates from coordinative unsaturated edge sites with radical character, i.e., μ_1_-O oxyls. By comparing to other IOHs and literature, we generalized our findings and synthesized a set of simple rules that allow prediction of stability and reactivity of IOHs from atomistic models. We hope that these rules will inspire atomic design strategies for future OER catalysts.

## Introduction

Proton exchange membrane (PEM) electrolyzers can produce green hydrogen dynamically at high purity and pressure.^1−3^ These benefits make them increasingly popular to produce green hydrogen on demand, but PEM electrolyzers cannot fuel a hydrogen economy of scale because of scarce catalyst materials for the oxygen evolution reaction (OER)—one half-reaction of water electrolysis. The state-of-the-art OER catalyst with the best balance of stability and activity are iridium oxohydroxides (IOHs), which make them an interesting model for the development of more abundant catalyst materials, or—at high metal utilization^4^—promising candidates for devices with high-end performance.

Among IOHs, amorphous IOHs are particularly active.^5−8^ In contrast to their crystalline counterparts, amorphous IOHs are hydrated^9−12^ and exhibit large surface areas,^5,13,14^ leading to a lower Ir–O connectivity and lower oxidation states in ambient conditions—typically between Ir^III^ and Ir^IV^.^6,7,15^ Interestingly, the higher activity of amorphous IOHs cannot be fully explained by their larger surface area. Instead, amorphous IOHs are intrinsically more active than their crystalline counterparts.^5,6,13,14,16^ On the downside, they are more prone to transient dissolution during oxidation and reduction.^17−20^ This suggests that lower Ir–O connectivity causes higher intrinsic activities at the cost of stability for noble metal catalysts. However, the correlation is not straightforward. For IOH films calcined between 100 and 600 °C, Geiger et al. found an optimum balance of activity and stability between 400 and 500 °C.^18^ Their findings show that stability and activity are correlated but in a complex way.

Pinpointing which atomic connectivity in amorphous IOHs leads to an optimum balance of activity and stability is not trivial. The problem can be separated into two parts. First, the role of Ir–O species under operating conditions needs to be known, and second, connected to the distribution of Ir–O species, i.e., the connectivity.

The first part has been addressed, among other techniques,^21,22^ by X-ray spectroscopy. X-ray spectroscopy is a tool well suited to study the role of Ir–O species on IOH surfaces, due to their surface sensitivity when using soft X-rays and distinct signals from different Ir–O species.^20,23−26^ In an effort to utilize this spectroscopy under wet conditions, a variety of in situ approaches have been developed^27,28^ and used on iridium dioxide,^29,30^ anodized metallic iridium thin films^30,31^ and nanoparticles,^25,32,33^ amorphous IOHs with varying pretreatment,^20^ and mesoporous IOH films.^34^ By comparing to calculated spectroscopy of a rutile-type IrO_2_ model system, these studies were able to identify electron-deficient oxygen species that are reactive.^25,30−32^ This negative charge transfer behavior between Ir and O occurs when iridium is oxidized beyond Ir^IV^.^32,33,35^ The dynamic behavior of the electron-deficient oxygen species under applied bias was further used to connect Ir–O species to electrochemical oxidation events^20,25^ and their impact on the reaction barrier of the rate-determining step in the OER employing ab initio molecular dynamics.^36^

The preliminary consensus on Ir–O speciation in the above studies is that surface oxygens bound to one iridium atom (μ_1_-O) are oxyls when stripped of all protons and are the most active species, oxygens bound to two iridium atom (μ_2_-O) contribute to a larger surface electron hole density and serve as proton acceptors in the rate-determining O–O coupling step, and the remaining μ_3_-O species contribute to stability through connectivity.

In this work, we use this knowledge about Ir–O species and their properties and expand it to the distribution of μ_*x*_-O species, i.e., the Ir–O connectivity. To that end, we use crystalline IrOOH nanosheets that have a predictable connectivity, such as rutile-type IrO_2_, and have a hydrated structure, such as amorphous IOHs.^37−39^ This hybrid behavior makes IrOOH a suitable model system to explain the relationships among connectivity, activity, and stability for crystalline and amorphous IOHs alike.

Apart from being an interesting model system, IrOOH nanosheets are promising for real world application. They have been reported to be more active than amorphous IOHs,^37−39^ relatively stable,^37,39^ and utilize iridium more effectively than nanoparticles.^37^ These properties combined make IrOOH nanosheets an interesting candidate to lower iridium utilization below 0.01 g_Ir_/kW, a critical limit for large-scale application of PEM electrolyzers.^40^

Our work describes all steps necessary to get from the material IrOOH to a set of qualitative rules about the relationship between connectivity, activity, and stability that apply to most IOHs. It starts with a characterization of the crystal structure of bulk and nanosheet IrOOH. Based on the experimental structure, we predict the electrochemical behavior of IrOOH with ab initio methods. We then tested the predictions with operando X-ray spectroscopy. This interplay between the model and experiment allows us to uncover a relationship of connectivity and function. To generalize the findings, we qualitatively compared IrOOH against the operando spectroscopy of other well-studied IOHs. The result of this comparison is synthesized into a “cheat-sheet” on how connectivity relates to the function of IOHs.

## Results

Bulk IrOOH is synthesized from the precursor material K_0.75_Na_0.25_IrO_2_ by exchanging the cations for protons in 1 M HCl. IrOOH are dark crystallites with a tinge of pink. Exfoliation to nanosheets was done using tetrabutyl ammonium hydroxide (TBAOH), ultrasonication, and centrifugation. For more information on synthesis, please visit the experimental section and Supporting Information (SI).

### IrOOH Crystal Structure

**Figure 1:**
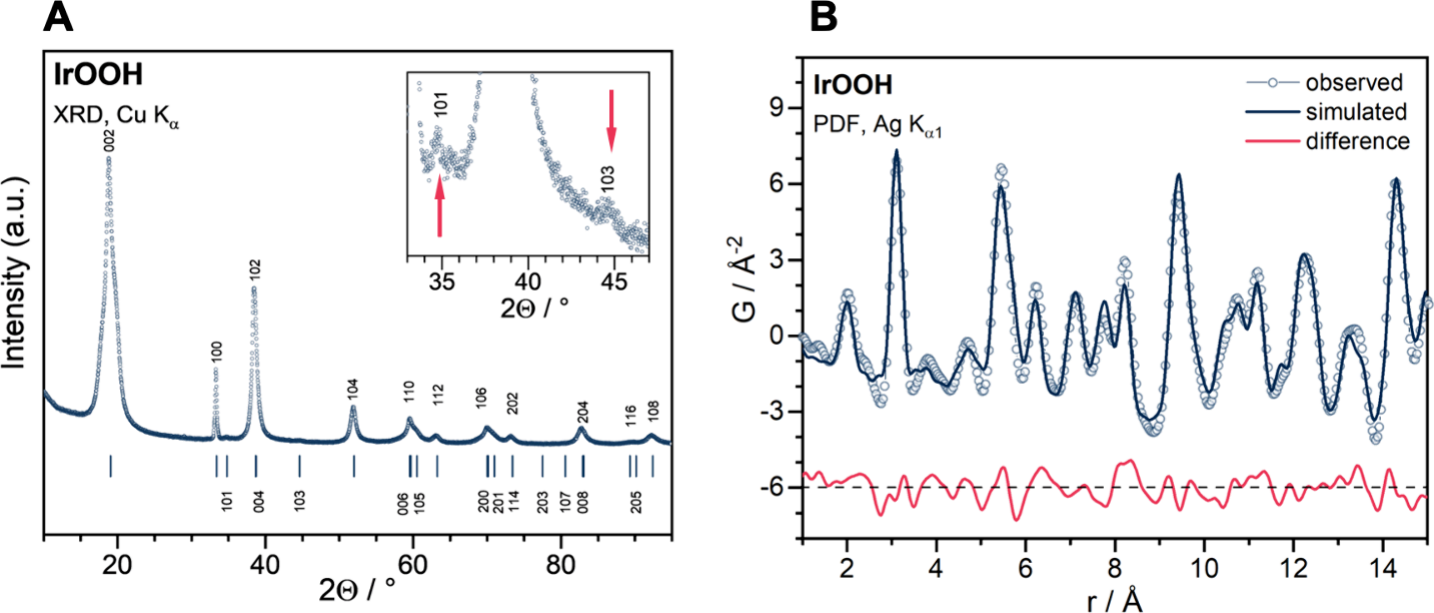
Structural characterization of IrOOH: (A) Diffractogram (blue symbols) of the heterogenite-2*H* with an AB-stacking, Laue indices of intense reflections are given above the curve; the inset shows the two superstructure reflections at 35° and 45° (marked with red arrows); (B) the simulated (dark blue line) and observed (light blue line and symbols) pair distribution function obtained from a heterogenite-*2H* model and IrOOH X-ray scattering, respectively; the difference is shown in red with an offset of −6.

The powder X-ray diffraction (PXRD) pattern of the synthesized IrOOH shown in Figure 1A exhibits sharp *hk*0 reflections demonstrating well-ordered heterogenite type sheets and significant broadening of the 00*l* series indicating domains with varying interlayer spacing, which we will address later in the text. In addition to the brucite-1*T* type pattern, we observe superstructure reflections at ∼35° and ∼44° 2θ (inset of Figure 1A), which can be indexed successfully by AB stacking, i.e., a heterogenite-2*H* type model (space group 194, *P*6_3_/*mmc*). ABC stacking could be ruled out (see SI). Further support for the heterogenite-2*H* structure comes from density functional theory (DFT) calculations, which predicts the formation energy per atom in heterogenite-2*H* to be 0.26 eV lower than that in brucite IrOOH (see Table S2).

The heterogenite-2*H* structure from PXRD was used as a starting point to analyze the pair distribution function (PDF) of IrOOH (Figure 1B). The observed and simulated oscillations of the PDF match well in the local structure range up to 15 Å, indicating good short- to medium-range order (see the SI for more detail). The average Ir–O distance was found to be 2.01 Å, and the closest Ir–Ir distance was 3.11 Å. These distances are close to what was observed by PXRD, namely, 2.05 and 3.10 Å, respectively. DFT calculations of fully relaxed heterogenite-2*H* structures found 2.10 Å for the average Ir–O bond length and 3.23 Å for the closest Ir–Ir distance.

**Figure 2:**
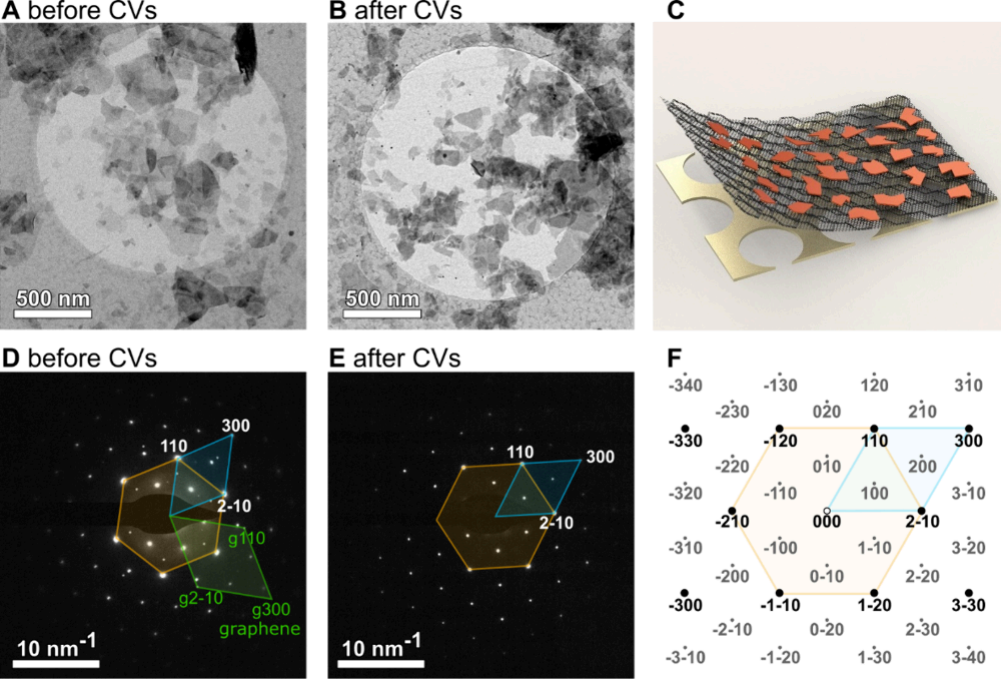
Transmission electron microscope (TEM) bright field micrograph (A) before and (B) after 50 CVs between 0.35 and 1.6 V_RHE_; the respective electron diffractograms of a single sheet are shown in (D,E), in which polygons represent the conventional (orange) and primitive (blue) reciprocal unit cells of IrOOH nanosheets or graphene (green); (F) calculated map of reflection spots for the diffractograms; (C) artistic rendering of the TEM samples with a single layer of graphene (dark gray), nanosheets (red) on a holey TEM gold grid (beige).

The crystal structure of IrOOH nanosheets on a graphene substrate (see experimental details for transfer method) has been characterized on a gold grid (see Figure 2) with selected area electron diffraction (SAED) in a transmission electron microscope (TEM). The crystallites are partly stacked, but single sheets can still be seen clearly (Figure 2A). Combining atomic force microscopy (AFM), TEM, and scanning electron microscopy (SEM) were used to quantify their distribution. We found for thin sheets that about two-thirds of the coverage are single sheets, less than a quarter are double layers, and the rest are three or more layers (Figures S45–S48). The coverage of larger aggregates is about 10% (Figure S46). A single layer is 1.1 ± 0.1 nm thick (Figure S47). Figure 2D shows the SAED of graphene and one IrOOH nanosheet. The latter agrees with a calculated pattern of heterogenite-2*H* (Figure 2F), which resulted in an Ir–Ir distance of 3.15 Å (≈3.11 Å from PXRD). 50 cyclovoltagrams (CVs) between 0.35 and 1.65 V_RHE_ do not alter the structure significantly (see Figure 2B,E and Table S7).

### Electronic Structure and Hydrogen Defects

**Figure 3:**
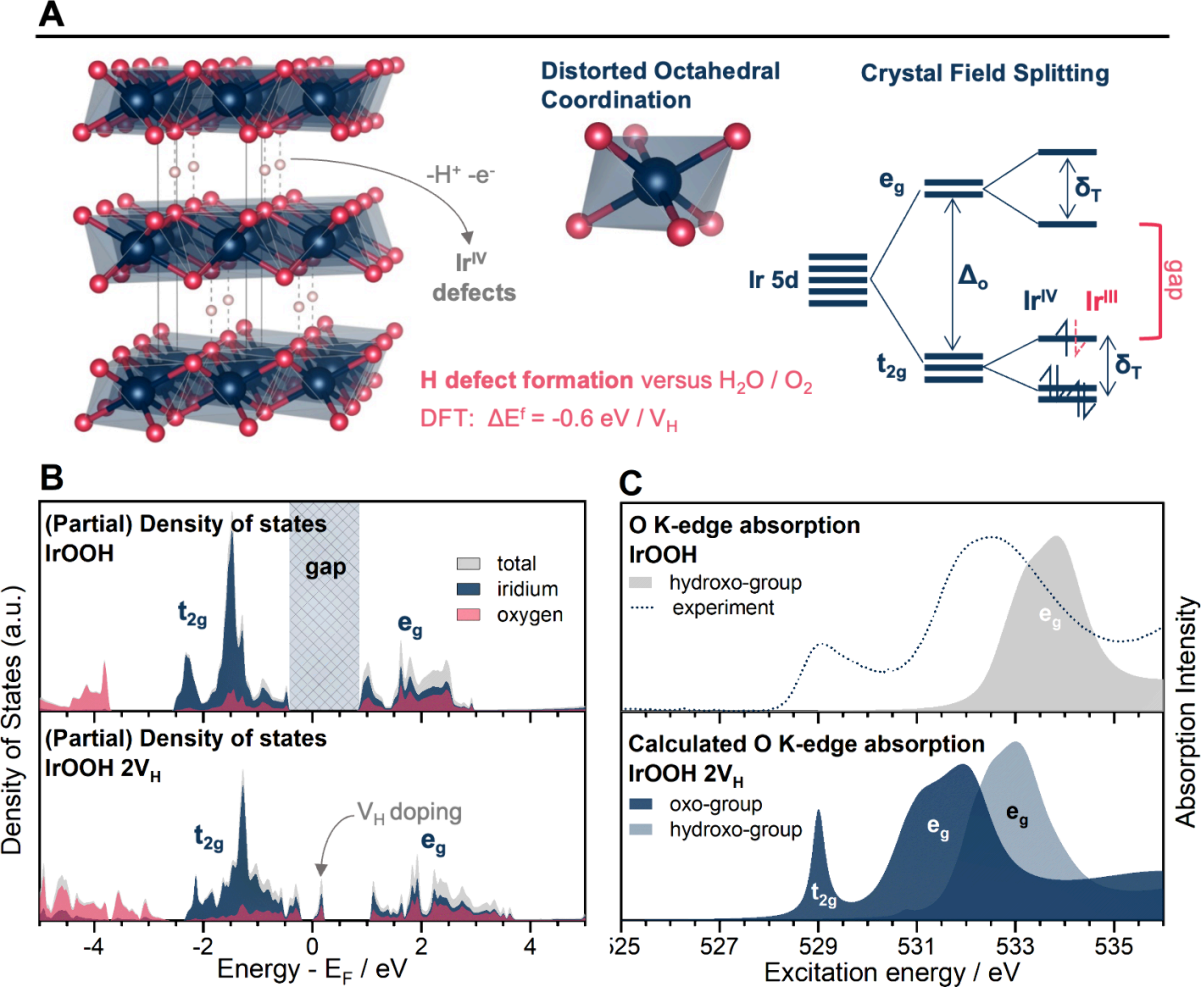
(A) Representation of the heterogenite-2*H* crystal structure and a qualitative scheme of the expected crystal field splitting for the valence states of iridium; dark blue, red, and light pink spheres denote Ir, O, and H atoms, respectively; the crystal field splitting is caused by octahedral coordination with O, which splits the Ir 5d states into states with t_2g_ and e_g_ symmetry, tetragonal distortion further splits these states, the e_g_ being affected more strongly; (B) total and partial density of states (PDOS) relative to the Fermi energy of heterogenite-2*H* IrOOH and a version thereof with one hydrogen vacancy in each layer (2 V_H_), resulting in the sum formula IrO_1.25_OH_0.75_; (C) the respective O K-edge spectra calculated by DFT coupled with the solution of the Bethe–Salpeter equation; the experimental O K-edge absorption of bulk IrOOH is shown as a dotted line.

The atomic positions of iridium and oxygen have a direct impact on the electronic structure of IrOOH and, thereby, their electrochemical function. Iridium in the heterogenite-2*H* structure is octahedrally coordinated by oxygen. The crystal field splits Ir 5d states into states with t_2g_ and e_g_ symmetry, opening a gap between them (see Figure 3A). Tetrahedral distortion introduces further t_2g_ degeneracy, but DFT calculations predict that these distortions do not close the band gap (Figure 3B). The width of the calculated band gap is 1.3 eV, which is in fair agreement with the 1.9 eV from analysis of diffuse UV–vis reflectance (Figure S18), as our DFT method underestimates the magnitude of the gap.^41^ IrOOH is therefore expected to be a semiconductor.

Another tool to probe the crystal field splitting is X-ray absorption spectroscopy (XAS), and we will use K-edge spectroscopy to do so. At first sight, it does not appear valid to probe the crystal field splitting of the metal center via oxygen, but it is in the case of IrOOH. Oxygen and iridium in IrOOH are strongly hybridized, so that the oxygen partial density of states (PDOS) contains information about the metal center and vice versa (see Figures 2B and S26).

In Ir^III^OOH, iridium has an electron configuration of [Xe]6s^0^5d^6^ (Figure 3A) and t_2g_-like states are fully occupied, leaving only excitations into e_g_-like states. The calculated O K-edge spectrum has one main absorption peak at about 534 eV (Figure 3C). However, the experimental O K-edge (shown in addition to the prediction in Figure 3C) shows a second contribution at lower excitation energies, indicating excitations into the lower-lying t_2g_-like states. For this to be true, the average oxidation state of iridium must be larger than +3 (Figure 3A).

This deviation from the formal oxidation of +3 can be caused by hydrogen defects or Ir^IV^ defects (equivalent description). To test if these defects are thermodynamically feasible, we introduced hydrogen vacancies in V_H_ in the ab initio model (Figure 3A). The first two vacancies indeed lowered the formation energy against a reservoir of water and oxygen stepwise, by −0.64 eV each (Table S3). The hydrogen vacancies act as dopants and create states within the gap (Figure 3B). Excitations into these states from the O 1s core level show up as a white line at 529 eV (see SI for energy calibration), matching the experimental O K-edge spectrum (Figure 3C) and further supporting the hypothesis of hydrogen or Ir^IV^ defects.

To quantify how many hydrogen atoms are missing between the layers, we used XAS and temperature-programmed reduction (TPR). Accounting for the possibly undefined hydrogen content, we introduce the general stoichiometry IrO_2–2x_(OH)_2*x*_, with *x* ranging between 0 and 1. For XAS, the integrated white line intensity (WLI) scales with the empty PDOS of the probed element,^42^ i.e., the hole character, or oxidation state. The Ir L_2,3_-edge WLI indicated an iridium oxidation state of +3.2, or IrO_1.2_(OH)_0.8_ (see Figures S20–S22), and the O K-edge WLI indicated an oxidation state of +3.7 or IrO_1.7_(OH)_0.3_ (discussion of Figure S19). For TPR, the initial weight and the amount of consumed hydrogen was used to determine an oxidation state of +3.5, or IrO_1.5_(OH)_0.5_ (see Figures S13 and S14). In other words, every fifth to two-out-of-three hydrogens are IrOOH is missing. The variation originates from uncertainty in the methods (see SI) and uncontrolled exposure to air. For simplicity, we will refer to IrOOH with missing hydrogen atoms as IrO_1.5_(OH)_0.5_, as obtained by TPR. These defects affect the interlayer spacing, the octahedra volume, and octahedra distortion (see Figure S25).

The large amounts of hydrogen defects will also affect interlayer spacing, due to the different diameters of iridium (*d*(Ir^III^) = 1.36 Å and *d*(Ir^IV^) = 1.25 Å) and the change in interlayer bonding (Figure S25). We therefore performed a Rietveld refinement of the PXRD data by introducing domains with varying interlayer spacing via an artificially high isotropic thermal displacement parameter and the Stephens model.^43^ The refined structure was able to explain the degenerated 002 reflex (Figures S4 and S5) with a dominant domain with an interlayer distance of 9.31 Å and two minor domains (9.92 and 8.90 Å). Since DFT indicates an increase in interlayer spacing with hydrogen vacancies (Ir^IV^) while the expected Ir^IV^ ion diameter is smaller, we cannot give a definite answer which minor domain is dominated by Ir^III^ or Ir^IV^.

Now that the atomic and electronic structures of IrO_1.5_(OH)_0.5_ have been well characterized by experiments and are captured by the ab initio model, we will next evaluate the links between the Ir–O connectivity and electrochemical properties of IrOOH with ab initio methods. To reduce transport limitations and get closer to the ab initio structure, we will focus on IrOOH nanosheets.

### Relationship between Connectivity and Electrochemical Properties

**Figure 4:**
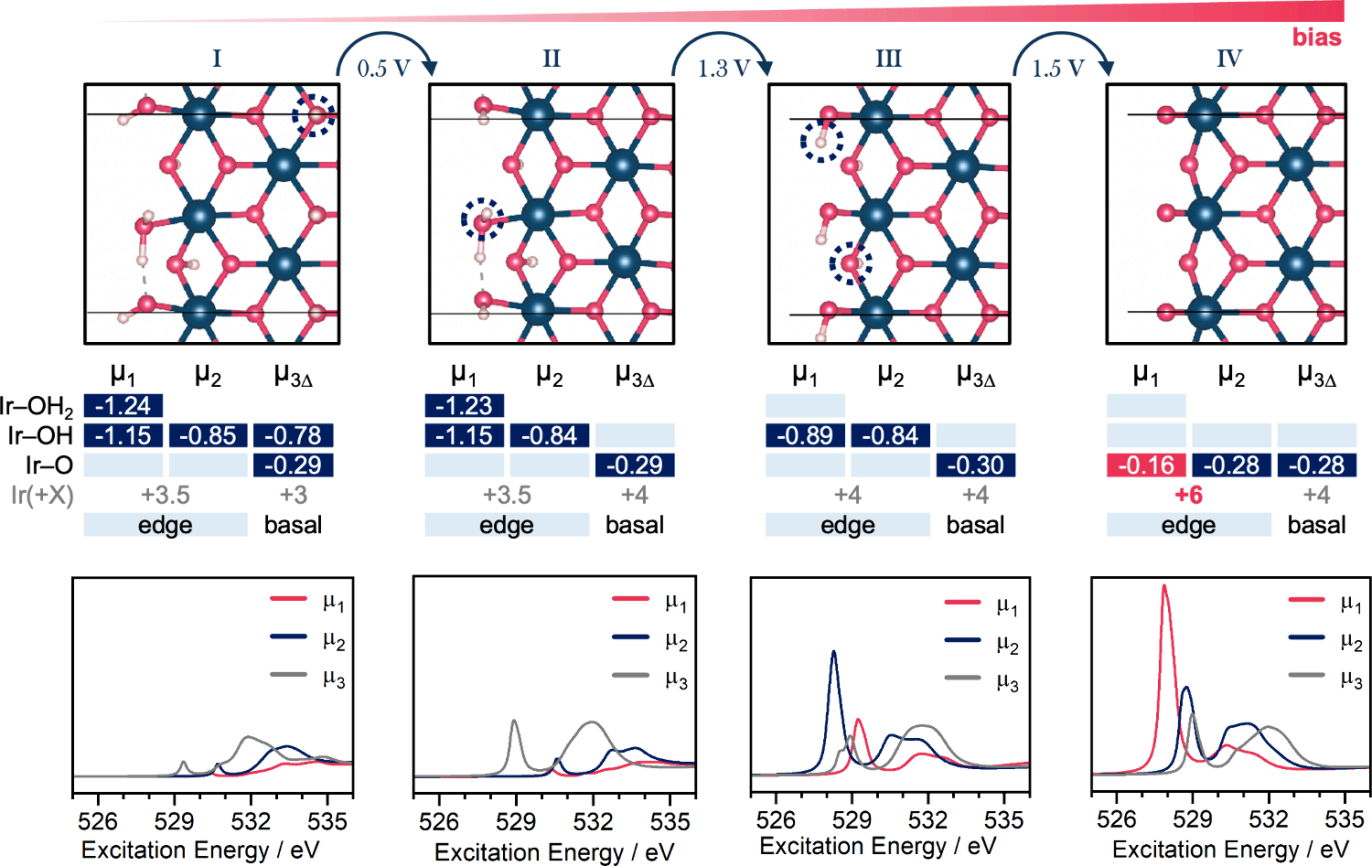
Top shows crystal structure renderings of the most stable IrOOH nanosheet edge structures at a given potential; the potential at which one structure is expected to transition to the other is indicated above them; they are given in Volt against a computational hydrogen electrode; the 3 × 3 tables in the middle row mark the state of hydrogenation of the oxygens bound to one (μ_1_), two (μ_2_), and three (μ_3Δ_) iridium atoms; the values within these dark blue fields indicate the respective charges on oxygen from a Löwdin population analysis with no normalization; the formal oxidation state of iridium in the basal plane and at the edges is given below the tables; the oxidation state +6 is marked in red because it is expected to fall into a negative charge transfer regime in which the holes on iridium are strongly shared with neighboring oxygens; the calculated O K-edges averaged for μ_1_, μ_2_, and μ_3_ are shown on the bottom (see Figure S34 for all spectra); the white line intensity is more intense the more hole character resides on the respective oxygen species.

We start with the horizon of expectation for Ir–O speciation, spectroscopy, and electrochemistry by using ab initio calculations. The DFT calculations (see Experimental Section and SI for computational details) were initiated with the crystal structure from SAED and relaxed within the experimental bounds with respect to the total energy and forces. The resulting Ir–Ir distance of 3.25 Å (see Table S4) is larger than the 3.15 Å from SAED, which is a well-known shortcoming of the generalized gradient approximation we use.^44,45^ The basal plane is entirely made up of what we are going to call trivalent pyramidal oxygen species, or μ_3Δ_-O, which are chemically distinct from μ_3_-O in rutile-type IrO_2_ (see the SI for hydrogen adsorption calculations). The placement of hydrogen on the basal planes was evaluated on continuous sheets. A structure with symmetric and parallel rows of hydrogen had the lowest energy (see SI). The same structure was terminated perpendicular to the (100) direction to arrive at the edge model with a 1:1 ratio between terminal oxygen (μ_1_-O) and bridging oxygen (μ_2_-O) (Figure 4).

With the model at hand, we first evaluate how nanosheet’s protonation changes with bias. Each μ_*x*_-O species exists in a protonated form, i.e., μ_*x*_-OH, and can shed the proton in a proton-coupled electron transfer (PCET), as in μ_*x*_-OH → μ_*x*_-O + e^–^ + H^+^. The nanosheet can be reduced or oxidized using these PCETs. To predict (de)protonation potentials, we compared the thermodynamic stability of 12 edge structures with various amounts of hydrogen against a computational electrode (Figure S32). The following phase transitions occur: Deprotonation of μ_3Δ_-OH at 0.5 V_SHE_, deprotonation of μ_1_-OH_2_ at 1.3 V_SHE_, and deprotonation of μ_2_-OH and μ_1_-OH at 1.5 V_SHE_ (see Figure 4, structures II and III). Assuming an oxidation state of −2 for oxygen and +1 for hydrogen allows determination of the formal oxidation states of iridium, which equates to successive oxidation from +3 to +6 (tabulated in the middle of Figure 4). Beyond +4, IOHs enter a negative charge transfer regime and electron holes increasingly reside on oxygen.^32,33,35^

This hole character on oxygen is known to play a crucial role for the reactivity of IOHs in the OER. We measured this hole character via the Löwdin charges on oxygen (tables within Figure 4) and the O K-edge WLI, which scales with the empty oxygen PDOS of the probed element.^42^ Two trends are observed in the bottom of Figure 4: first, the absorption white line position shifts to lower values with a lower valency, and second, the absorption intensity increases with an increasing 2p hole character on oxygen. When fully deprotonated, the absorption intensity and Löwdin charge of μ_1_-O are distinct from those of the μ_2_-O and μ_3_-O counterparts, which have nearly equal 2p hole character. As shown in earlier studies,^20,25,26,36^ this extensive hole character on μ_1_-O leads to radical character and a high reactivity in O–O coupling.

**Figure 5:**
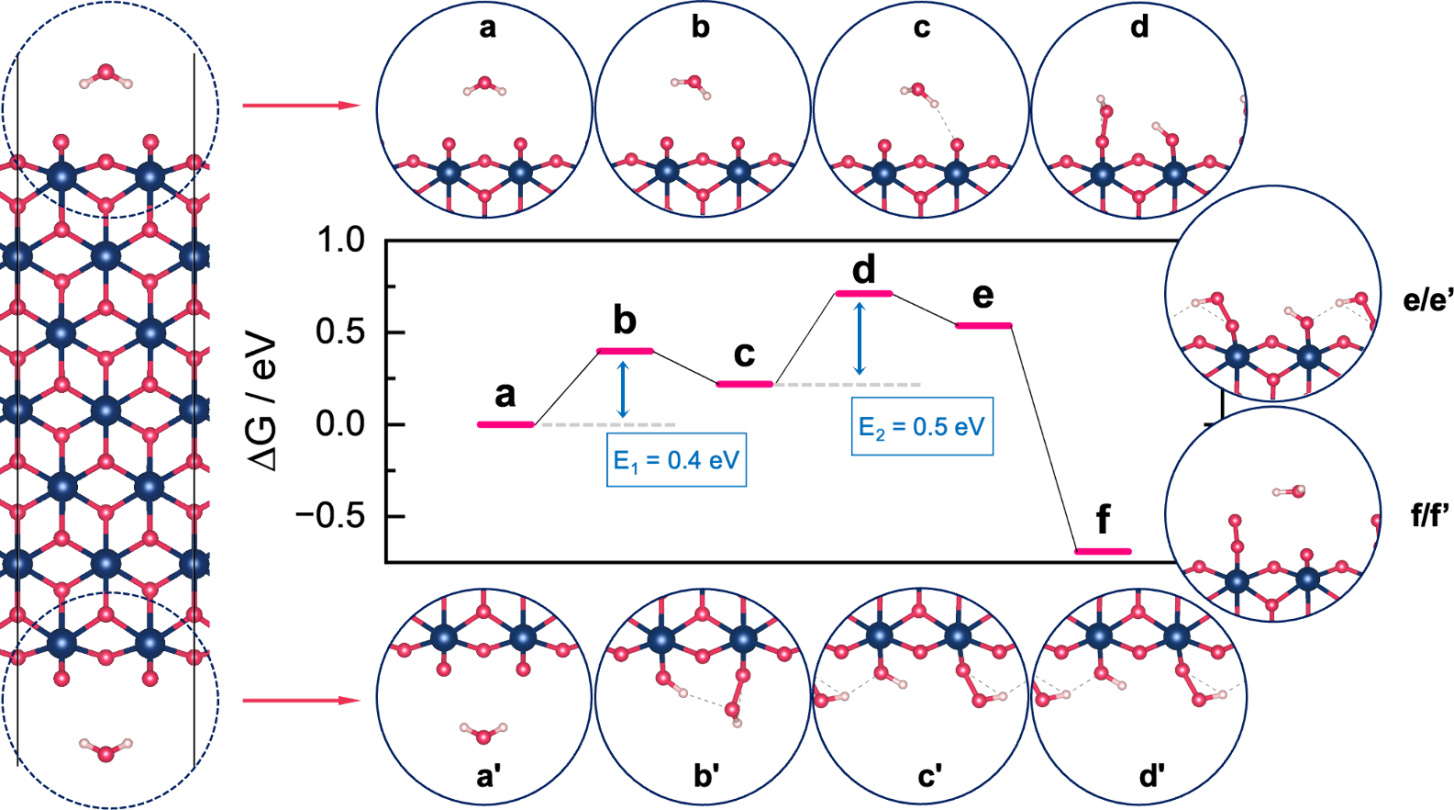
Calculated free energies of the reaction path for water oxidation on μ_1_-O referenced to the initial state; the sheet calculations have two sites (left), on the top and bottom edge, while the latter is marked with an apostrophe; since they did not react simultaneously, the structure of the edges are shown separately for a–d, for e–f the top edge is representative for both edges (see right-hand side); the activation energies for the O–O coupling are 0.4 eV for the bottom edge and 0.5 eV for the top edge (E_1,2_, a–e).

To verify that the reaction on μ_1_–O is feasible, we calculated the reaction barrier on a μ_1_-O site on the edge of an IrOOH nanosheet with two edges using the climbing image nudged elastic band method (Figure 5).^46^ We consider O–O coupling as the rate-determining step (detailed discussion in the SI) and found activation energies for between 0.4 and 0.5 eV. These barriers are low compared to the 0.6 eV in Ping et al. on IrO_2_(110) using implicit solvation^47^ and is expected to be lowered further using explicit solvation.^36^ Similarly to studies on IrO_2_(110),^36,47^ O–O coupling on IrOOH edges includes a proton transfer to a neighboring μ_1_-O site, which makes the reaction a nonconcerted electron transfer, or chemical reaction step.

To summarize the expectation from ab initio calculations, we can put on record that there are three distinct oxygen species on IrOOH nanosheets: μ_1_-O, μ_2_-O, and μ_3Δ_-O, in the order of increasing connectivity. μ_1_-O is distinct in that it is an oxyl that is highly reactive in O–O coupling. μ_2_-O and μ_3Δ_-O have similar hole character, but due to their different connectivity, they have distinct chemistry: The basal plane μ_3Δ_-OH deprotonates at a lower potential than μ_2_-OH. The O K-edge white line positions of these three surface oxygen species are separated by more than 0.2 eV, which is larger than the typical experimental resolution. Knowing the spectroscopic fingerprint, we can now test the relationship between oxygen connectivity and electrochemical behavior with operando X-ray spectroscopy.

### Connecting Oxygen Species to Electrochemical Currents

**Figure 6:**
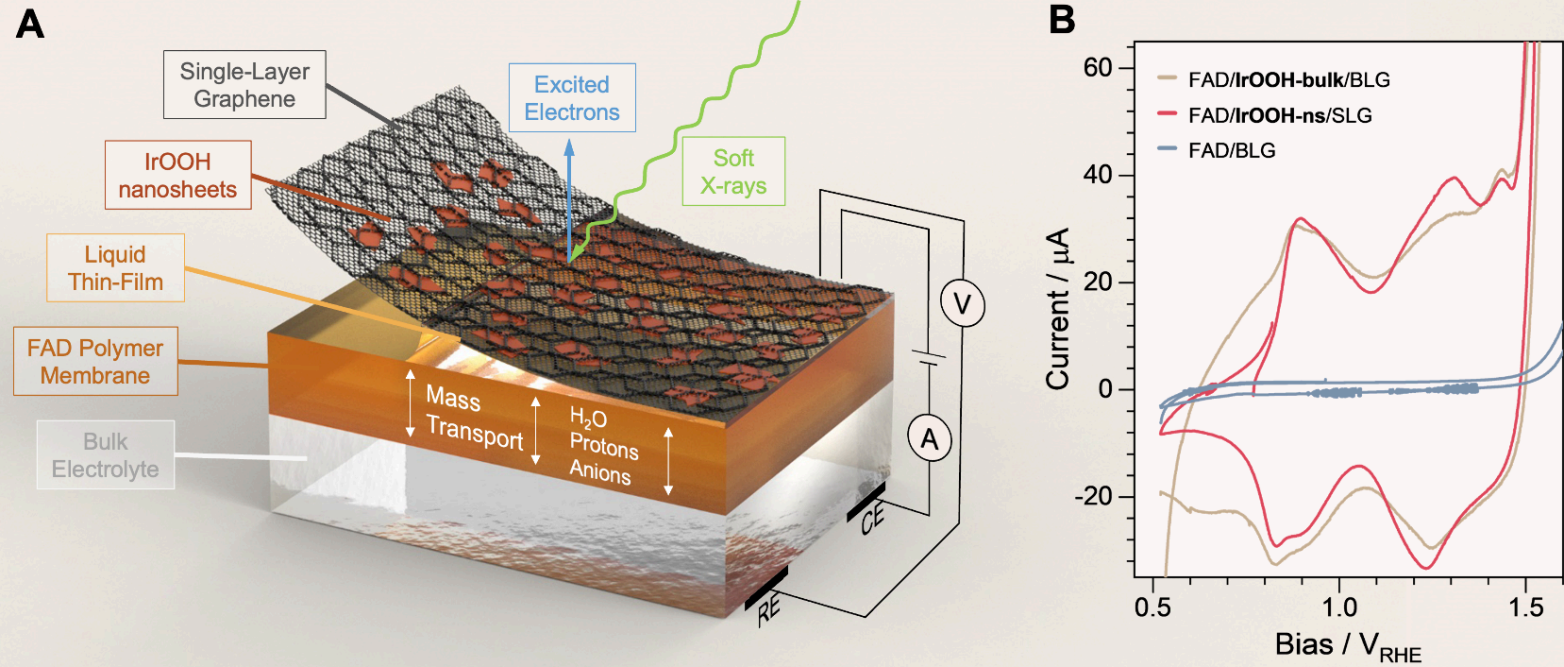
(A) Artistic rendering of the operando samples, comprising the bulk electrolyte (bottom) in which the Ag/AgCl reference electrode and the Pt counter electrode are immersed (bottom right), the FAD polymer membrane through which water, protons, and anions can pass (sandwiched orange layer), and the graphene double layer (BLG, black) with attached IrOOH nanosheets (red platelets); the top is facing vacuum, but the evaporation barrier of BLG leads to a liquid thin film between graphene and the polymer membrane; X-ray spectroscopy is measured from the top using soft X-rays (green arrow); electrons (blue arrow) are detected with a differentially pumped NAP-XPS system. (B) Cyclic voltammetry at 10 mV/s of FAD/BLG without catalyst (blue) and with IrOOH catalyst (beige for drop-casted IrOOH powder and red for IrOOH nanosheets).

The samples for operando X-ray spectroscopy were made from a PEM (FAD by Fumatech), the nanosheets, and a graphene blanket (Figure 6; more information is available in the SI). This arrangement is in contact with bulk electrolyte in which the counter and reference electrodes are immersed. Liquid electrolyte (including sulfate ions and protons) can pass through the FAD membrane and form a thin electrolyte layer between the membrane and the working electrode (Figure 6A).^48^ This setup allows surface sensitive X-ray spectroscopy at a biased solid–liquid interface by using a near-ambient pressure X-ray photoelectron spectroscopy (NAP-XPS) setup. A sample architecture consisting of an FAD membrane, IrOOH nanosheets, and a single layer of graphene (SLG), which is in contact to 0.1 M H_2_SO_4_, will be referred to as (0.1 M H_2_SO_4_)/FAD/IrOOH-ns/SLG in the following. From TEM (Figure S45), SEM (Figure S46), and AFM (Figure S48) analysis, we expect aggregates and particles to contribute 40% of the intensity in X-ray absorption and photoelectron spectroscopy (see SI), the remaining intensity stems largely (>80%) from single or double layers.

CVs of bulk IrOOH and IrOOH nanosheets are the first test. Both CVs have oxidation features at 0.9 V_RHE_, 1.3 V_RHE_, and at 1.4 V_RHE_, that mostly differ in their width. The more expressed oxidation feature at 1.3 V_RHE_ of the exfoliated material might be caused by more μ_2_–OH species in defected basal planes being exposed to the electrolyte. The delayed reduction of bulk IrOOH to below 0.9 V_RHE_ might originate from mass transport limitations. As in the prediction, the three oxidation events separate four phases with the difference that deprotonation of μ_3_-OH was predicted 0.4 V earlier than that in the experiment, which will be discussed later in the text. In the following, we will investigate each redox transition by operando spectroscopy.

**Figure 7:**
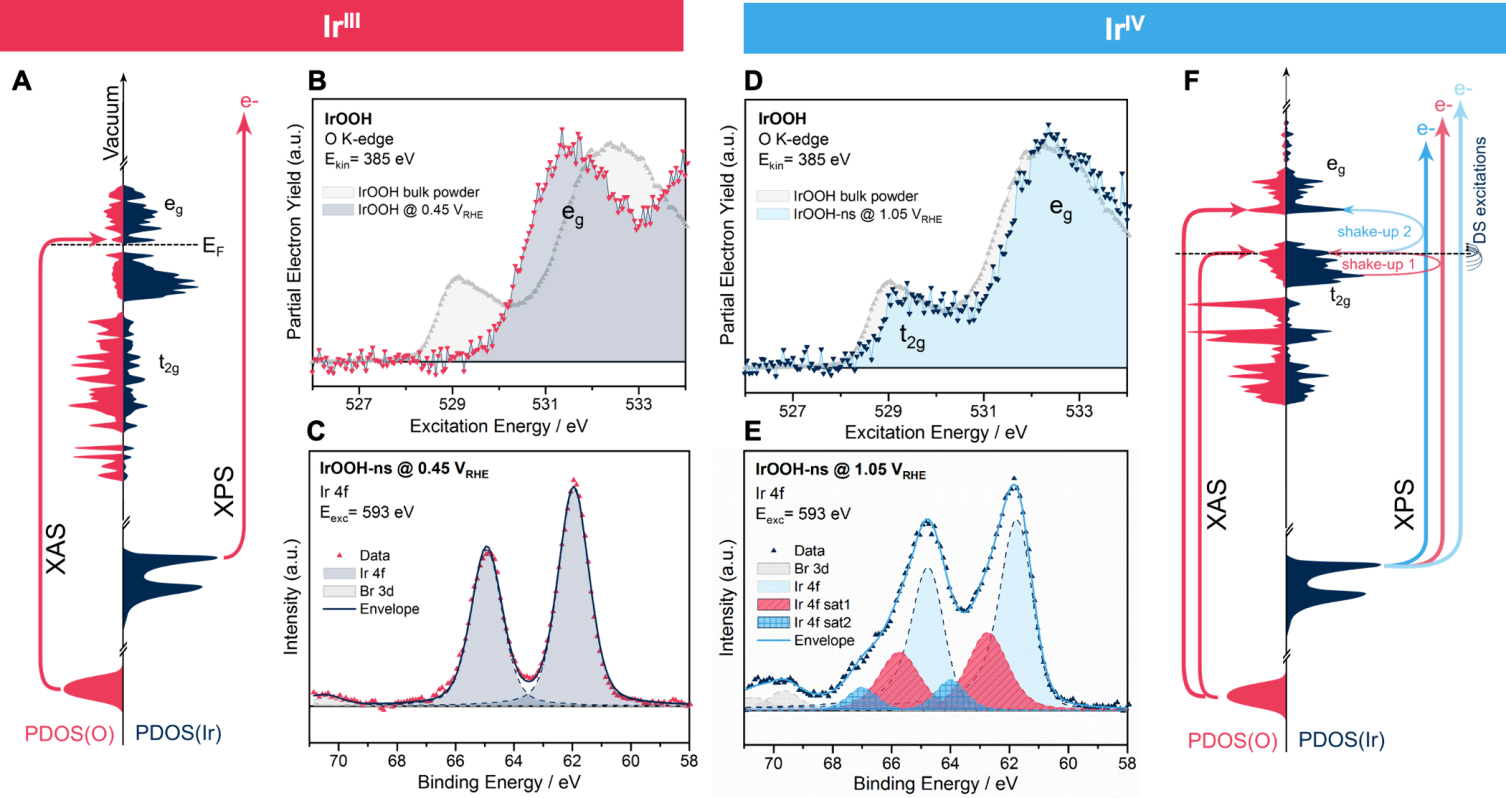
Operando (B,D) O K-edge absorption spectroscopy measured in a partial electron yield normalized to the maximum e_g_ intensity and (C,E) operando Ir 4f X-ray photoelectron spectroscopy (XPS) of (0.05 M H_2_SO_4_)/FAD/IrOOH-ns/SLG at a potential of (B,C) 0.5 V_RHE_ and (D,E) 1.1 V_RHE_; the excitation schematics for X-ray spectroscopy on sheets of Ir^III^OOH and Ir^IV^OO are given in part (A) and (F) of the figure; absorption at 532 eV originates from excitations from O 1s into unoccupied states with e_g_ symmetry, while the white line at 529 eV originates from an excitation into unoccupied states with t_2g_ symmetry (left part of (A,F)); the photoelectrons in XPS experience energy loss from coexcitations from occupied t_2_- into unoccupied t_2g_- and e_g_-like states, resulting in an asymmetric Doniach–Sunjić line shape (light blue), and two shakeup satellites (red and intense blue); a Shirley-type background was subtracted from the shown Ir 4f XP spectra.

The first transition occurs at about 0.9 V_RHE_ and is predicted to be the transition from a semiconducting state (Ir^III^) to a conducting state (Ir^IV^). The CV (Figure 6B) switches from a tapered shape to a broader capacitance region, typical for metal–insulator transitions.^49,50^Figure 7B,C shows the spectroscopic measurements at 0.5 V_RHE_ in a well-equilibrated state, i.e., at negligible currents (Figure S49). The Ir 4f spectrum shows two symmetric peaks with a spin doublet separation of 3.0 eV, the expected intensity ratio of 4:3, and an Ir 4f 7/2 peak position of 62.0 eV (see Table S8). Noticeable is the reverse core level shift in comparison with rutile-type IrO_2_ appearing at 61.8 eV.^15,51^ The O K-edge at 0.5 V_RHE_ (Figure 7B) shows a single contribution at 531.5 eV, which originates from excitation from the core level into unoccupied e_g_-like states (Figure 7A) and is in line with prediction (Figure 4, structure I).

At 1.1 V_RHE_, the Ir 4f spectrum is clearly asymmetric toward higher binding energies (Figure 7E). Similar to previous work,^15^ we fitted using an asymmetric Doniach–Sunjić line shape^52^ with two shakeup satellites (see Table S8). The BE of the Ir 4f 7/2 is 61.7 eV, and the spin doublet separation is 3.0 eV. Fits of the first shake up satellite (Table S8) show an energy loss of ∼1 eV, which matches the difference between the Fermi energy and a sharp feature in the occupied PDOS (Figures 7F and S30). The second satellite (sat2 in Table S8) resides ∼2 eV above the main line, which matches an excitation from the Fermi energy into a peak in the density of states at 2 eV above the Fermi energy (Figures 7F and S30). The experimental K-edge has a clear absorption white line slightly above 529 eV and a second broader feature at 532.5 eV (Figure 7D). The spectrum agrees well with what is expected from the calculations (Figure 4, structure II).

The IrOO(H) nanosheets reach the state of ∼Ir^IV^ at 1.1 V_RHE_, and the basal planes are deprotonated, but the remaining μ_2_-OH_*x*_ and μ_1_-OH_*x*_ edge can still be oxidized. The respective PCETs are predicted to occur at 1.3 and 1.5 V_RHE_, as observed in the CVs of IrOOH-ns (Figure 6B). At 1.3 V_RHE_, deprotonation of μ_2_-OH and μ_1_-OH_2_ sites leads to a formal oxidation state of Ir^IV^ (Figure 4, structure III) and additional deprotonation of μ_1_-OH leads to formally Ir^VI^ (Figure 4, structure IV).

**Figure 8:**
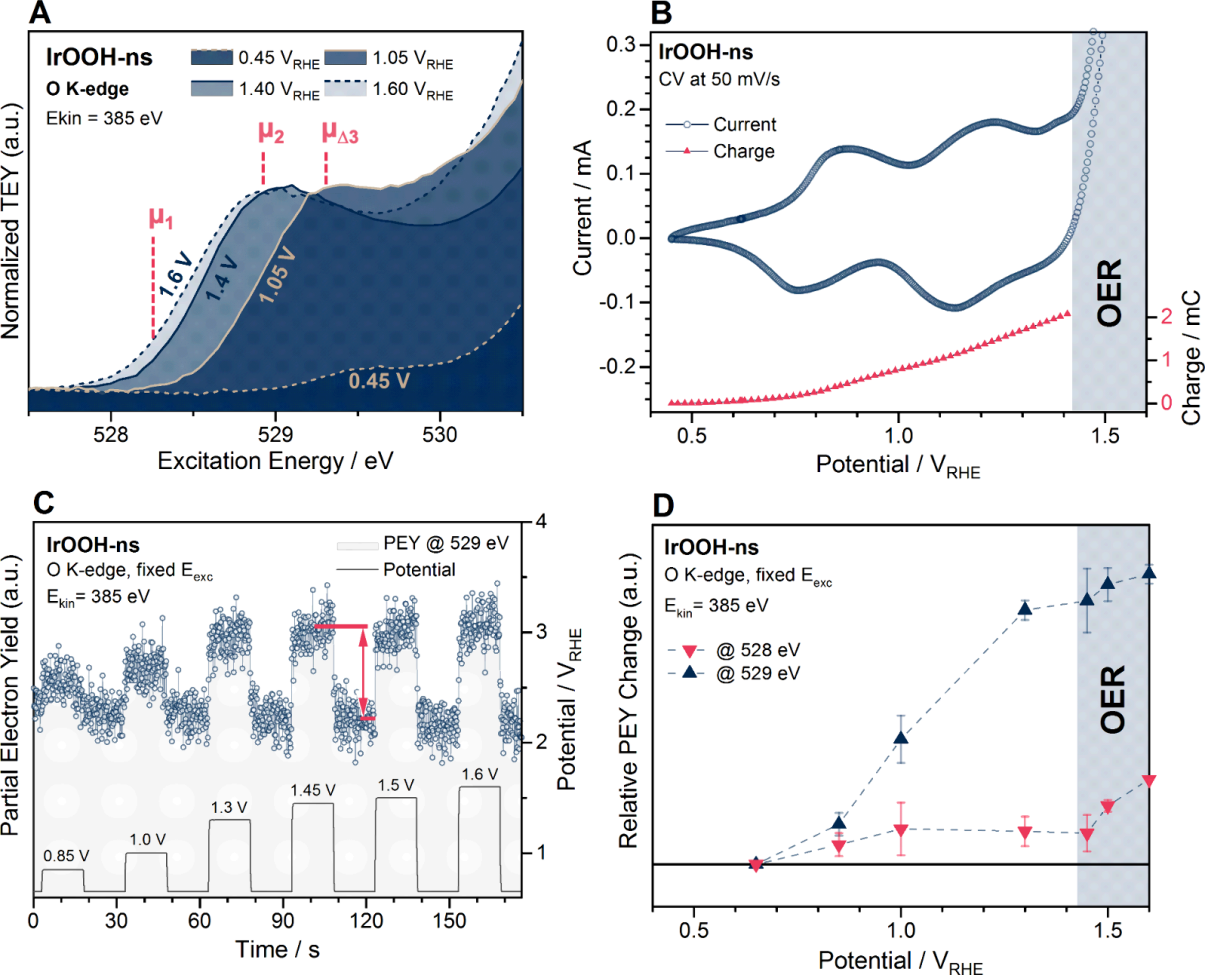
Electrochemical operando spectroscopy of 0.05 M H_2_SO_4_/FAD/IrOOH-ns/SLG: (A) O K-edge absorption spectra normalized to the peak intensity of the white line (except at 0.45 V_RHE_); (B) cyclic voltammogram and the respective integrated charge up to the onset of the OER; (C) exemplary potentiodynamic X-ray absorption showing the applied potential on the bottom (right axis) and the response of the partial electron yield signal at *E*_exc_ = 529 eV (left axis); (D) signal difference relative to the signal intensity at 0.65 V_RHE_, as indicated in red in part (C); the signal at 528 and 529 eV in (D) is an average from data of four measurement positions; a tail of the 528 eV white line is captured at 529 eV.

Experimental K-edge spectra of nanosheets at the OER onset (1.4 V_RHE_) and under operation conditions (1.6 V_RHE_) are shown in Figure 8A. At the onset of the OER, μ_2_-O contributes at ∼528.7 eV and the corresponding Ir 4f spectrum is further broadened (Figure S53). During the OER, the O K-edge of the nanosheets shifts further toward lower *E*_exc_. These changes are qualitative because the spectra were recorded at different measurement positions to avoid beam damage and thus had to be normalized for comparison (see caption of Figures 7 and 8). Quantification could be obtained for a given species and measurement position with potentiodynamic X-ray absorption, for which the white line intensity was tracked over multiple potential steps (Figure 8C) and positions. The statistical result is shown in Figure 8D. The signal intensity at 529 eV contains contributions from μ_2_-O to μ_1_-O, increases with the first oxidation wave at 1.2 V_RHE_, and saturates approaching the OER. The signal at 528 eV captures the resonance characteristic for μ_1_-O (see Figure 4). It keeps increasing into the OER, suggesting an active role of oxyl in the reaction.

Operando spectroscopy confirmed that the μ_*x*_-O deprotonation potential decreases with connectivity. It also confirmed the metal insulator transition from formally Ir^III^ to Ir^IV^ via deprotonation of the basal plane μ_3Δ_-O. Both μ_2_-O and μ_3Δ_-O are stable well below OER potentials, but with deprotonation of μ_1_-O oxygen starts evolving, as predicted by the strong hole character on μ_1_-O. Having linked the model with its electrochemical properties via ab initio methods and operando experiments, we now compare to other IOHs to be able to draw more general trends on how connectivity is linked to performance in the discussion.

### Comparisons between IOHs

**Figure 9:**
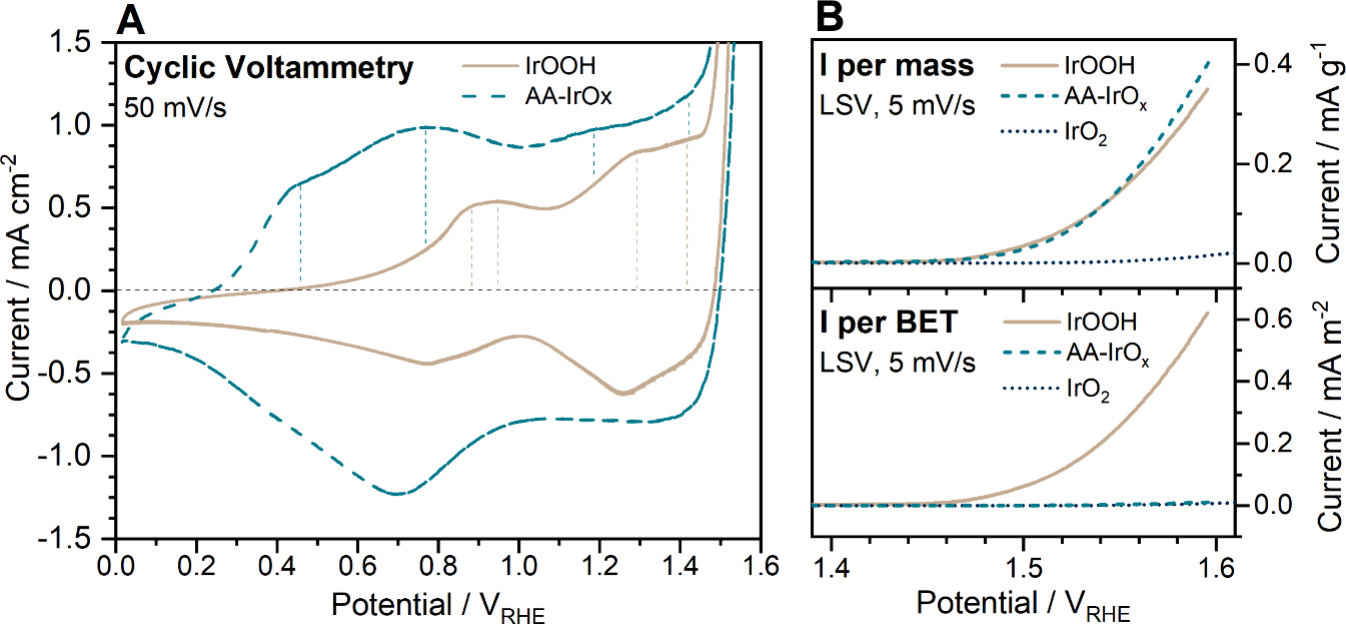
Electrochemistry in Ar-saturated 0.1 M H_2_SO_4_ of a polished glassy carbon rotating disk coated with a catalyst layer containing Nafion: (A) cyclic voltammetry of AA-IrOx and IrOOH and (B) their respective linear sweep voltammetry in comparison to rutile-type IrO_2_; normalization to mass is given on the top, and normalization to the BET surface area is given on the bottom.

As a comparison to IrOOH (nanosheets), we use well-studied IOH examples, an amorphous IOH powder from Alfa Aesar (AA-IrO_*x*_) with elements of crystalline hollandite-type and rutile-type phases,^7,15,23,24^ calcined rutile-type IrO_2_ powder, and anodized Ir nanoparticles (Ir NPs), as in ref (25). For Ir NPs, we expect the formation of anodic IOHs with an amorphous and hydrated structure.^53^ The electrochemical behavior of the powdered materials was tested on a rotating disk electrode (Figure 9, see SI for details).

The mass activity (Figure 9B) is similar for IrOOH and AA-IrO_*x*_ but when normalized to the Brunauer–Emmett–Teller (BET) surface area of the powders (see Figure 9B and SI), IrOOH shows a much larger intrinsic activity. The activity per surface area of crystalline IrO_2_ is comparable to that of AA-IrOx. The same trend is obtained when normalizing to electrochemical surface area, i.e., capacitance (see Figure S41). The intrinsic activity of exfoliated and powder IrOOH is not compared, due to an uncertain weight loading of nanosheets.

The amorphous AA-IrO_*x*_ has a first oxidation feature at 0.45 V_RHE_ and the CV is wider than that of IrOOH (Figure 9A). Calcined IrO_2_ has fewer oxidation features and a large capacitance region (Figure S40). An analysis of the capacitance at 1.0 V_RHE_ (see Table S6) yielded 26 mF/cm^2^ for AA-IrO_*x*_, 4 mF/cm^2^ for IrOOH, and 0.5 mF/cm^2^ for IrO_2_. However, we cannot exclude contributions from the redox events. The last oxidation feature before OER at 1.4 V appears similar in IrOOH and AA-IrO_*x*_ but is shifted to 1.6 V_RHE_ for IrO_2_.

Assuming octahedral coordination and the limited Ir–O speciation, the electrochemical differences suggest that the varying connectivity influences the deprotonation potentials of Ir–O species and their reactivity.

**Figure 10:**
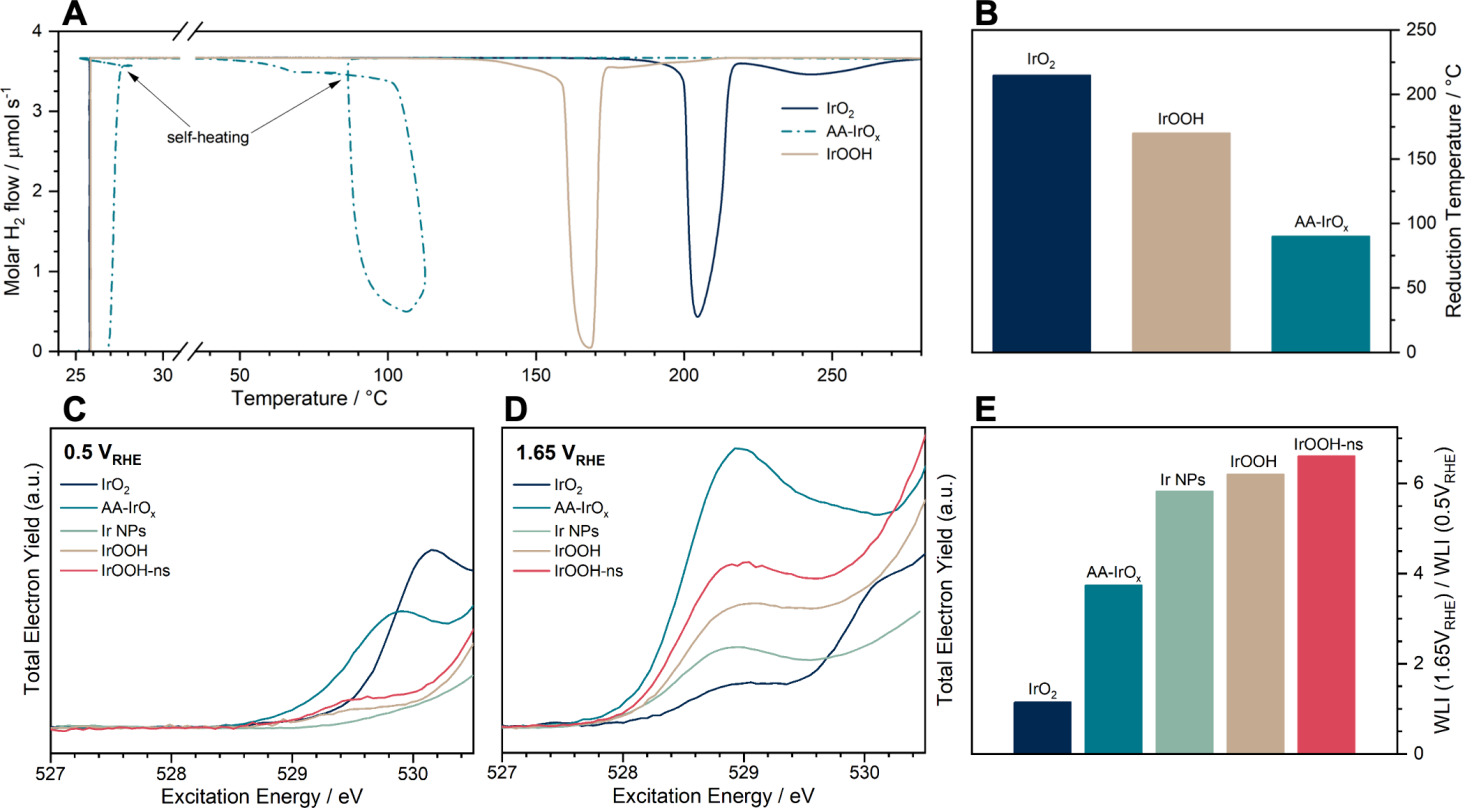
(A) Temperature-programmed reduction in a flow-through reactor; the measured molar flow of hydrogen downstream of the reactor is shown during a linear heating ramp from room temperature to 300 °C at 6 K/min; reactor inflow at 100 mL/min and 5.0% hydrogen in argon is 3.7 μmol/s; (B) temperature at which peak reduction has finished, marked by a sharp increase of downstream hydrogen content; (C,D) operando O K-edge white lines normalized to the edge jump at 550 eV, i.e., all oxygen species, at 0.5 V_RHE_ and 1.65 V_RHE_, respectively; (E) ratio of the white line intensity (WLI) at 1.65 V_RHE_ and 0.5 V_RHE_ integrated between 527 and 530.5 eV, showing the relative reducibility of all oxygen species.

Another consequence of varying connectivity is a larger variation in the bond strengths. We do a bond strength analysis with peak reduction temperature of TPR in H_2_. The molar flow of H_2_ at the outlet during a heating ramp from room temperature to 300 °C is given in Figure 10A. The peak reduction event of the roughly 25 mg of powder is finished at 90 °C for AA-IrO_*x*_, 170 °C for IrOOH, and 215 °C for IrO_2_, as summarized in Figure 10B. The two reduction events of AA-IrOx (dash-dot curve in Figure 10A) at room temperature and 75 °C are exothermic, causing a nonlinear temperature ramp (see Figure S15) and loop functions (Figure 10A). A similar effect is observed in IrOOH, but it is less pronounced (Figure S15). The peak reduction temperatures can be used, in a first approximation, as a measure of bond strength. A Redhead type analysis of first order gave a Ir–O bond strength of 1.44 eV for IrO_2_ and 1.33 eV for IrOOH using the programmed heating rate of 6 K/min.^54^ For the strongly exothermic reaction on AA-IrO_*x*_, we used the measured heating rate at a peak reduction of 58 K/min, which gives 1.06 eV for the Ir–O bond strength. This analysis shows that a larger distribution of bond strengths, i.e., lower connectivities, leads to a lower temperature of disintegration. As OER dissolution studies found the same trend,^17−20^ Ir–O bond strength hence seems to relate with oxidative stability. This makes sense given the covalent nature of the Ir–O bonds. However, the inverse behavior of oxidative dissolution and bond strength raises the question if connectivity mostly influences stability via bond strength distribution or also via the IOHs electrochemical ability to reduce or oxidize via PCETs.

**Figure 11:**
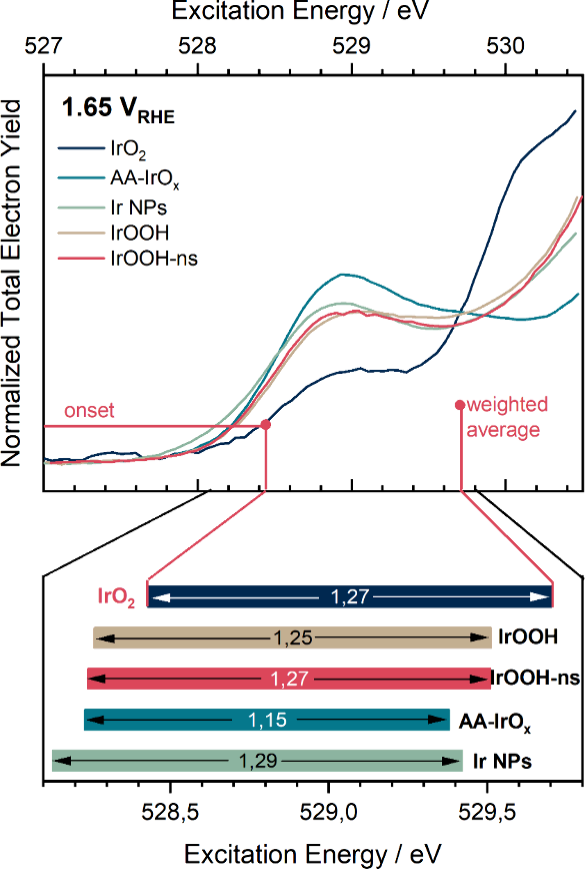
Average and the extreme hole character on oxygen in the OER of different IOHs. (Top) O K-edge white line at 1.65 V_RHE_ with the total intensity between 527 and 530.5 eV normalized to unity; (Bottom) floating bar graph; the average hole character on oxygen marks the right limit of the bars and is the intensity-weighted average excitation energy; the number of oxygen species with extreme hole character was evaluated by the excitation energy at the onset at an intensity of 5.7 × 10^–3^ and marks the left limit of the bars; the distance between these marking points, or the length of the bar, is noted at the center of each bar.

We investigated the effect of the bias on electrochemical reducibility (Figure 10C–E) and oxidizability (Figure 11) with operando spectroscopy at extreme potentials (0.5 and 1.65 V_RHE_). The electrochemical reducibility is measured by the relative WLI between 527 and 530.5 eV stepping from 1.65 to 0.5 V_RHE_. The WL spectra and the resulting values are given in Figure 10C,D, and E, respectively. The calcined bulk IrO_2_ is not reducible, as expected from single-crystal studies.^55^ It is followed by AA-IrOx, which is known to have crystalline domains of rutile-type and hollandite-type phases,^7,15,23,24^ and anodized iridium nanoparticles. Dissolution studies also concluded that more amorphous oxides have greater absolute dissolution. IrOOH is an outlier in this respect, as it is highly crystalline and reducible.

Electrochemical oxidizability is gauged with the average hole character on oxygen at 1.65 V_RHE_. To obtain this measure, we first normalize to all the integrated absorption intensity between 527 and 530.5 eV—carbonaceous oxygen species and water are not expected to contribute much intensity in this window^25^—and then evaluate the average excitation energy weighted by the absorption intensity. The average hole character increases in the order of IrO_2_ < IrOOH ≈ IrOOH-ns < Ir NPs < AA-IrO_*x*_, with AA-IrO_*x*_ being the most oxidized on average. Similarly, the respective Ir 4f spectra (Figure S53) become increasingly asymmetric in the order IrO_2_ < AA-IrO_*x*_ < Ir NPs < IrOOH ≈ IrOOH-ns, suggesting highly oxidized sites in IrOOH, AA-IrO_*x*_, and Ir NPs. This ordering shows that a lower connectivity allows for higher average oxidation states.

If the higher average oxidation states on oxygen explain reactivities, we evaluate the onset of the normalized white lines in Figure 11, a measure of the quantity of μ_1_-O oxyl species. When it comes to the number of oxyl species (onset), the order is IrO_2_ < IrOOH ≈ IrOOH-ns ≈ AA-IrO_*x*_ < Ir NPs. The span of the bars is similar for all IOHs where one dominant phase is expected, i.e., the crystalline IrOOH and IrO_2_. In addition, the nanoparticles with mixed metallic and amorphous phases have a similar span. The span of the bar is only smaller for AA-IrO_*x*_ which is a mixed amorphous and crystalline phase.^24^ Hence, we found that IrOOH, AA-IrO_*x*_, and Ir NPs during the OER have highly oxidized oxygen species that are expected to be active, but the average oxygen in AA-IrO_*x*_ and Ir NPs is more oxidized. The onset identifies IrO_2_ as the least active (compare Figure 9) but cannot distinguish between IrOOH and AA-IrO_*x*_. We will return to the meaning of this finding in the discussion.

## Final Discussion

This study is intended to narrow the structure gap between atomistic models of iridium oxohydroxides (IOHs) and their function as catalysts in acidic water oxidation. Paramount to achieving this goal was to determine the atomic and electronic structure of the model compound IrOOH precisely and evaluate the chemical behavior of interfacial species in comparison with other IOHs. We will first evaluate the success regarding structure, electronic structure, and site chemistry of IrOOH in the following and then move on to more general conclusions about IOHs below.

### Atomic Structure of IrOOH

The structure was determined using PXRD, PDF, and TEM (Figure 1 and 2). The results for all structural probes agreed well with ordered, crystalline nanosheets that when stacked, can best be described by a heterogenite-2*H* structure. The Ir–O bond lengths and Ir–Ir distances are within 1% before and after exfoliation, chemical etching, or 50 CVs between 0.35 and 1.65 V_RHE_ (Figure 2), indicating structural robustness. IrOOH owes this stability to a dense Ir–O bonding network of trivalent pyramidal μ_3Δ_ oxygen species. Unlike typical μ_3_-O in rutile-type IrO_2_, the μ_3Δ_-O in IrOOH exists in a protonated form (μ_3Δ_-OH), which allows formal iridium oxidation states of + III throughout the nanosheet. The hydrogen adsorption is in fact 0.67 eV on μ_3Δ_-O and 0.21 eV on μ_3_-O on a IrO_2_(110) surface, based on our DFT calculations. The bulk loses interlayer hydrogen atoms and oxidizes to ∼IrO_1.5_(OH)_0.5_.

### Electronic Structure of IrOOH

The electronic structure of IrOOH is expected to be a semiconductor from simple considerations of crystal field splitting, DFT calculations (Figure 3), and evidence from UV–vis spectroscopy (Figure S18). However, X-ray spectroscopy (Figures 3, S19, and S22) and DFT revealed that IrO_1.5_(OH)_0.5_ is heavily doped with hydrogen vacancies, at the expense of lattice distortion (Figures S4 and S25).

If a bias is used to fix the chemical potential of electrons and influence the oxidation state of Ir via PCETs, operando measurements on IrOOH nanosheets (or bulk) agreed well with a gapped Ir^III^OOH at 0.45 V_RHE_ and a conducting Ir^IV^OO at 1.05 V_RHE_.

At 0.45 V_RHE_, we observed a reverse core level shift of Ir 4f (vs IrO_2_) and a symmetric line shape. Final state effects are the most likely explanation for these observations. The core hole is screened by ligand charge transfer, leading to symmetric line shapes typical for semiconductors. The binding energy of Ir^III^OOH is blue-shifted compared to the Ir^IV^OO, due to more efficient conduction band screening in Ir^IV^OO. The O K-edge has one broad feature at 532 eV originating from excitations into unoccupied e_g_-like states (Figure 3 and 7A), across the gap, and no white line indicating unoccupied t_2g_-like states.

At 1.05 V_RHE_, the Ir 4f line becomes asymmetric due to excitations across the Fermi level within t_2g_-like states (Figure 7F) and an O K-edge white line appears at 529 eV. Both are consistent with unoccupied t_2g_-like states facilitating conductivity. Support for a metal–insulator transition also comes from the tapered shape of the CVs (Figure 6B) below 0.9 V_RHE_.

Further oxidation beyond Ir^IV^ pushes the material into a negative charge transfer regime, in which hole character increasingly resides on oxygen instead of iridium.^32,33,35^ In response, the O K-edge absorption white line becomes more intense and shifts to lower *E*_exc_ in calculations and experiment (Figure 4 and 8 and refs (20,25)). The respective Ir 4f spectrum is broadened further toward higher BEs (Figure S53), supporting the assertion that iridium centers are further oxidized.^33^

### Ir–O Speciation in IrOOH and Their Chemistry

The μ_*x*_-O speciation and their chemistry on IrOOH were tested with TPR, electrochemistry, operando spectroscopy, and CO titration. We start the discussion with μ_3Δ_-O. It has a remarkably low *E*_exc_ reminiscent of an electron-deficient μ_2_-O species active in CO oxidation,^15,23^ but unlike the latter, μ_3Δ_-O does not oxidize CO at room temperature (Figures S36–S39). The Löwdin charges of μ_3Δ_-O and μ_2_-O are, in fact, similar. In H_2_ TPR, IrOOH reduces at about 170 °C (Figure 10), not far from rutile-type IrO_2_, which reduces at 215 °C. The estimated Ir–O bond strengths are 1.4 and 1.3 eV, respectively. Since IrO_2_(110) has been shown to have “extraordinary stability” toward cathodic reduction,^55^ the similar bond strength of IrOOH at comparable hybridization suggests a good chemical stability of basal plane μ_3Δ_-O, i.e., limited cathodic dissolution. The μ_3Δ_-OH sites deprotonate in the first redox reaction at about 0.9 V_RHE_ (Figure 4), oxidizing the sheets from +3 to +4.

Further μ_*x*_–O speciation was done with a combination of CVs (Figures 6, 8, and 9), a calculated surface phase diagram (Figures 4 and S32), and operando spectroscopy. The PCET of μ_2_-O(H) explains the second redox couple at 1.3 V_RHE_, adding absorption intensity at ∼528.7 eV (Figures 4 and 8). The PCET at μ_1_-O(H) explains the third redox couple at 1.45 V_RHE_ (Figures 4 and 8). The order of the oxidation waves agrees well with other amorphous or crystalline IOHs.^20,25^ However, a one-to-one comparison of the transition potentials to calculations is still challenging, due to the influence of surface protonation and solvation in the ab initio model, which has been shown to influence the above energy of PCETs in calculations.^36,47,56^ The relatively small unit cell containing two μ_1_-O molecules at the edge of the sheet aggravates these influences with respect to the total energy. For example, the μ_3Δ_-O below 0.5 V_SHE_ shows an absorption white line that is not observed by the operando experiment, suggesting that a dense hydrogen bonding network suppresses the white line originating from nonprotonated μ_3Δ_-O. Donating hydrogen bonds are in fact known to suppress white line intensity.^25^ The lower energy from this solvation could also explain why the deprotonation potential of the basal plane is predicted at 0.5 V_NHE_ (Figure 4) instead of the 0.9 V_RHE_ in experiment (Figures 6 and 8), though the use of implicit electrolyte is also a likely source of discrepancy; explicit electrolyte increases and broadens the deprotonation window over rutile-type IrO_2_.^20,36^

In the experiment, the deprotonation of μ_1_-OH shows no saturation and increases only with the onset of the OER (Figure 8), suggesting an active role in water oxidation. This stands to reason, since μ_1_-O has a Löwdin charge distinct from μ_2_-O and μ_3_-O (Figure 4) and μ_1_-O has been predicted to have radical character on IrO_2_(110) at formally Ir (+5.33),^25,32^ as well as on IrO_2_(111) and IrO_2_(001).^33^ Iridium at IrOOH nanosheet edges can formally oxidize to +6 (Figure 4) and exhibits an intense white line at low *E*_exc_ (Figures 4, 8, and 11), both predictive of reactivity in O–O bond formation.^25,33,36^ A consequence of μ_1_-O being the active site is that the OER occurs at the edges of sheets, not the basal plane. The same was found on ruthenium nanosheets^57^ and on cobalt platelets.^58^ Since charge storage influences the reaction barrier of O–O coupling,^32,33,35,36^ an interesting aspect is that on nanosheets, unlike on bulk materials, the main part of the charge storage would occur on sites that are not directly involved in the reaction.

### Summary on IrOOH

**Figure 12:**
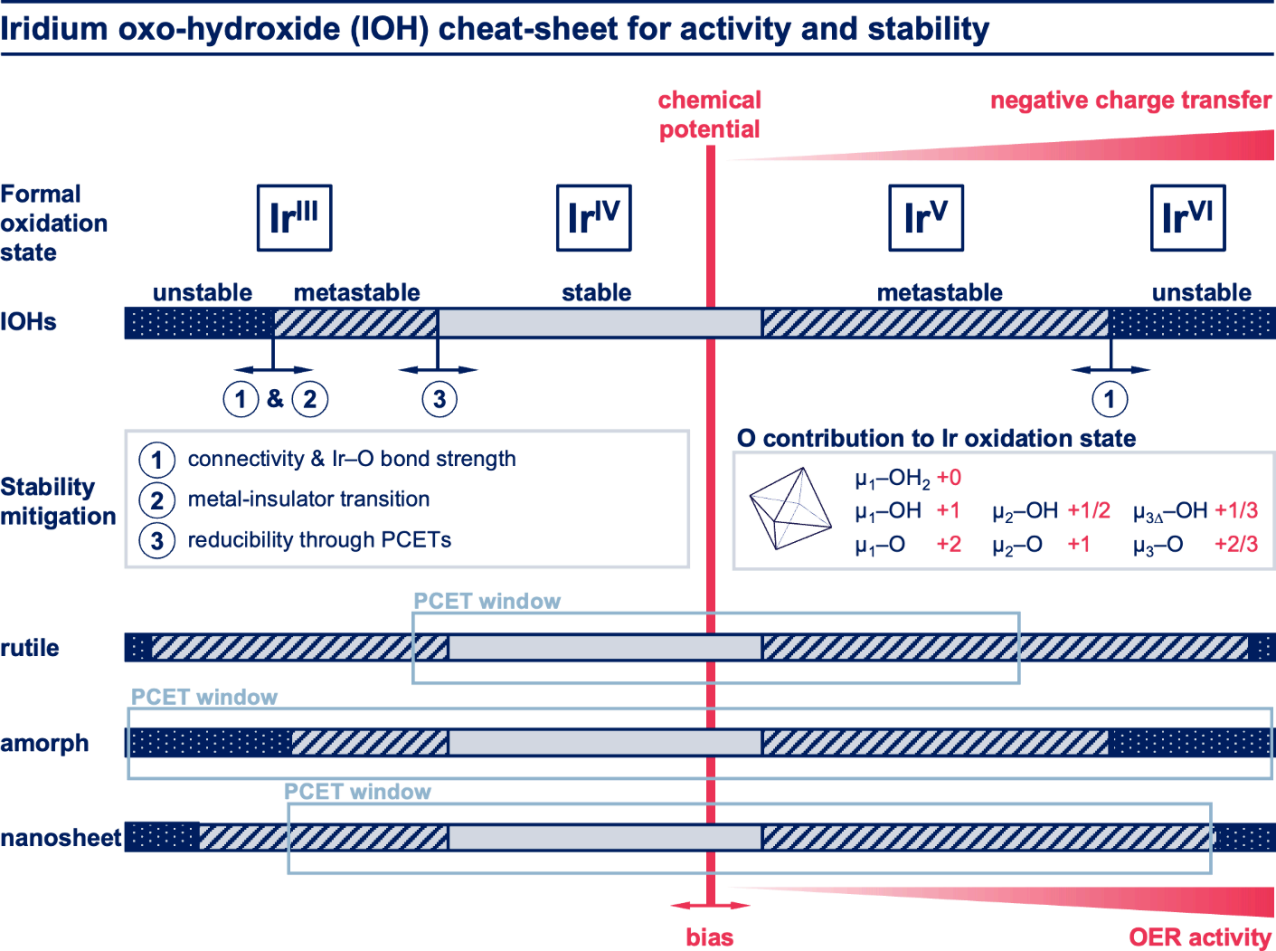
“Cheat-sheet” how activity and stability in IOHs depend on connectivity; the formal oxidation state of iridium atoms (oxygen fixed at −2) are given on the top; since iridium is almost always octahedrally coordinated, the formal oxidation state can be calculated from the connectivity of iridium in combination with the protonation state of octahedrally coordinated oxygens, as given in the light blue box in the middle right; iridium at the IrOOH edges, for example, is connected to three μ_3Δ_, two μ_2_, and one μ_1_ oxygen species; when these oxygens are fully protonated, the formal oxidation state is 3*1/3 + 2*1/2 + 1*0 = +3, and when they are fully deprotonated, it is 3*2/3 + 2*1 + 1*2 = +6; if the coordination is assumed to not change, the connectivity determines the maximum and minimum formal oxidation state that can be reached with proton-coupled electron transfers (PCETs), or electrochemical (de)protonation, as marked with light blue rectangles in the bottom part; a bias (shown as a red vertical bar) can thus control the formal oxidation state of iridium; since iridium and neighboring oxygens are strongly hybridized, the additional hole character tends to reside increasingly on oxygen beyond Ir(+4)—a negative charge transfer regime (red wedge on top) is entered; this leads to increasingly OER-active oxygen species (red wedge on bottom); if no bias is applied, formal oxidation states typically return to something between 3.5 and 4.5, marking the stable region (light blue of horizontal bars), the bias then either forces the material into a metastable state (hatched part of horizontal bars), or, depending on the bond strength, into an unstable state (dark blue part of horizontal bars); the metastable regime can be extended by the metal–insulator transition, alleviating pressure from the bias to reduce, and by the reducibility of the structure. Please note that the sharp borders between stable, metastable, and unstable regions indicate trends and are not precisely determined by experiment.

Our atomistic model of IrOOH nanosheets connects a wealth of experimental and theoretical findings, a sign of a reliable model. But only the comparison to other IOHs will show if the structure–function relationship of IrOOH nanosheets is exceptional or follows general trends. To find out how far our findings on the IrOOH nanosheets can be generalized, we will compare different IOHs with respect to their thermodynamic stability, their PCET windows, and their OER activity. Figure 12 will serve as a summarized guide of this discussion.

### Thermodynamically Stable Oxidation States in IOHs

The spontaneous formation of hydrogen defects in bulk IrOOH at ambient conditions (Figure 3) hints at Ir^III^ in the [Xe]6s^0^5d^6^ configuration being metastable or unstable. Along similar lines, a computational study found Ir_2_O_3_ being considerably less stable than IrO_2_, due to relativistic effects.^59^ If we then also consider the lack of an experimental proof for an Ir^III^ IOH stable at ambient conditions, it stands to reason that that IOHs thermodynamically favors an oxidation state above +3 in the most stable octahedral coordination,^6,60^ unless stabilized by other elements, bias, solvation, or similar. Further reduction of Ir^III^ is expected to be even less stable, due to an additional cost of occupying antibonding e_g_-like states. However, it should be noted that both a mixed and average oxidation state are possible. In iridium dimers, mixed III–IV oxidation states could not be observed.^61^

The upper limit of iridium oxidation state is expected above Ir^IV^, where IOHs enter the negative charge transfer regime and form electrophilic oxygen species.^32,33,35^ The probe molecule CO can identify such species.^23,62^ In fact, AA-IrO_*x*_ with an average Ir oxidation state of ∼4.13 (Figure S22) oxidizes CO at room temperature (Figures S36–S39 and ref (23)), pointing toward metastability of those sites in ambient conditions. The active site is most likely μ_2_-O, which are known to oxidize CO below formally Ir (+5.33).^23,62^ IOHs thus become metastable between +4 and +5.33. The exothermic reaction of H_2_ or CO (Figure S36) at room temperature also point toward metastable domains in AA-IrO_*x*_.

As a rule of thumb, IOHs with octahedral coordination and no other elements involved thermodynamically prefer formal oxidation states between roughly +3.5 and +4.5 (Figure 12) and are metastable or unstable beyond these oxidation states, unless stabilized by solvation or other special circumstances. Manganese, for example, can stabilize Ir^VI^.^63^

### PCET Window in IOHs

How far a bias can change the oxidation state without altering the connectivity depends on the PCET window, which we define as the range of oxidation states that can be reached by interfacial species. Connectivity defines the width of this PCET window because the contribution to the formal oxidation state of (octahedrally coordinated) iridium follows the order μ_1_ > μ_2_ > μ_3_, as shown in the inset of Figure 12. It follows logically that the PCET window is larger for lower connectivities. For example, the prototypical IrO_2_(110) surface can reach a minimum oxidation state of +3.33 and a maximum of +5.33, defining the PCET window for a IrO_2_(110) model (gray box for crystalline IOHs in Figure 12). In comparison, a highly amorphous material with more μ_2_-O(H) and μ_1_-O(H) sites is expected to have a much larger PCET window that accommodates a wider variety of oxidation states (Figure 12). In effect, various IOHs showed similar electronic structures at moderate biases between ∼0.5 and 1.4 V_RHE_, despite expected differences in their connectivity. Find the equivalents of Figure 7 for other IOHs in Figures S50–S52 and for Figure 8A in Figure S53. However, the acidity of the surface sites, the oxidation states accessible via PCETs influence redox potentials. This is why CVs are sensitive to connectivities.^5,64,65^ The comparison of IrOOH and AA-IrO_*x*_ (Figure 9), for example, shows that the oxidation from the lowest oxidation state at 0 V_RHE_ and 1.1 V_RHE_ (Ir^IV^) involves more charge at lower potentials for AA-IrOx. This is consistent with more μ_2_ and μ_1_ species that allow for lower oxidation states via PCETs (Figure 12). Assignment of redox transitions can be guided by oxygen valence since O–H bond strength and oxidation potential increase with μ_1_ > μ_2_ > μ_3_.^25^

In a nutshell, smaller connectivities allow the adaption of a wider range of formal oxidation states through PCETs, i.e., a larger PCET window. The bias foremost controls the oxidation state or electronic structure, and depending on connectivity, the IOHs accommodate this request.

### OER Activity of IOHs

The barrier of O–O bond formation–the rate-determining step of the OER in acids–is known to depend strongly on the hole character on oxygen.^36,47^ Three trends increase the hole character on oxygen: first, a smaller valence μ_1_ > μ_2_ > μ_3_, second, a larger formal oxidation state on iridium, third the overall hole character at the (sub)surface.^32,33,35,36^ This means that IOHs with lower connectivities have more active sites than their crystalline counterparts, and they can be charged far into the negative charge transfer regime (Figure 12). And indeed, the mass activity of AA-IrOx is much higher than that of IrO_2_ (top Figure 9B).

When it comes to intrinsic activity of the surfaces, IrOOH outperforms both IrO_2_ and AA-IrOx (bottom Figure 9B). The advantage over IrO_2_ can be explained clearly by the three trends above. The IrOOH edge sites can oxidize to formal oxidation states of +6, which cannot be reached on typical surfaces of rutile-type IrO_2_.^20,33,66^ In addition, the μ_3Δ_-O on oxidized IrOOH surfaces have Löwdin charges as large as their μ_2_-O counterpart, which are known to influence the rate-determining reaction barrier.^36^ The comparison between IrOOH and AA-IrO_*x*_ is not as straightforward. Both IrOOH and AA-IrO_*x*_ have highly oxidized sites which should be equally active (Figure 11). One possible explanation is that the effect of the overall hole character in the (sub)surface on the reaction barrier works well in a conductive μ_3_-O framework^36^ but is more localized in amorphous structures due to lower connectivity and increased disorder.^67^ Another is that amorphous oxides have more sites with more hole character on oxygen, but also more sites with lower hole character on oxygen.^68^ This wider distribution leads to a less consistent contribution of neighboring hole character to the most active sites, as is the case for IrOOH. A third explanation could be the effectiveness of proton transfer to neighboring sites during O–O bond formation, which might be more scattered in amorphous structures.

In essence, the most active sites are oxyl μ_1_-O, a prerequisite for the formation of the −O bond in acidic OER. A lower connectivity creates more of such active sites, leading to large mass activity. Intrinsic activity of sites seems to also depend on the surroundings of the active sites and is harder to predict.

### Electrochemical Stability of IOHs

As established above, IOHs are largely thermodynamically unstable below +3.5 and above +4.5. A bias fixes the chemical potential of the electrons and thus can go beyond the limits with PCETs. If this leads to a metastable or unstable state, it depends on how high the barrier is to break an Ir–O bond. If the bonding framework is weak, the metastable region is narrowed, and the structure degrades sooner. The Ir–O bond estimated from the TPR reduction temperature is IrO_2_ > IrOOH > AA-IrO_*x*_ (Figure 12). This means IOHs with lower connectivity have a higher number of weak Ir–O bonds and, therefore, a narrower metastable region (Figure 12). This agrees with large dissolution rates for amorphous IOHs^17−20^ and with the finding from Lee et al. that amorphfous structures have more weak and more strong bonds.^68^ However, thorough dissolution studies on nanosheets are still needed, given that the stability of planar and pyramidal μ_3_-O is considerably different in manganese oxides.^69^

The bias should be particularly destructive outside the PCET window, where the only way to accommodate extreme oxidation states is the breaking of Ir–O bonds. This delivers an explanation for why—in relative terms—crystalline IrO_2_ suffers more from reductive dissolution than from oxidative dissolution when compared to amorphous IOHs.^19,20^ The PCET window of IrO_2_ does not accommodate oxidation states below +3.33 on the surface and +4 in the bulk, making the material more unstable toward reduction. This is aggravated by the bulk, which is fixed at +4 and stays conductive and thus cannot reduce the driving force for reduction (as indicated in Figure 12). A study on the reactive IrO_2_(100) surface indeed found that extended cycling amorphized the surface to accommodate more extreme oxidation states.^70^ In absolute terms, amorphous IOHs are much less stable toward reductive and oxidative dissolution, highlighting the dominant rule of connectivity and bond strength, which makes IrO_2_(110) surprisingly stable against reductive dissolution.^55^

An interesting consequence of the connection between PCET windows and stability is that the active site is dynamically entering unstable regimes during a reaction cycle. This leads to either reversible turnover or dissolution, depending on connectivity and bond strength of the active site’s bonding network.

IrOOH seems to combine the best of both crystalline and amorphous IOHs. The μ_3Δ_ sites provide large connectivity, which widens the window of metastability, and, unlike their μ_3_ counterparts, allow full reduction to a gapped/semiconducting Ir^III^ material, diminishing the destructive effect of reductive potentials.

In summary, the connectivity and bond strength distributions are linked,^68^ ultimately leading to correlated activity and stability, as suggested by other authors.^16−18,20^ However, the stability of the Ir–O bonding framework appears to be the most important effect for the overall dissolution rate, followed by size of the PCET window and reducibility to a gapped/semiconducting state at low potentials.

## Conclusions

The present study of IrOOH establishes structure–function relationships at the atomic level with a narrow gap between the model and experiment. We determined oxyl μ_1_-O on the nanosheet edges are the active sites during OER. The intrinsic activity of these nanosheet edge sites is outstanding, even compared to amorphous iridium oxohydroxides (IOHs). However, when comparing to other IOHs, IrOOH is an outlier in the common trade-off between activity and stability of noble OER catalysts.^16^ Their basal plane is a dense framework of trivalent pyramidal μ_3Δ_-O that provides both surface electron hole character comparable to that of μ_2_-O and a strong bonding network. IrOOH combines the best of crystalline and amorphous IOHs: they have highly active sites within a stable framework of μ_3Δ_-O bonds. A thorough study of nanosheet stability is, however, still missing.

Drawing on a wealth of research on IOHs and the present work, we compiled a set of simple rules to estimate the activity and stability for a given atomic model (depicted also in Figure 12). These simple rules include, but are not limited to

1. Lower oxygen valence (μ_1_ > μ_2_ > μ_3_) increases (A) O–H bond strength, (B) variance of formal iridium oxidation states, and (C) OER activity.
2. The formal iridium oxidation state is a good indicator of a site’s OER activity.
3. The bias foremost controls the oxidation state of the material, which accommodates the request via proton-coupled electron transfers (PCETs).
4. A higher connectivity creates a larger barrier for dissolution.
5. IOHs that fully reduce to gapped Ir^III^ diminish destructive effects of reductive potentials.

These simple rules are based on many advanced studies, but their application requires only an atomic model and simple bond counting, available to any scientist. The reverse process—from experiment to model—will also be easier with these rules. Simple electrochemistry, such as CVs, should be sufficient to gain insights into the atomic model of the catalyst.

## Outlook

Nanosheets seem to strike the ideal balance between activity and stability with almost complete metal utilization. However, despite first signs of long-term stability,^39^ more insights into nanosheet dissolution and detachment are needed to make the case for large-scale employment of nanosheets in PEM electrolyzers. Marrying the concept of nanosheets with nanoporous materials, such as hollandite IrO_2_,^24,65,68^ might increase iridium utilization even further.

We hope that our simple predictive rules will be used, criticized, and refined to empower future studies toward a more complete understanding of OER catalysts. It would be of particular importance to test if the rules extend to other noble metal catalysts and ultimately to other transition metal catalysts.

## Experimental Section

IrOOH powder was obtained from precursor K_0.75_Na_0.25_IrO_2_ by exchanging alkali cations in 1 M HCl for 5 days. The remaining potassium within the first 3 nm from the surface is 1.6 ± 0.3% with respect to iridium (see SI). The precursor was synthesized from one equivalent of iridium powder, 2.6 equiv of K_2_CO_3_, and 0.4 equiv of Na_2_CO_3_, which were heated in air at 850 °C for 120 h. The synthesis follows the procedure of a previous report,^71^ but the temperature treatment of the precursor was altered in order to avoid a hollandite-type impurity phase (see SI). Exfoliable material was obtained from a precursor synthesized with a different heating procedure, namely, 900 °C for 15 h. Exfoliation was done using tetrabutyl ammonium hydroxide (TBAOH), ultrasonication, and separation by centrifuging the suspensions. Samples for operando spectroscopy were produced in a chemical transfer method using FAD membranes by Fumatech (Bietigheim-Bissingen), bilayer- or single-layer graphene (SLG) from Graphenea (San Sebastian), and (drop-casted) catalyst material. The samples were used on an operando cell made of PEEK and held in place by a boron-doped diamond coated niobium lid, as described elsewhere.^48^ The obtained nanosheets on a free-standing SLG were inspected in a transmission electron microscope (TEM) on a Quantifoil gold grid.

XRD measurements were performed in Bragg–Brentano geometry on a Bruker AXS D8 Advance II theta/theta diffractometer, using Ni filtered Cu Kα_1+2_ radiation. Total scattering measurements were collected using a Stoe Stadi-P diffractometer with AgKα1 radiation, a Ge(111) Johann monochromator, and a DECTRIS Mythen 1K detector in a Debye–Scherrer geometry. Their PDF was fitted with simulated PDFs from a structure model based on XRD results using the PDFgui and PDFgetX3 software.^72,73^ To index the patterns of the 2*H*-heterogenite structure, we used entry 56288 of the Inorganic Crystal Structure Database (ICSD), replacing Co by Ir.

All ab initio DFT calculations performed in this work are done with the Quantum EPSRESSO package.^74,75^ The generalized gradient approximation is used in form of Perdew–Burke–Ernzerhof (GGA-PBE) type functionals from the Standard Solid State Pseudopotential (SSSP) library^76−79^ to treat the exchange and correlation energy. Cutoffs for kinetic energy and charge density are set to 60 and 480 Ry, respectively, and the Marzari–Vanderbilt type cold smearing was set to a width of 0.01 Ry. A gamma-centered reciprocal grid with an equivalent distance of 0.2 Å^–1^ between adjacent k-points was used throughout the work in order to achieve the k-grid consistency of various structures. The convergence threshold for electronic self-consistency was set to 1 × 10^–8^ Ry. Atomic geometries and lattice constants (in-plane lattice constants only in 2D structures) were fixed to the experimental lattice constants (ionic relaxation) and relaxed until the total energy and forces converged within the thresholds of 10^–6^ and 10^–4^ atomic units, respectively. The method could lead to slightly compressive strain, since it tends to produce bonds longer than in experiments. In the case of 2D and edge structures, a spacing of at least 18 Å was ensured in the nonperiodic directions.

A home-built setup was used for flow-through TPR experiments using quartz reactor tubes inside a tube furnace. Gas analysis was done by a thermal conductivity detector, which was calibrated by using reference gas mixtures. Electrochemical measurements (excluding in situ studies) have been done with a rotating disk made of glassy carbon and a thin catalyst coating (40 μg catalyst and ∼8 ng Nafion per cm^2^).

TEM measurements were conducted using a Thermo Fisher Scientific Talos F200X, operated at 200 kV. Special care was taken to minimize beam damage, which was evident from ring-like patterns in the SAEDs after damage and degrades into nanometer-sized particles (see Figure S43).

Ex situ spectroscopy and in situ measurements in CO were both recorded at the BelChem beamline at the BESSY II synchrotron facility. Electrochemical in situ and operando experiments have been done at the ISISS beamline, also at BESSY II. We used Pt wire and an Ag/AgCl electrode as counter and reference electrode, respectively. XAS spectra have been processed with a self-written Python script, and XP spectra were fitted using the CasaXPS software.^80^ The energy calibration method for O K-edge absorption has an error below ±0.05 eV; the calibration error of XP spectra is below ±0.15 eV and below ±0.1 eV in the case of in situ Ir 4f spectra of IrOOH nanosheets, which were calibrated by second order excitations in situ. The probing depth for partial electron yield and XPS range between 2.5 and 3.5 nm (three times the inelastic mean free path in IrOOH from the TPP-2 M^81^ formula). The probing depth of total electron yield is typically around 10 nm.^82^

For further information on experimental procedures and methods, please visit the SI.

## Supporting Information Available

Detailed analysis of X-ray diffraction measurements, temperature-programmed reduction in hydrogen, and room temperature CO oxidation in a reactor and NAP-XPS setup with accompanying spectroscopy; optical data; quantification of X-ray absorption measurements of the O K-edge and Ir L_2,3_-edge; details on the input structures and calculated energies, density of states, and absorption spectroscopy from ab initio calculations, as well as calculated reaction barriers; complementing electrochemical characterization; further characterization of nanosheet stacking, layer height, and crystal structure from TEM, AFM, and SEM; and in situ spectroscopy of multiple IOHs, as well as XPS fitting parameters (PDF)
