# Supplementary material for: Atomic Insights into the Competitive Edge of Nanosheets Splitting Water
Source: J Am Chem Soc. 2024 Sep 25;146(40):27886–902. doi: 10.1021/jacs.4c10312 (PMC11467904; doi:10.1021/jacs.4c10312)
Supplement: Supplementary file 1 — ja4c10312_si_001.pdf [file ja4c10312_si_001.pdf]

Supporting Information of

Atomic insights into the competitive edge of nanosheets splitting water

Lorenz J. Falling[1,2*], Woosun Jang[1,3], Sourav Laha[4,5], Thomas Götsch[1], Maxwell W. Terban[5], Sebastian Bette[5], Rik Mom[1,6], Juan-Jesús Velasco-Vélez[1,7], Frank Girgsdies[1], Detre Teschner[1], Andrey Tarasov[1], Cheng-Hao Chuang[8], Thomas Lunkenbein[1], Axel Knop-Gericke[1], Daniel Weber[5,9], Robert Dinnebier[5], Bettina V. Lotsch[5], Robert Schlögl[1], Travis E. Jones[1,10*]

1 Fritz Haber Institute of the Max Planck Society, Berlin 14195, Germany

2 School of Natural Sciences, Technical University Munich, 85748 Munich

3 Integrated Science & Engineering Division, Underwood International College, Yonsei University, Incheon 21983, Republic of Korea

4 Department of Chemistry, National Institute of Technology Durgapur, Mahatma Gandhi Avenue, West Bengal-713209, India

5 Max Planck Institute for Solid State Research, Stuttgart 70569, Germany

6 Leiden Institute of Chemistry, Leiden University, 2300 RA Leiden, The Netherlands

7 Experiments division, ALBA Synchrotron Light Source, Cerdanyola del Vallés, Barcelona 08290, Spain.

8 Department of Physics, Tamkang University, Tamsui 251, Taiwan

9 Wallenberg Initiative Materials Science for Sustainability, Chemistry and Chemical Engineering, Chalmers University of Technology, 41296 Gothenburg, Sweden

10 Theoretical Division, Los Alamos National Laboratory, Los Alamos, NM 87545

* To whom correspondence should be addressed: lorenz.falling@tum.de and tejones@lanl.gov

Synthesis of IrOOH and exfoliation

$K_{0.75}Na_{0.25}IrO_2$ was prepared following a similar procedure as in the previous report.[1] One equivalent of iridium powder was mixed with a flux containing 2.6 equivalent of K_2CO_3 and 0.4 equivalent of Na_2CO_3 and heated in a corundum crucible in air at 850 °C for 120 h. The product was quenched to room temperature and quickly transferred to an argon filled glovebox to obtain a grey product containing a mixture of $K_{0.75}Na_{0.25}IrO_2$ crystallites and remaining flux.

The product was treated with 1 M HCl (1 mL mg^{-1}) for 5 days to remove the excess flux and exchange the alkali cations with protons. The acid was exchanged every day. The remaining residue was washed with deionized water and dried at room temperature for overnight to obtain IrOOH crystallites with a tinge of pink. We used powder X-ray diffraction (PXRD), scanning electron microscopy (SEM) and energy dispersive X-ray spectroscopy (EDX) to characterize the quality of the products. Alkali content after exchange is 0.5% for Na and 0.1% for K relative to Ir, as provided by inductively coupled plasma optical emission spectrometry, or ICP-OES.

To evaluate the alkali content at the surface, we integrated the K 3p and Ir 4f regions from XPS experiments of the IrOOH powder in UHV and corrected the integrals by the differential cross section [2] and the inelastic mean free path (IMFP) in IrOOH using the TPP-2M formula.[3] We omitted the analyzer transmission function, which we assume constant within the range of kinetic energies (386 eV and 433 eV). A linear background was used for better comparability of the integration between K 3p and Ir 4f. A statistical analysis of 6 measurements gave a 1.6±0.3% potassium content relative to iridium. This means within about 3 nm (3 x IMFP) of the surface there is at most one potassium atom on 50 iridium atoms, assuming homogeneous distribution. We did not find Na 2p signal despite similar sensitivity as K 3p. This means there is potassium enrichment at the surface or the integrated area contains other spectral contribution, hampering our analysis.

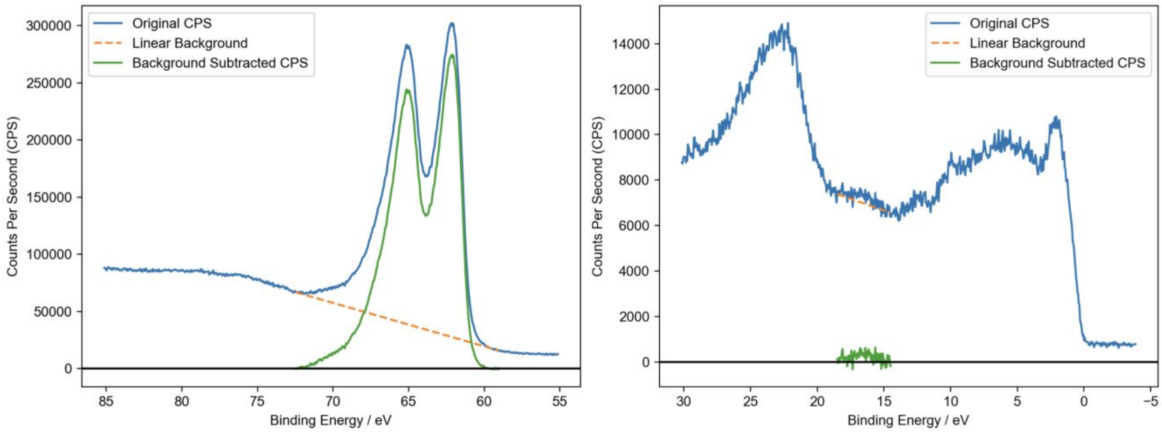

Figure S1: Exemplary X-ray photoelectron spectra of an IrOOH powder pellet at an excitation energy of 450 eV; a linear background was subtracted for the Ir 4f (left) and K 3p (right) and the difference integrated.

The IrOOH sample obtained following from the above procedure does not exfoliate. To prepare exfoliable IrOOH, the synthesis condition of the precursor had to $K_{0.75}Na_{0.25}IrO_2$ be modified.[4] The mixture of iridium powder, K_2CO_3 and Na_2CO_3 (in the molar ratio of 1 : 2.6 : 0.4) was heated to 900 ^{o}C for 15 h in air and was quenched to room temperature. The resulting product was treated with 1 M HCl and washed with deionized water to obtain the exfoliable bulk IrOOH.

The exfoliable bulk IrOOH powder was dispersed in a 10 mM aqueous solution of tetrabutyl ammonium hydroxide (TBAOH) with a molar ratio of IrOOH : TBAOH = 1 : 2 and ultrasonicated for 30 min to result in a blue colored IrOOH nanosheet suspension. The unexfoliated residues were separated by centrifuging the suspension at 1000 rpm for 30 min. The supernatant suspension was further centrifuged at 15000 rpm for 30 min to make all the nanosheets precipitate at the bottom. The TBAOH solution was removed and the remaining nanosheets were redispersed in isopropanol and were drop casted onto single layer of graphene (SLG) on copper foil obtained from Graphenea (San Sebastian). Since the redispersion is not entirely homogeneous, we lose the information about the deposited weight and hence intrinsic activity compared to IrOOH powder.

Powder X-ray diffraction

The powder X-ray diffraction (PXRD) measurements were performed in Bragg-Brentano geometry on a Bruker AXS D8 Advance II theta/theta diffractometer, using Ni filtered Cu $K\alpha_{1+2}$ radiation and a position sensitive energy dispersive LynxEye silicon strip detector. The sample powder was filled into the ca. 0.5 mm deep and 20 mm diameter recess of a zero-background silicon single crystal sample holder so that the smooth surface of the powder bed was at the same level as the sample holder surface. The XRD data were analyzed by whole powder pattern fitting according to the Rietveld method as implemented in the DIFFRAC.TOPAS software (version 5, Bruker AXS, 1999-2014).

In **Figure S2** we compare three different stacking geometries of the same sheet structure. The brucite-like 1*T* structure (**Figure S2**) has a simple AA stacking order. Within the limitations imposed by the anisotropic nature of the peak broadening, all major reflections of the pattern are matched reasonably well. However, as the inset of **Figure S2A** shows, two additional minor reflections are not reproduced by this model. While the original IrOOH publication [1] suggested a hollandite side phase, we use the heterogenite-2*H* type superstructure with AB stacking. Both the main reflections and the minor superstructure reflections can be simulated (**Figure S2B**). This is in analogy to the structure reported for the precursor $K_{0.75}Na_{0.25}IrO_2$.[1] Finally, in the case of the heterogenite-3*R* structure with an ABC stacking, the majority of the simulated reflections does not agree with the observed diffraction pattern, thus ruling out this possibility. Within the limits of the method, we could not detect any hollandite byphase with the updated synthesis recipe (see above), confirming the effectiveness of the treatment. However, we do find weak reflections (e.g. at 10° 2θ) that can be attributed to minor amounts (1 wt- %) of rutile type IrO_2 (**Figure S5**).

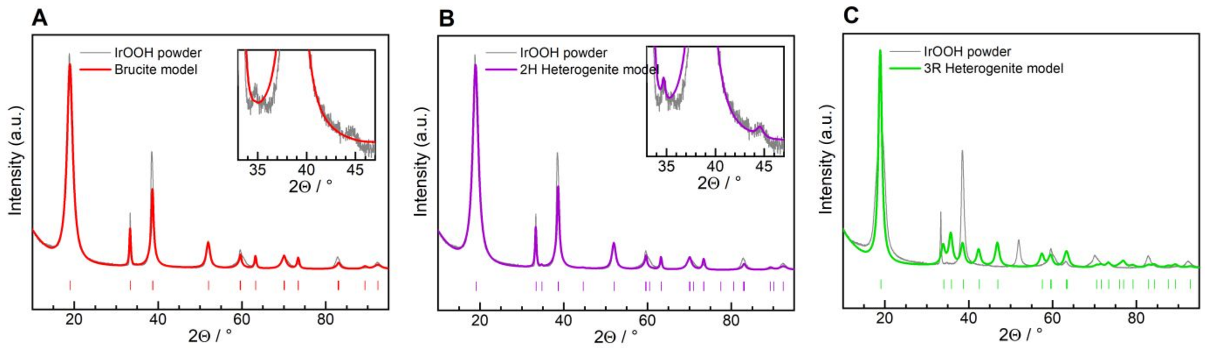

Figure S2: Powder Cu $K_{\alpha 1}$ X-ray diffraction of IrOOH (gray) compared with an A) brucite-1T B) heterogenite-2H, and C) heterogenite-3R model.

Concerning the origins of the observed anisotropic peak broadening, several explanations are possible. Apart from a probably platelet-like aspect ratio of the crystalline domains or anisotropic micro-strain, some contribution by stacking disorder between the layers seems likely. The peak profile of the main reflection in the stacking direction of the IrOOH layers shows a complex structure, which seems to be composed of at least three components (**Figure S3**), which requires further refinement.

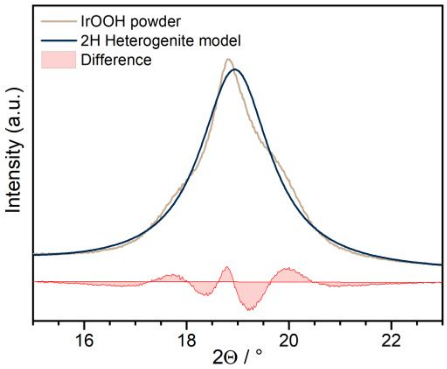

Figure S3: 002 reflection of IrOOH from powder Cu $K_{\alpha 1}$ X-ray diffraction (beige) and the difference (red) to a heterogenite-2H model (dark blue).

Due to the cell metrics and lattice symmetry only the 100 type reflections appear as non *l*-containing diffraction lines. These peaks exhibit a very sharp profile (**Figure S4a**), whereas diffraction lines like 101, 104 or $2\bar{1}2$ are significantly broadened (**Figure S4a**). This is an indicator for the presence of stacking fault disorder.[5] In addition, the 002 diffraction line is significantly broadened and, at close inspection (inset **Figure S4b**), reveals that the reflection is degenerated into three Bragg peaks. Any attempt to include these peaks and additional reflections underneath the broadened peak into a new indexing process of the powder pattern, failed. As other attempts to include these reflections into lateral and/or horizontal supercells also failed, we believe that the degeneration of the 002 diffraction line is attributed to the presence of domains, showing slightly different interlayer distances.

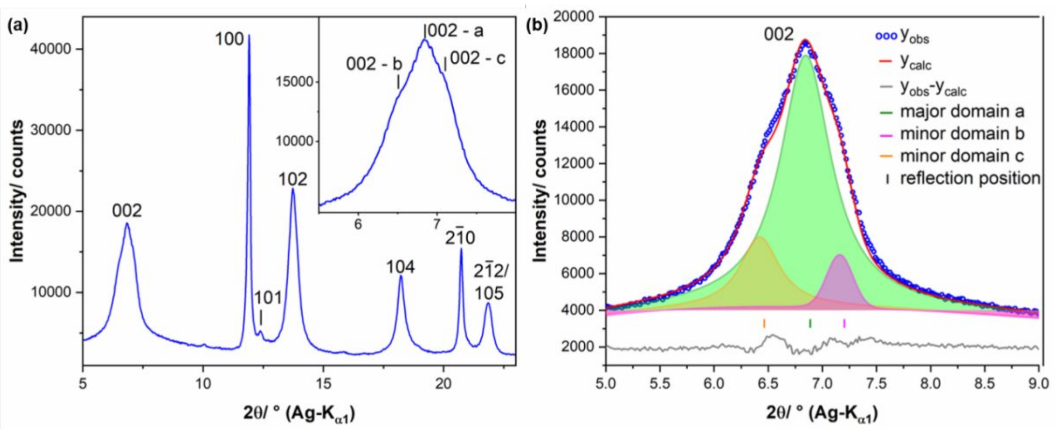

Figure S4: (a) Ag $K_{\alpha 1}$ XRPD pattern of heterogenite type IrOOH including selected reflection, the 002 reflection, which is split into three Bragg peaks, is presented in the inset, (b) Refinement of the 002 reflex using three isostructural heterogenite type structure with individual c-axes.

As the 100 and $2\bar{1}0$ reflections are neither degenerated nor broadened, they are most likely intergrown in a commensurate way. Hence, including three related heterogenite-type crystal structure with constrained a-lattice parameters seems to be straightforward. If simple domain size related peak broadening algorithms are used assuming an isotropic spherical shape, this will not lead to a satisfying refinement of the XRPD, as the usage of three constrained structure with individual c-axes would lead to a degeneration of the 104, as well, which cannot be observed.

Accordingly, we conclude that the intergrown domains haven a smaller and a larger interlayer distance, which we will call “minority domains”. They have a very limited vertical extension despite exhibiting a larger lateral extension. This leads to a highly 2θ-depending broadening and damping of the diffracted signal. For minimization of the parameter space, we modelled this by using artificially high isotropic thermal displacement parameters. The stacking fault disorder, we observed for the majority domain was phenomenologically described by using the Stephens model.[6] In order to avoid an over interpretation of the data available, we are not going to make any statement on the kind and degree of stacking fault disorder present. According to final refinement the majority domain is present with a weight fraction of 71 wt-% and an interlayer distance of 9.3105 Å, the minority domains are apparent with a weight fraction of 19 wt-% and an interlayer distance of 9.9217 Å and a weight fraction of 9 wt-% and an interlayer distance of 8.9041 Å (**Figure S5**).

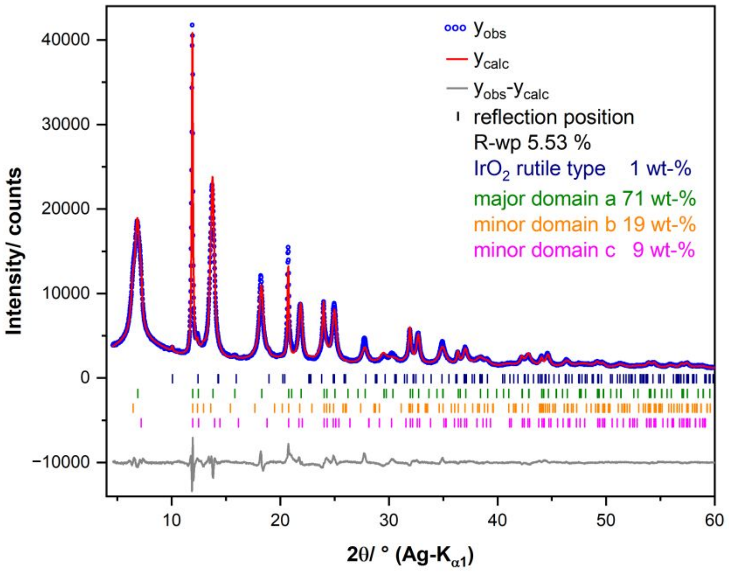

Figure S5: Graphical result of the final Rietveld Refinement of IrOOH using three isostructural, heterogenite-type phases with individual c-axes, Rwp = 5.53 %.

The degeneration of the interlayer distance can be associated with the presence of different ion sizes in the same lattice, i.e. r(Ir+3) = 0.68 Å, r(Ir+4) = 0.625 Å). Analyses on the cathode material $Li(Ni_{0.80}Co_{0.15}Al_{0.05})O_2$ [7] during charging and discharging revealed, that the lattice expansion, which is related to the oxidation and reduction induced changes in the cation radii of the transition metals, rather effects the vertical than the lateral dimension. In consequence the minority domain with the larger interlayer distance may exhibit an excess of Ir^{+3} and the minority domains with a smaller interlayer distance an excess of Ir^{+4} in relation to the majority domains. However, DFT of IrOOH structures with hydrogen defects found increasing lattice spacing with increasing iridium oxidation state, so a definite assignment is not possible based on our evidence. Both DFT and XRD confirm that the lateral lattice constants are less variable with oxidation and hydrogen vacancies, as the 100 reflex remains extremely sharp and DFT predicts only a small contraction of the lateral lattice constant a (**Figure S25**).

Due to the complex microstructure of the sample and the adverse trend from DFT calculations, we recommend to interpret the results of the XRPD refinement qualitatively.

Pair distribution function (PDF) analysis

Total scattering measurements were collected using a Stoe Stadi-P diffractometer with AgKα1 radiation (λ = 0.55941 Å, 22.162 keV), a Ge(111) Johann monochromator, and a DECTRIS Mythen 1K detector in Debye-Scherrer geometry. Samples were loaded into 0.2 mm ID glass capillaries and measured at

room temperature, over a range of 2θ = 2–127.73°, with 0.495° step size and 200 s counting time per step. Background scattering intensities from the capillary and air were measured and subtracted. Correction and normalization of the integrated total scattering patterns were carried out, using PDFgetX3 [8] within xPDFsuite,[9] to obtain the total scattering structure function, F(Q), and the pair distribution function (PDF), G(r). For elastic scattering, $Q = 4\pi sin\theta/\lambda$, where λ is the x-ray wavelength and 2θ is the scattering angle.

Analysis of the PDF provides a useful method for interpreting structure information from total scattering data, which treats both Bragg and diffuse scattering equally.[10] A diffraction measurement over a wide range of momentum transfer and with good statistics is required to obtain suitable PDFs for structure analysis. In order to obtain the pair distribution function, the measured powder diffraction intensities are first normalized by the average form factor squared to obtain the total scattering structure function $S(Q)$, defined as

$$S(Q) = \frac{I(Q) - \langle f(Q)^2 \rangle + \langle f(Q) \rangle^2}{\langle f(Q) \rangle^2}$$

The experimental PDF, denoted $G(r)$, is the truncated Fourier transform of the reduced, total scattering structure function, $F(Q) = Q[S(Q) - 1]$, as

$$G(r) = \frac{2}{\pi} \int_{Q_{min}}^{Q_{max}} F(Q) sin(Qr) dQ\,,$$

where $G(r)$ is the magnitude of the scattering momentum transfer. In practice, values of Q_{min} and Q_{max} are determined by the experimental setup, and Q_{max} is often reduced below the experimental maximum to reduce the effects of low signal-to-noise in the high-Q region on the Fourier transformation. To aid in qualitative assessment of the long-distance structural correlations, a modification function can be applied to $F(Q)$ prior to Fourier transformation by,

$$G(r) = \frac{2}{\pi} \int_{Q_{min}}^{Q_{max}} M(Q) F(Q) sin(Qr) dQ$$

and,

$$M(Q) = \frac{sin(Qr_{ij})}{Qr_{ij}}.$$

$M(Q)$ is called a modification function, in this case a Lorch function,[11,12] which damps the intensity of $F(Q)$ to 0 at Q_{max}. This reduces the effects of termination and noise on the Fourier transform, which

suppresses non-structural high frequency oscillations in the PDF. A Lorch function with a Q_{max} of 20.25 Å^{-1} was used for all data.

The PDF gives the scaled probability of finding two atoms in a material a distance r apart and is relative to the density of atom pairs in the material. For a macroscopic scatterer, $G(r)$ is calculated from a known structure model according to

$$G(r) = 4\pi r[\rho(r) - \rho_0],$$

$$\rho(r) = \frac{1}{4\pi r^2 N}\sum_i \sum_{j\neq i} \frac{f_i f_j}{\langle f\rangle^2}\delta(r - r_{ij}).$$

Here, ρ_0 is the average number density of the material and ρ(r) is the local atomic pair density, which is the mean weighted density of neighbor atoms at distance r from an atom at the origin. The sums in ρ (r) run over all atoms in the sample, f_i is the scattering factor of atom i, $\langle f\rangle$ is the average scattering factor and r_{ij} is the distance between atoms i and j. In this study, Eqs. 4 and 5 were used to fit the PDF generated from a structure model to the experimental PDFs in using the program PDFgui.[13] The delta functions in Eq. 5 were Gaussian-broadened. PDF modeling was performed by adjusting the lattice parameters, atomic positions were allowed by symmetry, atomic displacement parameters (ADPs), correlated motion of neighboring atoms δ2, domain size (*sp-diameter*), a global scale factor, and phase-specific scale factors for multiphase fits. The refinements were run by minimizing R_w, calculated as

$$R_w = \sqrt{\frac{\sum_{i=1}^{n}[G_{obs}(r_i) - G_{calc}(r_i,P)]^2}{\sum_{i=1}^{n} G_{obs}(r_i)^2}},$$

which was used to quantify the goodness-of-fit for the model. Additional Rietveld refinements were performed using TOPAS v6.[14]

Results

Diffraction patterns measured by AgKα1 radiation were assessed prior to reduction and Fourier transformation to the pair distribution functions (PDFs). IrO_x shows sharp Bragg reflections indicating highly crystalline metallic iridium, which was confirmed by Rietveld refinement, **Figure S6**. A significant diffuse component indicates the amorphous IrO_x component which is highlighted by the background function in green. Rietveld refinement was also attempted on the IrO_2 sample for reference purposes in **Figure S7**. However, distortions in relative peak intensities and unindexed peaks indicate either a slightly different structure from pure Rutile-type IrO_2, or impurity phases in the sample.

Data reduction from the scattering intensities I(Q) to the reduced structure functions F(Q) and finally to the PDFs, G(r), are demonstrated in **Figure S8**. Single-phase structure refinements of Rutile-type IrO_2 to the PDF of IrO_2 and Heterogenite to the PDF of IrOOH were performed over a local structure range of 1-15 Å. Despite the distortions indicated by the diffraction patterns, the fits to the local structure are fairly good for what can be expected for laboratory PDF data, **Figure S9** and **Figure S10**. This indicates that the local structure of IrO_2 can be mostly described by the Rutile-type structuring, indicating that this is the predominant nature of local atomic packing in the sample. The agreement of Heterogenite structure to IrOOH also indicates that despite long range disorder, this material consists of Heterogenite-type layers. Finally, for the IrO_x sample, a multiphase refinement was performed consisting of metallic iridium, Hollandite-type, and Rutile-type IrO_2, **Figure S11** and **Figure S12**. The fit is good, indicating that the amorphous component of IrO_x consists of a structure with characteristics of the local packing in both Hollandite and Rutile-type IrO_2, in agreement with the results of Willinger et al.[15] The refinement resulted in an amorphous structure with the characteristic of Hollandite to Rutile local structure in an atomic ratio of approximately 80:20. Average Ir-O and Ir-Ir atom-pair distances are given in **Table S1**.

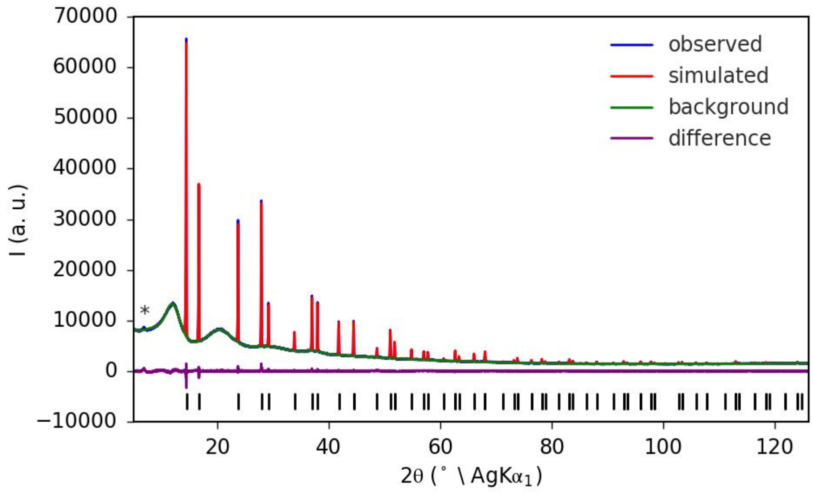

Figure S6: Fit (red) from Rietveld refinement of FCC metallic iridium structure (space group Fm-3m) and the XRPD pattern measured from the IrO_x sample (blue) are shown. The difference is shown offset below (purple), and the reflection positions are indicated below by vertical black lines. A significant amount of scattered intensities are concentrated in the diffuse scattering component, highlighted by the background function (green), which represents the amorphous IrOx component. A small peak unindexed by FCC iridium is also marked (*) at approximately 6.9°.

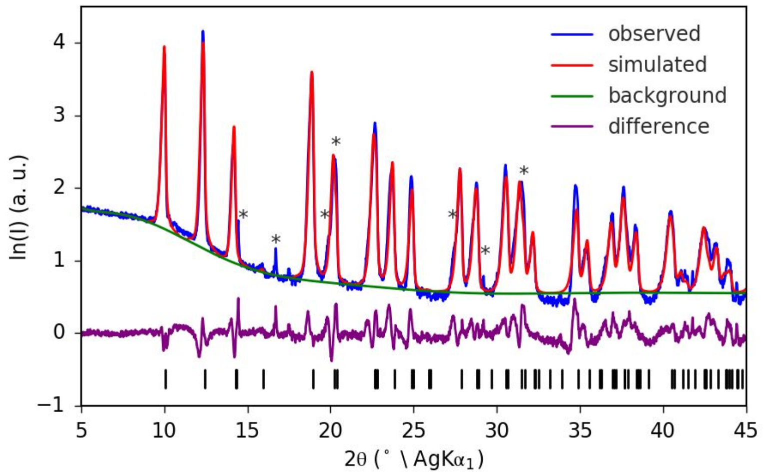

Figure S7: Fit (red) from Rietveld refinement of Rutile-type IrO2 structure (space group P42/mnm) and the XRPD pattern measured from the IrO2 sample (blue) are shown on a logarithmic scaling. The background function (green), difference (purple), and reflection positions (black vertical lines) are also shown. The fit is poor, primarily due to peaks not indexed by the structure (*), and some differences in relative peak intensity not described by the structure.

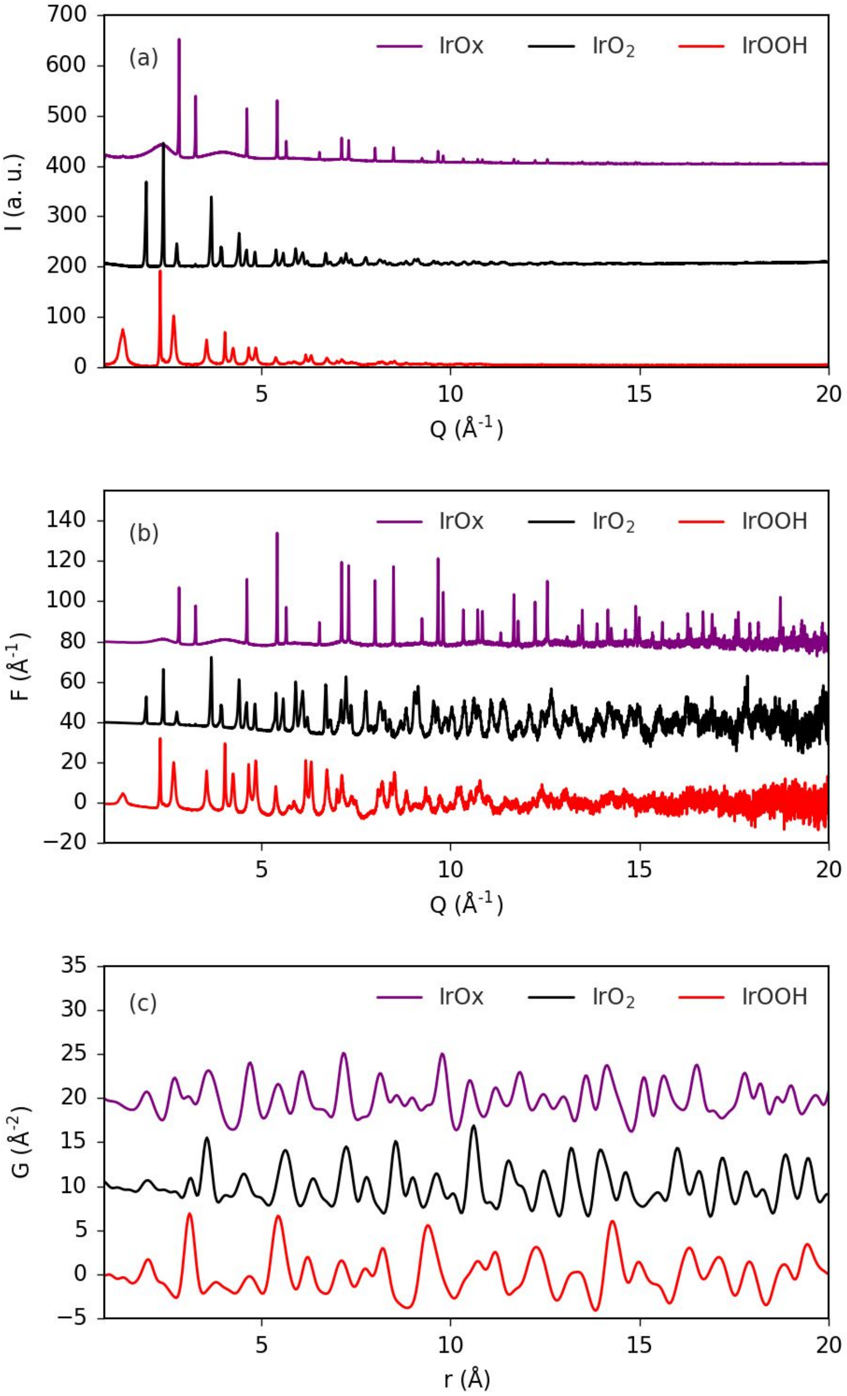

Figure S8: (a) Background subtracted total scattering intensities, (b) reduced total scattering structure functions, and (c) PDFs for the three samples are shown.

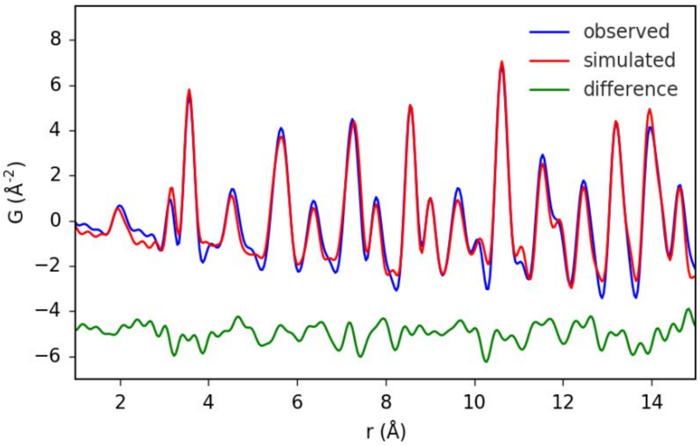

Figure S9: Fit (red) from real-space refinement of Rutile-type IrO_2 structure (space group P42/mnm) and the PDF measured from the IrO_2 sample (blue) are shown, $R_w = 0.212$.

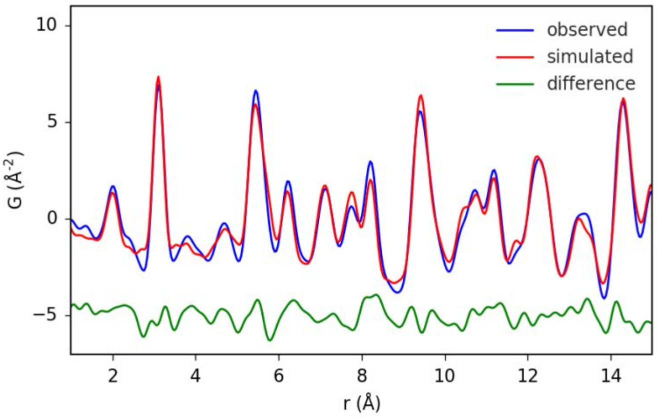

Figure S10*:* Fit (red) from real-space refinement of Heterogenite IrOOH structure (space group P63/mmc) and the PDF measured from the IrOOH sample (blue) are shown, $R_w = 0.213$.

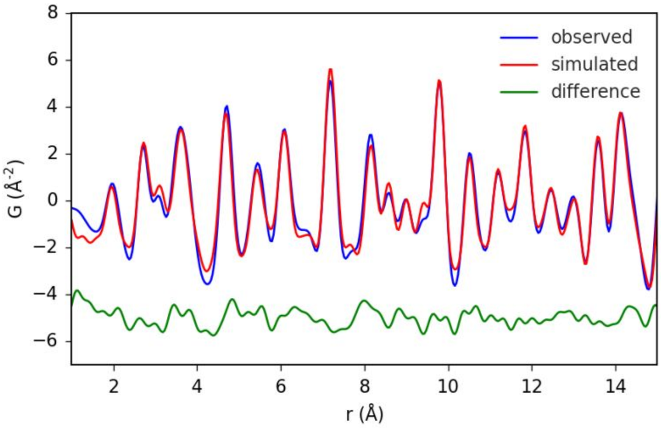

Figure S11: Fit (red) from multiphase real-space refinement of metallic iridium, Hollandite and Rutile-type IrO_2, and the PDF measured from the IrO_x sample (blue) are shown, $R_w = 0.200$.

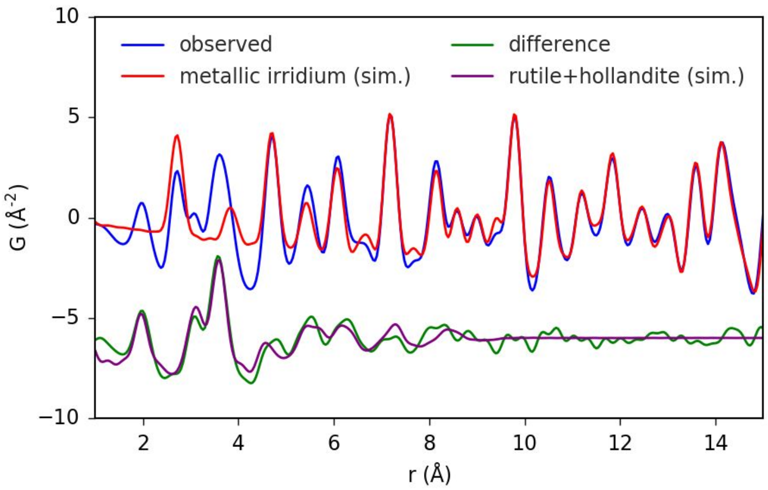

Figure S12: Contribution from metallic iridium (red) is compared to the measured PDF for IrO_x (blue). The difference (green) indicates the local structuring of the amorphous component and is compared to the separated Hollandite + Rutile-type phase (purple), both resulting from the multiphase refinement shown in Figure S9.

Table S1: Approximate atom-pair distances for Ir-O and Ir-Ir pairs extracted by fitting a Gaussian to the peak positions.

	IrOOH	IrO_2	IrOx (amorphous)	IrOx (metallic Ir)
Ir-O / Å	2.009	1.992	1.988	-
Ir-Ir / Å	3.110	3.133, 3.569	3.096, 3.613	2.697

Temperature programmed reduction (TPR)

A homebuilt setup was used for TPR experiments using quartz reactor tubes inside a tube furnace and gas dosing valves with a low dead volume. The sample powder was weighed on a high-precision scale (target of 45 mg), filled in a flow-through reactor tube made of quartz glass, and held in place by quartz wool. Prior to experiments, the samples were dried in a flow of pure Ar at room temperature for at least one hour. Chemical reduction of the powdered materials was done in a constant flow (100 mL/min) of 4.95 % H_2 in Ar. For the reduction itself, we used a flow of 100 mL/min, which results in a contact time of 65 s at room temperature. The switch between reactive and inert gas atmosphere was realized with valves optimized for a low dead-volume. Hydrogen consumption was measured by an X-Stream thermal conductivity detector by Emerson. The temperature program consisted of a heating ramp (6 K/min) to 450 ˚C, a dwelling step of 10 mins at the highest temperature, and a cool-down. Oxidation states were referenced to the total hydrogen consumption during the reduction of CuO with the same molar loading (~17 mg of CuO). The maximum temperature for the reduction of CuO was 700 ˚C.

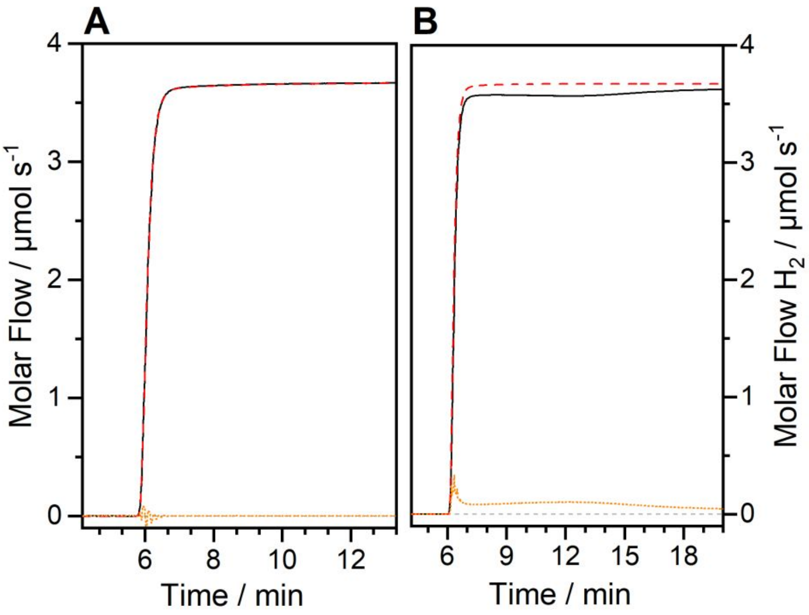

Figure S13: Introduction of 4.95 % H_2 in Ar to the reactor before (solid line) and after (dashed line) complete reduction and their difference (dotted line) for a) IrOOH and b) AA-IrOx.

The oxidation state in IrOOH was calculated based on the amount of hydrogen that was consumed in the reduction process. The amount of hydrogen was obtained by the integration of the absolute difference between the hydrogen concentration measured with the fresh sample and the same sample after reduction. In this way, we also capture reductive processes at room temperature (see **Figure S13**), which was especially relevant for the material AA-IrOx. The total amount of hydrogen consumed was then used for the calculation of the equivalent weight of O-H. Since the latter depends on the speciation, i.e. oxo-groups (need 1 H_2 to reduce to H_2O) or hydroxo-groups (need ½ H_2 to reduce to H_2O), the result was calculated for a range of possible oxo-hydroxo ratios. In the case of IrOOH, we used the general formula $IrO_{2-2x}(OH)_{2x}$. From a given mol of consumed hydrogen we calculated the weight of $O_{2-2x}(OH)_{2x}$ that could be reduced with it, for a range of x-values in steps of Δx=0.05. By subtracting this O-H-related weight from the reactor loading (45.2 mg of powder) we get the weight of reduced iridium and can thereby calculate the iridium oxidation state by dividing twice the amount of consumed H_2 by the amount of iridium. There is only one solution x for this model, at which the calculated oxidation state is equal to the formal oxidation state (**Figure S14**). The intersection is at x=0.23 (linear interpolation), which is close to the stoichiometry $IrO_{1.5}(OH)_{0.5}$. This means that, depending on the level of exposure to air, every second hydrogen could be missing in IrOOH. The obtained value might underestimate the oxidation state of iridium because crystal water was not accounted for.

It is worth noting that the reduction process of AA-IrOx occurs at lower temperatures compared to IrOOH and it is exothermic (**Figure S15**).

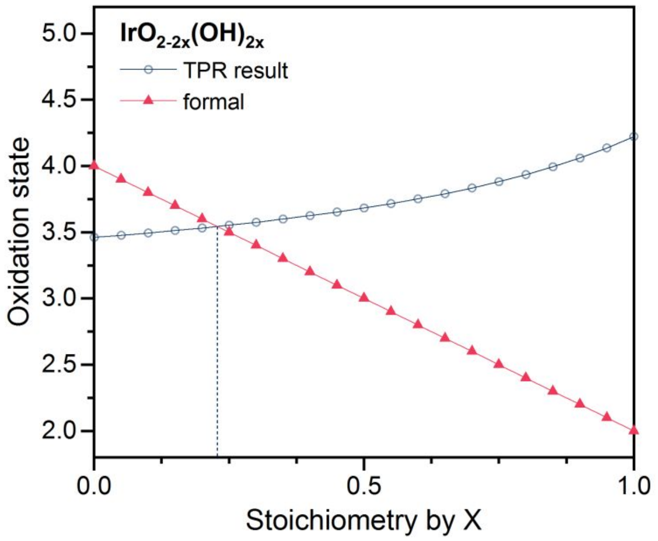

Figure S14: Chemical oxidation state of $IrO_{2-2x}(OH)_{2x}$, formally and solutions for x where the reduced weight is equal to the reactor loading.

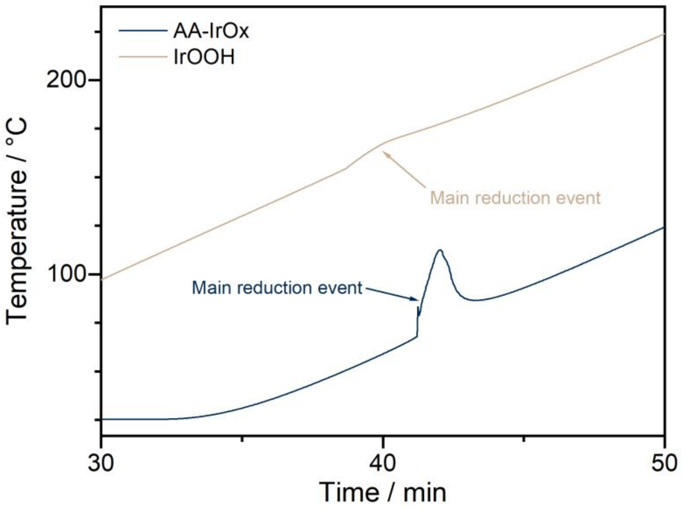

Figure S15: Temperature reading close to powder fill for IrOOH and AA-IrOx; the time-scale is relative with arbitrary zero.

UV-Vis spectroscopy

The UV-Vis absorbtance of IrOOH powder was measured in a FLS 980 photoluminescence spectrometer by Edinborough Instruments equipped with a Xe lamp as source. The IrOOH powder was dispersed in water and exfoliated IrOOH nanosheets were dispersed in ethanol. Absorptance was measured in a synchronous scan and a monochromator allowed selective detection of the incident wavelength, excluding photoluminescence. The result is given in **Figure S16**. The absorptance spectrum does not show the characteristic drop expected for gapped materials. Instead, only a dip centered at ~2.5 eV is detected.

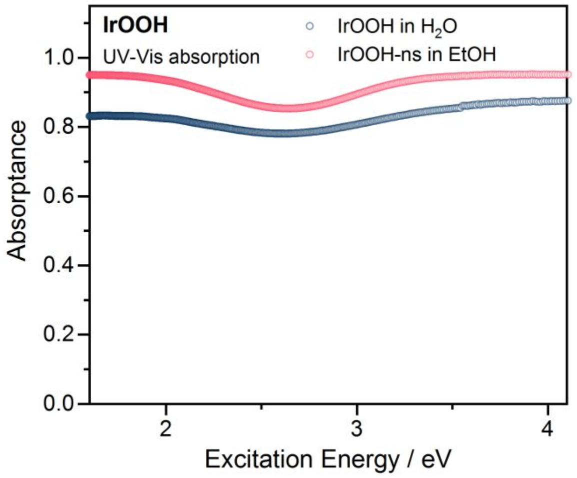

Figure S16: Absorbtance of IrOOH powder dispersed in water, excluding photoluminescence.

Diffuse reflectance was recorded on a Cary 5000 spectrometer from Agilent using an in-situ gas phase holder and a Praying Mantis cell. The materials $BaSO_4$ and Spectralon were used as white standards (see **Figure S17**). A flat surface of powdered sample was used to obtain the reflectance of the samples relative to the white standard. We used the Kubelka Munk function F(R) to calculate the absorption coefficient. The band gap was estimated by the root of a linear fit, as suggested by Tauc et al. for a direct and allowed bandgap.[16] For both white standards we found a bandgap of 1.9 eV (**Figure S18**).

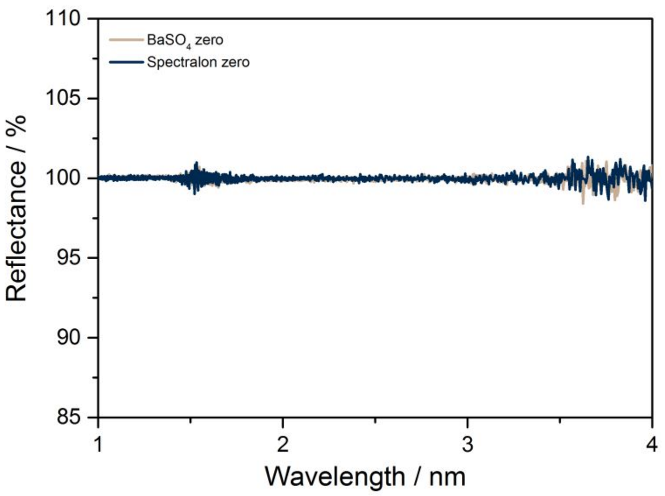

Figure S17: Reflectance of reference materials relative to reference measurement.

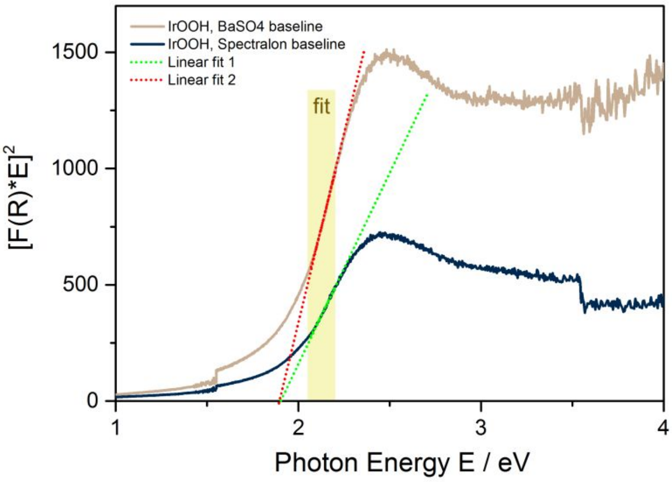

Figure S18: Tauc-plot for a direct allowed bandgap transition (squared product of the absorbance and the excitation energy) for IrOOH normalized by two different white standards; fitting of linear functions (dotted lines) in the yellow region.

Processing of X-ray spectroscopy

X-ray spectroscopy was recorded at the BESSY II synchrotron facility in Berlin at either the ISISS or the UE56-2 PGM1 beamline. Data acquisition was performed with a differentially pumped analyzer by SPECS (Berlin) using the Prodigy recording software, also by SPECS. Absorption spectroscopy was processed with a homemade Python script. The script calibrates the excitation energy by using gas phase signals, i.e. water or CO, or features in the mirror current. In the case of water vapor, the 3p Rydberg state was set to 537.25 eV following the recommendation in reference [17]; in the case of CO, the 2p π* signal was set to 534.1 eV following reference [18]. The script furthermore corrects the recorded intensity for synchrotron ring current and beamline flux. The latter was achieved by recording flux curves on an oxygen-free gold foil at elevated temperature. The flux curves for the operando measurements were recorded in the respective water vapor pressure, i.e. 0.25 mbar. Constant values were used for background subtraction. X-ray photoelectron spectroscopy (XPS) was processed and fitted using the CasaXPS software. The excitation energy was calibrated with tables created in the same week using a gold reference or, in the case of thin samples such as IrOOH-ns, with the difference in binding energy (BE) between XP spectra excited with first and second order contributions of the bending magnet of the ISISS beamline. The error of the energy calibration is smaller than 0.15 eV for the tables and smaller than 0.1 eV for the second order alignment. XP spectra were used as recorded, normalized from 0 to 1, or fitted using a Shirley-type background. The information about the background is provided in each case. Differences between two spectra were calculated by linear extrapolation of one dataset to the binding-energy scale of the other.

O K-edge integration

Oxygen states in iridates are hybridized with the metal center and therefore show a t_{2g}-like and e_g-like contribution. Assuming that the hybridization is similar for different oxidation states, the integrated absorption signal of the O K-edge can give insight into the occupation of metal-centered orbitals. However, because of the stated assumption and the unknown shape of the background below the O K-edge signal, this method needs verification. We therefore integrated the O K-edge absorption signal (partial electron yield) of rutile type IrO_2 from the pre-edge to the first minimum at about 531 eV and to the second minimum at about 536 eV (see **Figure S19**). Since we would expect 4 electron holes contributing to the intensity of the e_g-like orbitals, a quarter of the difference between the two integrations is the intensity representative of one electron hole in the e_g. The analysis gives 5.8 for a quarter of the difference, which is the intensity of one electron hole. The t_{2g}-contribution itself amounts to 5.8 as well, indicating one electron hole in t_{2g}-like orbitals. Since expectation and testing agree, we applied this method to IrOOH. With the same analysis, we obtain 5.14 for the quarter-difference and 3.6 for the white line intensity. This would mean that 70% have a hole in t_{2g}-like states, which is in fair agreement with the value obtained from TPR.

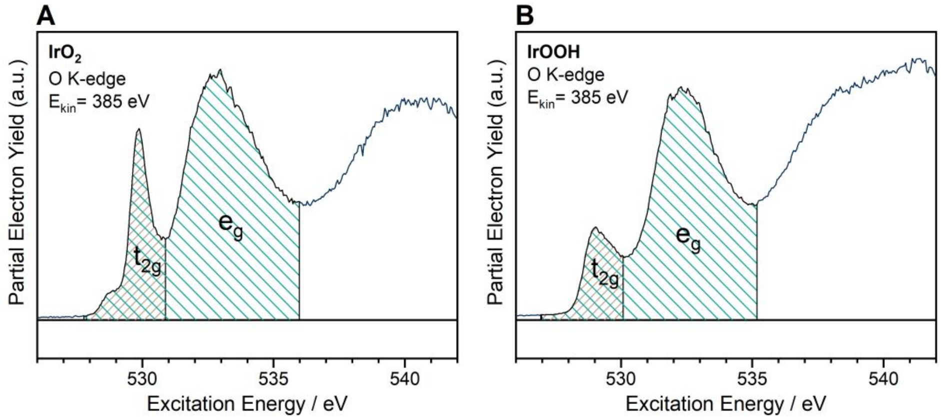

Figure S19: O K-edge integration for IrO_2 and IrOOH.

Ir L-edge integration

Iridium $L_{2,3}$-edges (**Figure S20** and **Figure S21**) were recorded at the beamline BL17C1 of the National Synchrotron Radiation Research Center (NSRRC) in Hsinchu (Taiwan). The signal was collected in fluorescence mode for the powdered samples. The spectra were corrected by an arctan background and integrated. Reference compounds $IrCl_3$, metallic iridium, rutile IrO_2, and $KnaIrO_2$ have been used for calibration and the compounds with unknown oxidation state were evaluated on a linear fit of the data (**Figure S22**).

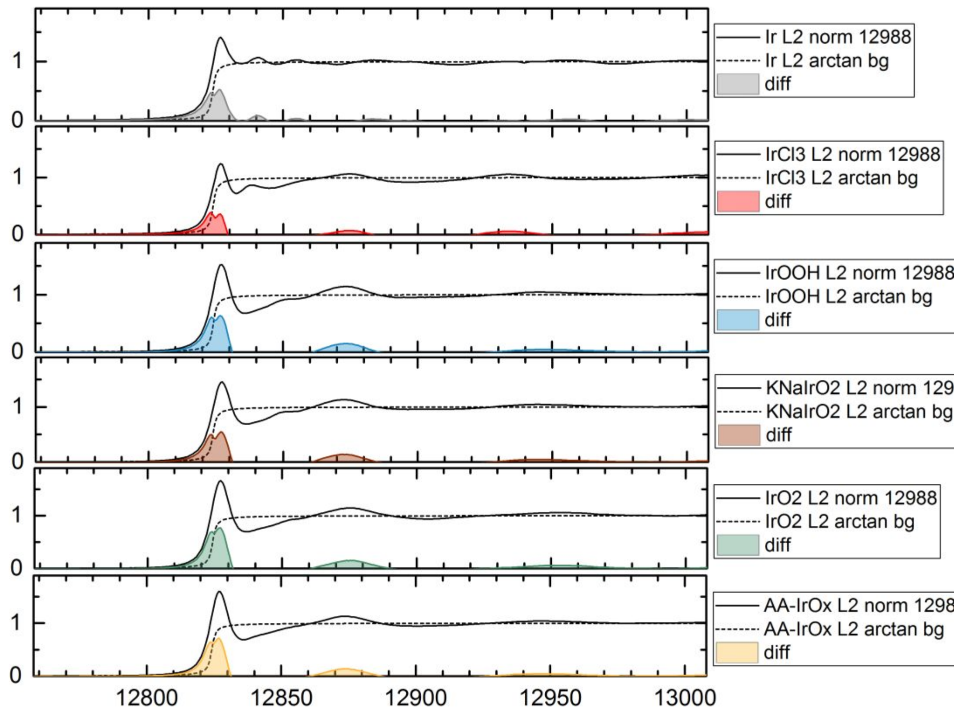

Figure S20: Ir L_2 edge absorption, arctan background and integrated difference.

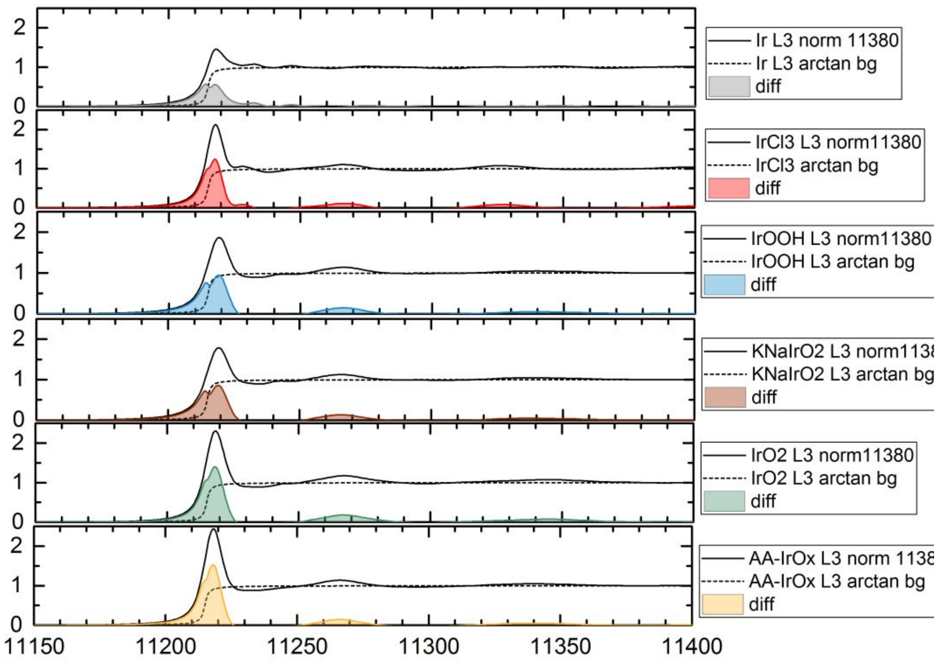

Figure S21: Ir L_3 edge absorption, arctan background and integrated difference.

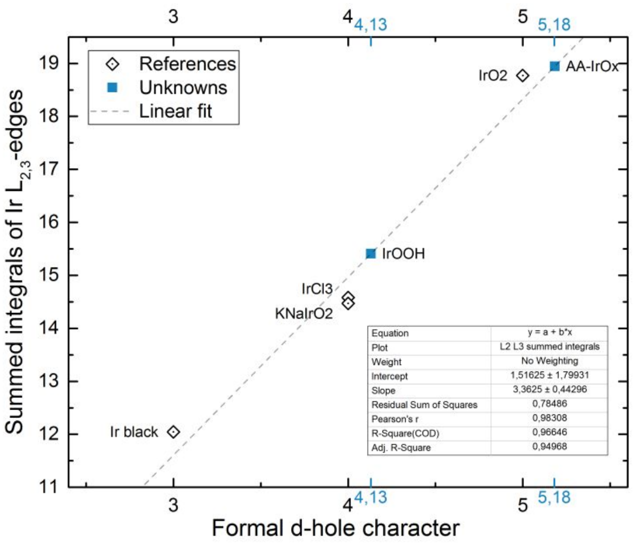

Figure S22: integrated intensity from Figure S20 and Figure S21. The calibration line was fitted to the reference materials and evaluated for the unknown compounds.

Ab initio bulk and basal plane structures

Bulk IrOOH was compared in two structures, the heterogenite-2*H* and the brucite-1*T* structure (**Figure S23**). IrO_2 in a rutile structure was used for reference purposes. The lattice parameters, the cohesive energy, and the formation energy of all bulk structures are given in **Table S2**. The calculated lattice parameters a_0 and c_0 of rutile-type IrO_2 at 0 K are 0.68 % and 0.75 % larger than the experimental values at 300 K.[19]

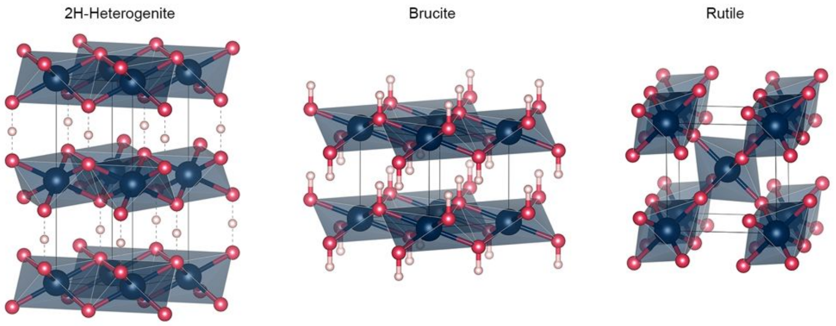

Figure S23: Bulk atomic structure of heterogenite-2H, brucite, and Rutile; dark blue, red, and light pink spheres denote Ir, O, and H atoms, respectively.

Table S2: DFT-calculated lattice parameters (a_0, c_0), cohesive energy (E^{coh}), and formation energy (E^{form}) of bulk iridium oxides.

**Structure**	**a_0** (Å)	**c_0** (Å)	**E^{coh}** (eV / atom)	**E^{form}** (eV / atom)
Heterogenite-2*H*	3.234	8.509	-4.580	-0.920
Brucite	3.537	3.762	-4.047	-0.665
Rutile	4.529	3.178	-5.292	-1.170

Bulk IrOOH was determined to adopt the heterogenite-2*H* structure by XRD (see above). The quantity and position of hydrogen in heterogenite-2*H* is, however, unknown from XRD. We tried to evaluate if hydrogen vacancies (V_H) could thermodynamically form in air by a computational experiment. Four structures were picked: the heterogenite-2*H* structure with no vacancy, one vacancy (V_H), and two vacancies (2 V_H), while the latter has been calculated in two variants with vacancies in the same plane and in different planes (see **Figure S24**). The formation energies of the vacancies with respect to the parent heterogenite-2*H* structure are given in **Table S3**. As it appears in the table, the formation of hydrogen defect is thermodynamically favorable against a reservoir of water and oxygen, so that heterogenite-2*H* is likely to have hydrogen defects when stored on air. Removal of the first and second hydrogen are equally exothermic.

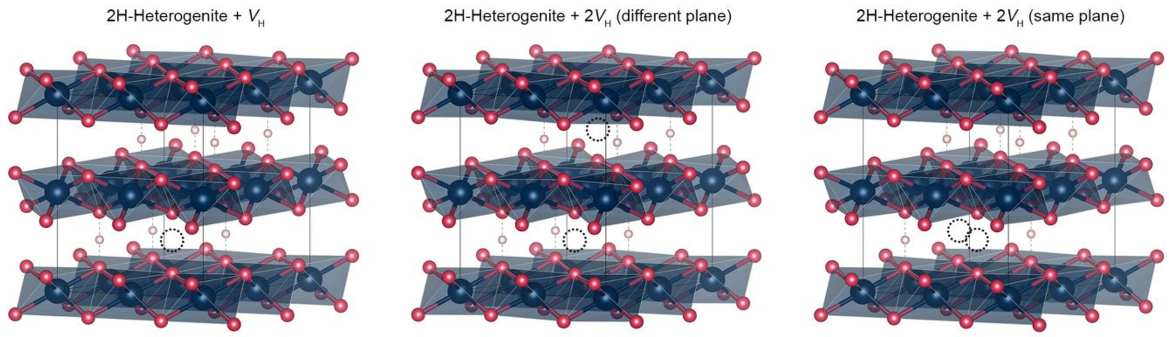

Figure S24: Side view of 2H-Heterogenite with hydrogen vacancies (V_H); dark blue, red, and light pink spheres denote Ir, O, and H atoms, respectively; the dotted circle is drawn to highlight the position of hydrogen vacancies in the unit cell.

Table S3: Hydrogen defect (V_H) formation energy in defected heterogenite-2H structures.

**Structure**	$E^f(\mu_H = \frac{1}{2}E_{H_2})$ (eV / atom)	$E^f(\mu_H = \frac{1}{2}(E_{H_2O} - \frac{1}{2}E_{O_2}))$ (eV / atom)
Heterogenite-2*H* + V_H	0.900	-0.635
Heterogenite-2*H* + 2V_H (different plane)	1.763	-1.307
Heterogenite-2*H* + 2V_H (same plane)	1.788	-1.282

It was also measured by means of polyhedral distortion indices if hydrogen defects could be responsible for structural lattice distortions. We chose the quadratic elongation ($<\lambda>$) and bond angle variance (σ^2) as distortion indices and define them by

$$<\lambda> = \frac{1}{n}\sum_{i=1}^{n}\left(\frac{l_i}{l_0}\right)^2$$

$$\sigma^2 = \frac{1}{m-1}\sum_{i=1}^{m}(\phi_i - \phi_0)^2$$

, where n is the coordination number of the polyhedral center (n = 6 in our work where Ir is in the octahedral environment); l_i is the distance between polyhedral center and i^{th} vertex atom; l_0 is the counterpart of l_i measuring the center-to-vertex distance of an ideal polyhedron having the same volume to target polyhedron; m, ϕ_i, and ϕ_0 denotes the number of bond angles in target polyhedron, i^{th} bond angle, and the ideal bond angle of the regular polyhedron. The results are presented in **Figure S25**. The lattice constants change by about 0.1 Å (see **Figure S25a**) when removing every fourth hydrogen atom from the layers, while the in-plane constant as well as the octahedral volume (see **Figure S25b**) contract, and the distance between the layers increases, while a larger scattering in the distortion of the octahera is observed (**Figure S25c-d**).

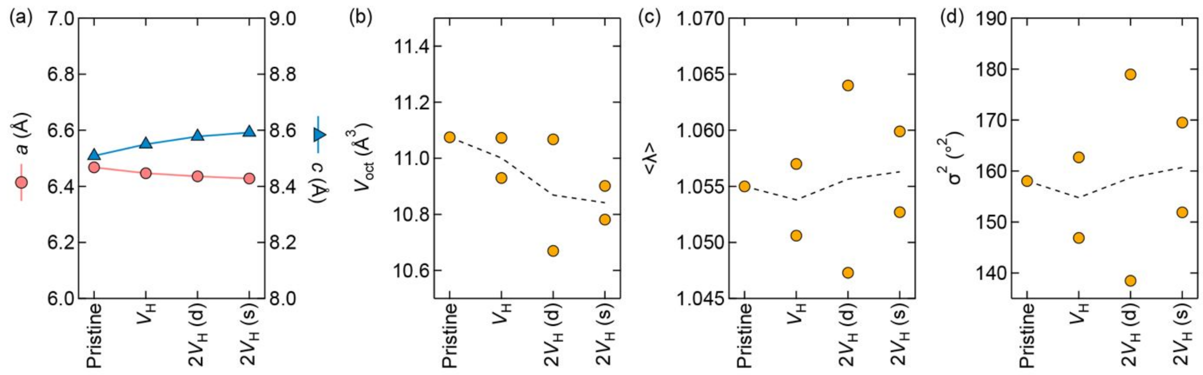

Figure S25: (a) DFT-calculated lattice parameter a and c, (b) volume of Ir-O$_6$ octahedra, (c) quadratic elongation, and (d) bond angle variance against different vacancy configurations of heterogenite-2H; dotted lines in b-d are to show the averaged value.

The density of states (DOS) and respective calculated O K-edge absorption spectra are given in **Figure S26** and **Figure S27**, respectively. In the pristine case is a clear gap of 1.3 eV. A single vacancy in one of the two layers adds states at 0 eV and an absorption white line at 529.7 eV appears. For two vacancies, the doping states are shifted to higher energies (0.2 eV) and increasingly connected to the valence band, particularly with neighboring vacancies (2 HV 2:0). The white line intensity is stronger when both layers have hydrogen defects.

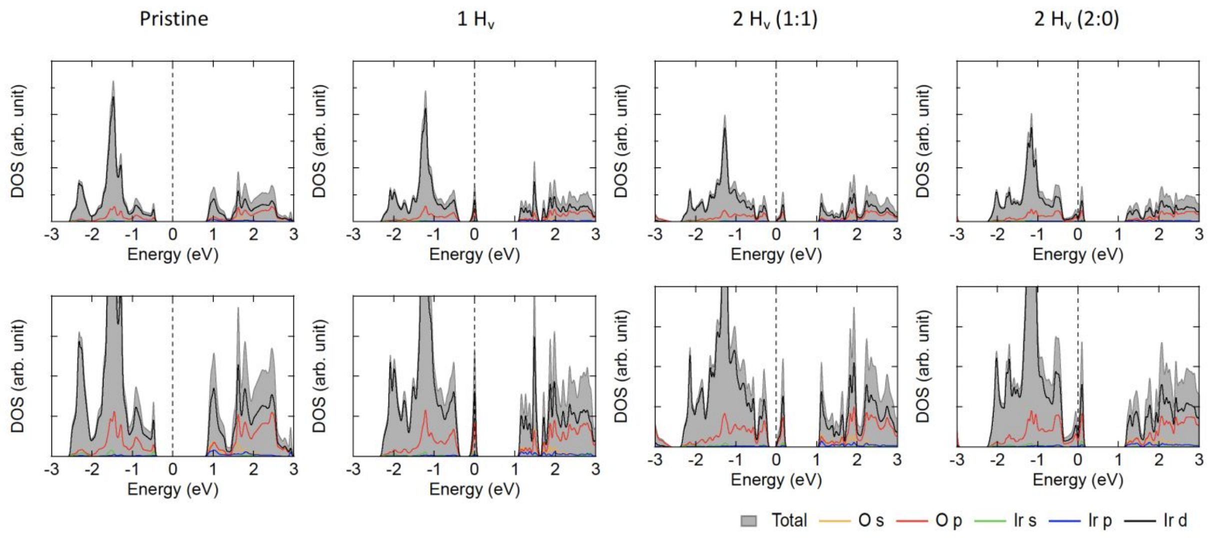

Figure S26: Density of states (DOS) of pristine bulk heterogenite-2H IrOOH and versions with defects, as shown in Figure S24.

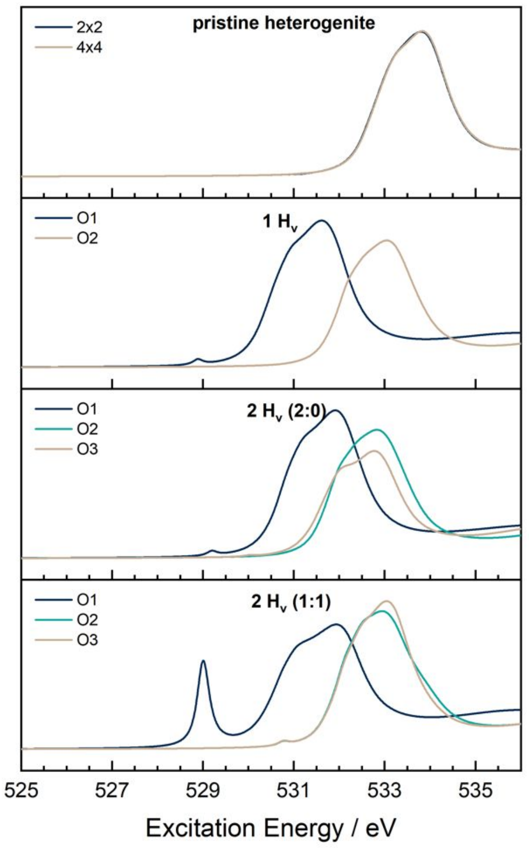

Figure S27: Calculated absorption spectroscopy of pristine bulk heterogenite-2H IrOOH and versions with defects, as shown in **Figure S24**.

The hydrogen vacancies also introduce states at and around the Fermi level (see **Figure S26**), which also leads to a absorption white line at 529 eV (see **Figure S27**). The energy of the O K-edge spectra is calibrated to the maximum white line absorption of $\mu_{3\Delta}$–O in the 2 H_v (1:1) structure, which was set to

529.0 eV (**Figure S27**). When referenced to the bulk absorption of a IrO_2 rutile sructure, we obtained values 0.73 eV larger. In the case of nanosheet edge structures, we used the same $\mu_{3\Delta}$–O white line feature of structure II in **Figure 4** of the main text as the 529.0 eV reference.

We define three different 2D IrOOH nanosheet structures, namely the $IrOOH_{asym}$, *d*-$IrOOH_{sym}$, and *p*-$IrOOH_{sym}$ (see **Figure S28**). $IrOOH_{asym}$ has asymmetrically adsorbed H atoms on one side of the 2D IrOO nanosheet, while the $IrOOH_{sym}$ structures have symmetrically adsorbed H atoms on both sides. The prefix *d*- (diagonal) and *p*- (parallel) in front of the $IrOOH_{sym}$ label denote the configuration of H atoms seen in the top view (see bottom of **Figure S28**). The lattice parameter *a*, the cohesive energy, and the formation energy of the structures are provided in **Table S4**. How the degree of protonation affects the atomic structure is measured with the octahedral distortion, as done above. A significant contraction of the octahedra (**Table S4** and **Figure S29a**) accompanied by a reduced distortion (**Figure S29b-c**) is observed with an oxidation from IrOHOH over IrOOH to IrOO. At the same average oxidation state, i.e. +3 in IrOOH, the hydrogen distribution influences the volume of the octahedra strongly, while the distortion does not change much. The distortion of the rather unstable IrOHOH structure (**Table S4**) is, however, much more distorted in a similar octahedral volume, when compared to the IrOOH structures.

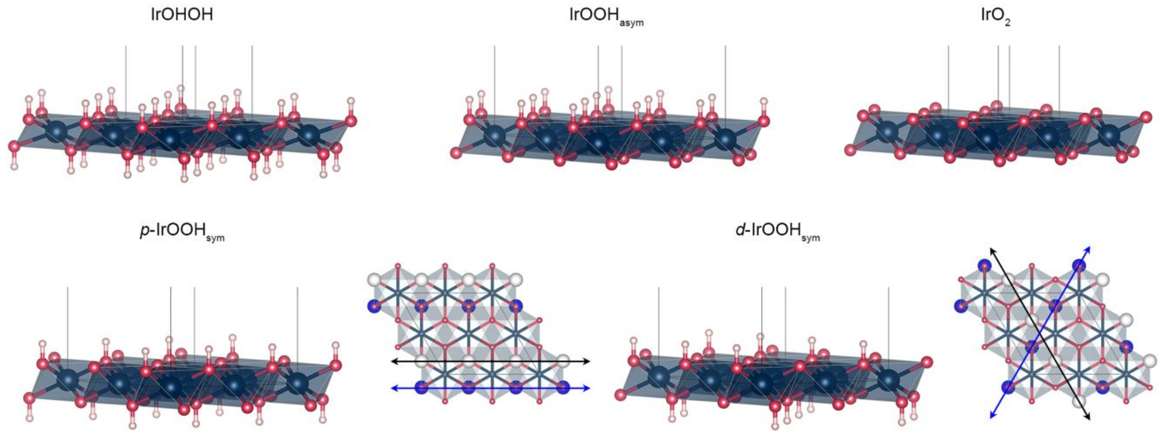

Figure S28: Atomic structure of 2D iridium oxide nanosheet structures considered; dark blue, red, and light pink spheres denote Ir, O, and H atoms, respectively; black and blue arrows plotted on the top view of p-$IrOOH_{sym}$ and d-$IrOOH_{sym}$ show the difference in the position of hydrogen atoms.

Table S4: DFT-calculated lattice parameter a, cohesive energy E_{coh}, and formation energy E_{form} of 2D iridium oxide nanosheets; the Ir–Ir distance is half the lattice parameter a.

Structure	a (Å)	Ir–O(H) (Å)	E^{coh} (eV / atom)	E^{form} (eV / atom)
p-$IrOOH_{sym}$	6.509	2.05 (2.13)	-4.412	-0.752
d-$IrOOH_{sym}$	6.493	2.07 (2.12)	-4.452	-0.792
$IrOOH_{asym}$	6.529	2.07 (2.10)	-4.382	-0.723
IrOHOH	7.144	(2.19)	-3.844	-0.462
IrOO	6.276	2.03	-5.048	-0.926

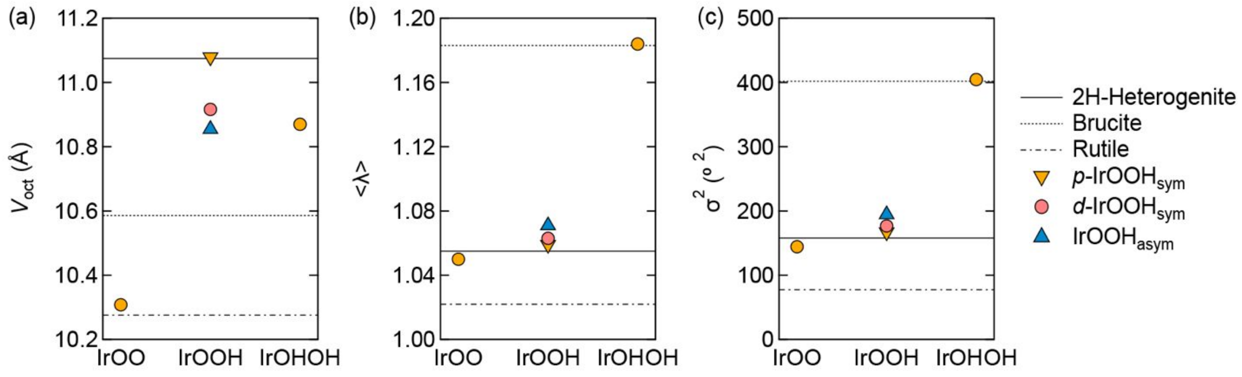

Figure S29: (a) DFT-calculated volume of Ir-O_6 octahedra, (b) quadratic elongation, and (c) bond angle variance of the considered nanosheet structures against the degree of protonation of IrOOH basal planes; the bulk structures with fixed stoichiometry are indicated as horizontal lines for comparison.

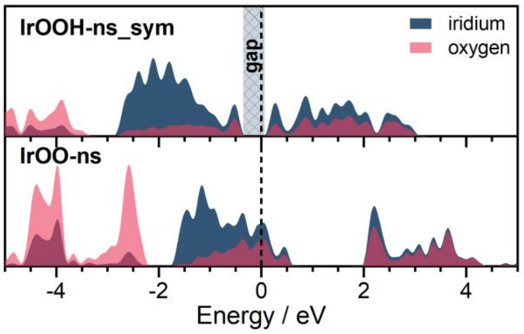

Figure S30: Density of states, or DOS, against energy relative to the Fermi Energy (dashed line) of *d*-$IrOOH_{sym}$ und IrO_2 nanosheets from the respective structures in Figure S28.

Ab initio IrOOH nanosheet edge structures

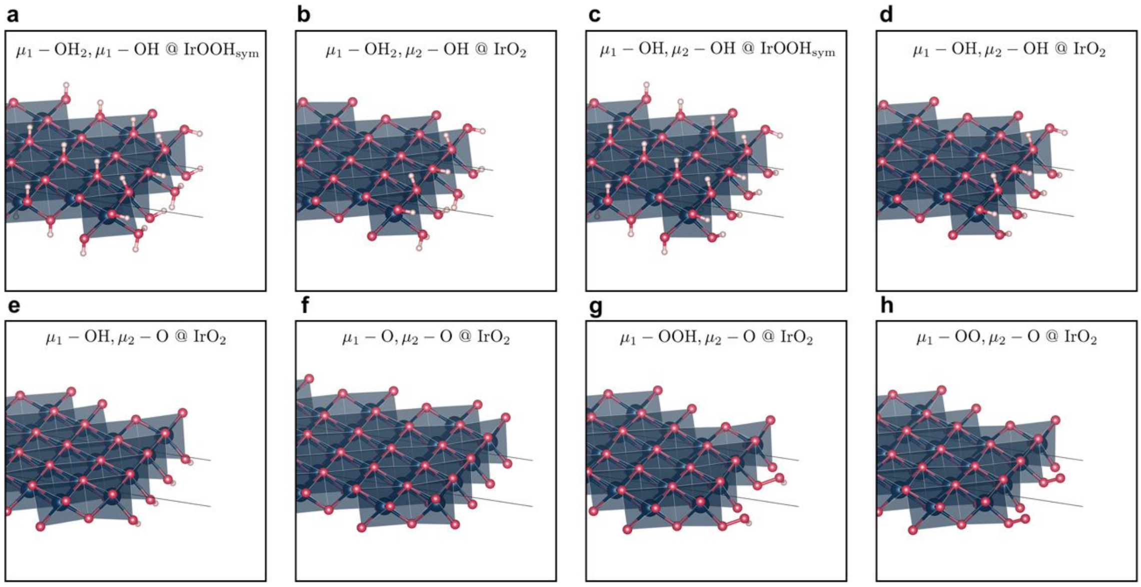

Figure S31: Atomic structure of IrOOH nanosheet edge structures with its edge exposed. Dark blue, red, and light pink spheres denote Ir, O, and H atoms, respectively.

With the aim to include all sites possibly contributing to the activity, we used edge terminated nanosheets (see **Figure S31**). Structures with an average iridium oxidation state of +2 were excluded because they are thermodynamically unfavorable (see the example of IrOHOH in **Table S4**). The stability at applied bias was caclulated by use of a computational hydrogen electrode,[20] which is shown in **Figure S32**. The respective Löwdin charges on oxygen are given in

Table S5. Examplary absorption spectra from IrOOH sheet edge structures are given in **Figure S33**.

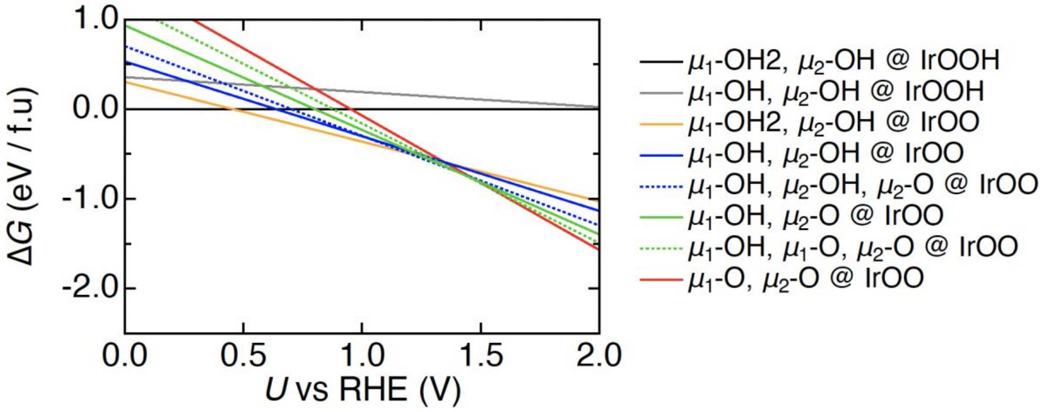

Figure S32: Ab initio Gibbs free energy of edge structures against a computational hydrogen electrode.

Table S5: Löwdin charges of the four stable structures in Figure S31, stable at <0.5V (A), 0.5-1.3V (B), 1.3-1.5V (C), >1.5V (D), with the additional structure containing μ_1–OO (E); the structure d/e stands for d with every second μ_2-OH deprotonated (see Figure S31).

Structure	μ_1-OH$_2$	μ_1-OH	μ_1-O	μ_1-OO	μ_2-OH	μ_2-O	μ_3-OH	μ_3-O
**a**	-1.24	-1.15			-0.85		-0.78	-0.29
**b**	-1.23	-1.15			-0.84			-0.29
**d**		-0.89			-0.84			-0.30
**e**			-0.16			-0.28		-0.28

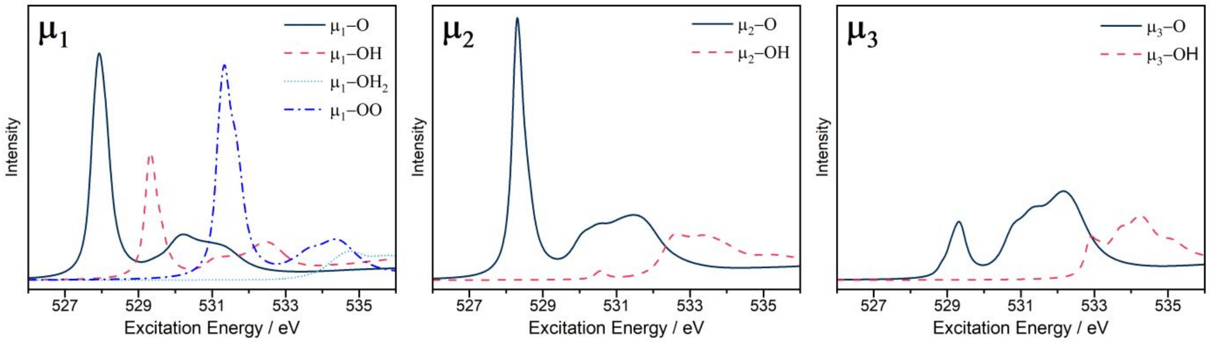

Figure S33: Representational O K-edge spectra of various oxygen species on IrOOH nanosheet edge structures.

We used structures c and d in **Figure S31** to determine the adsorption energy of hydrogen atoms on $\mu_{3\Delta}$–O and obtained 0.67 eV. This makes $\mu_{3\Delta}$–O chemically distinct from μ_3–O on IrO_2(110), for which hydrogen adsorption is 0.21 eV. Both calculations used comparable theory.

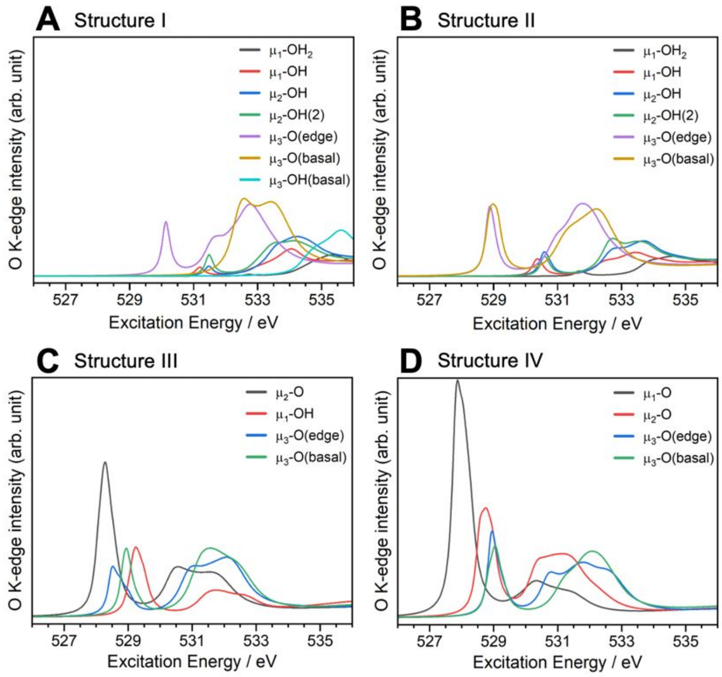

Figure S34: Calculated O K-edge spectra of unique oxygen species of the most stable structures based on the edge model; these spectra were grouped by connectivity ($\mu_{1\text{-}3}$) and averaged for Figure 4 in the main text.

The reaction barriers were calculated using a climbing image nudged elastic band method.[21] We found an oxygen release barrier with 0.7 eV to be higher than the O–O coupling with 0.4-0.5 eV. We suspect this larger barrier of release to be an error of our computational method, as observed by Ping et al.[22] First, the Perdew-Burke-Ernzerhof (GGA-PBE) type cannot capture the O_2 spin state and therefore bond energy correctly. Second, in our calculation the water molecule might not appropriately swap places with O_2. The latter is observed indirectly by comparing the top and bottom edge, for which we respectively missed the barrier or found a large activation barrier (**Figure S35**). Meta dynamic calculations in which interfacial water can swap with the surface bound O_2 and they do not show large barriers for O_2 release if solvent DOFs are retained. Third, the entropy of gas formation is not captured by our model. Fourth, to the best of our knowledge there is no experimental evidence for oxygen release to be rate limiting in the acidic OER.

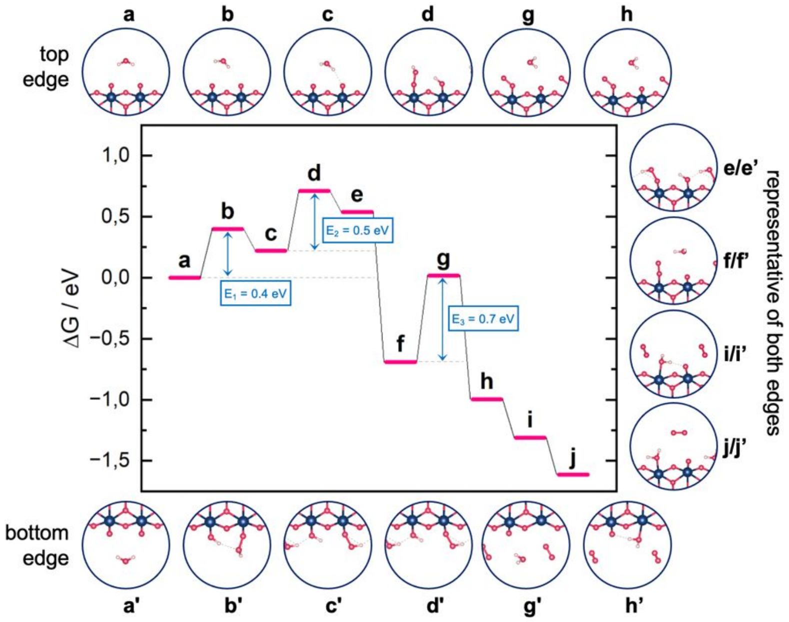

Figure S35: Calculated free energies of the reaction path for water oxidation on μ_1–O referenced to the initial state **a**, the sheet calculations have two sites, on the top and bottom edge, while the latter is marked with an apostroph; since they did not react simultaneously, the structure of the edges are shown separately for a-d and g-h, for e-f and i-j the top edge is representative for both edges (see right hand side); the activation energies for O–O coupling is 0.4 eV for the bottom edge and 0.5 eV for the top edge ($E_{1,2}$, a-e), the subsequent O_2 release (E_3, f-j) is 0.7 eV for the bottom edge with an empty μ_1–O site, and appears barrierless on the top edge, where the transition state was not observed.

BET surface area

The BET surface area of the powders was determined using an Autosorb-6-B setup by Quantachrome. The samples were dried at 423 K in vacuum and the volumetric physisorption of Kr was performed at 77 K for 11 pressures, ranging from p/p_0 = 0.05-0.3. The measurements were reproduced at least once. We determined the BET surface area of both powders. We obtained 0.56 m^2g^{-1} for IrOOH and 36.5 m^2g^{-1} for AA-IrOx.

CO oxidation in flow-through reactor

CO oxidation was carried in the same reactor as the TPR (see above). The samples were dried in pure Ar for about two hours before 250 ppm CO in Ar were introduced. We recorded concentrations of CO and CO_2 with non-dispersive infrared technique using an Advance Optima setup with an Uras 14 module from ABB.

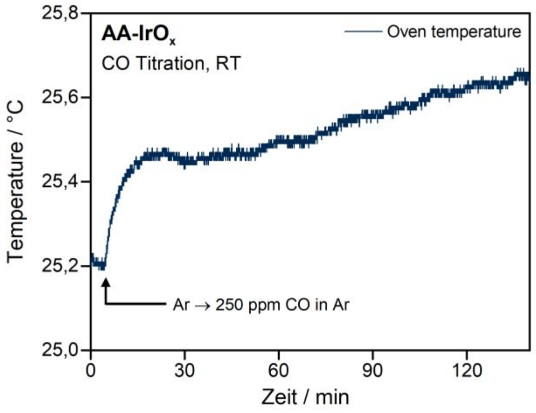

Figure S36: Oven temperature reading during CO titration with 250 ppm CO in Ar flowing at 20 mL/min.

Results of the reactor experiment using 25.2 mg of AA-IrOx are given in **Figure S37A**. Initially, 250 ppm of CO in Ar are fully converted to CO_2 by the AA-IrOx catalyst. The reaction is exothermic (see **Figure S36**) The conversion drops to 0.5 within about 30 minutes. Results for IrOOH are shown in **Figure S37C**. No conversion of CO was detected at room temperature for 25.3 mg of IrOOH powder. To assure that this result is not a surface area effect, we accounted for it using BET surface areas. IrOOH has a BET surface area 65 times smaller than AA-IrOx (see above). However, the amount of CO_2 evolving from IrOOH in the first 60 minutes on stream was only $1.1\cdot10^{-8}$ mol, which is 490 times smaller than the $5.4\cdot10^{-6}$ mol CO_2 from AA-IrOx in the same time frame. Hence, the surface area alone cannot explain the different behavior in CO oxidation.

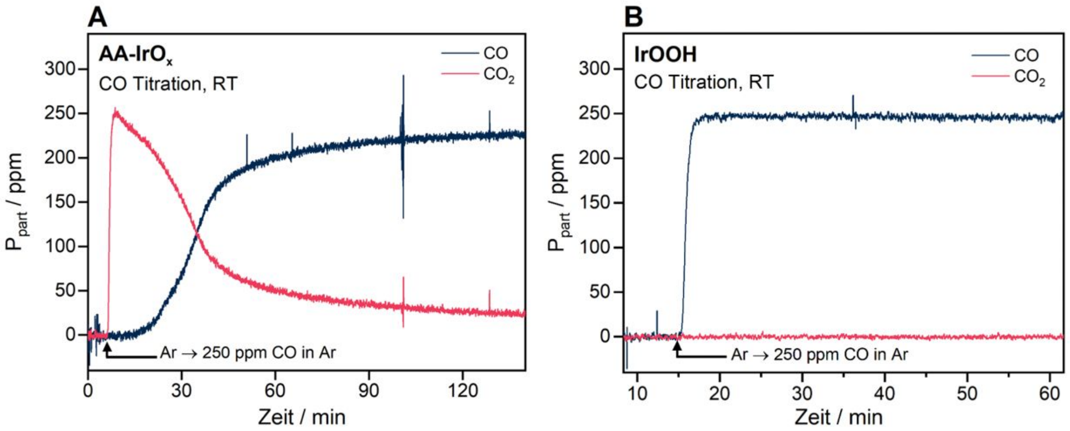

Figure S37: CO titration of the AA-IrO$_x$ and IrOOH powders; **A** AA-IrO$_x$ and **B** IrOOH powder between glass wool in 250 ppm CO in Ar flowing at 20 mL/min, an arrow on the bottom marks the introduction of CO.

In situ X-ray spectroscopy during CO oxidation at room temperature

About 20 mg of sample powder were pressed into pellets and mounted on a sapphire holder using steel plates. A thermocouple was mounted between the holey front-plate and the powder pellet for temperature monitoring. Spectroscopy was recorded at the UE56-2 PGM1 beamline at the BESSY II

synchrotron Berlin in a near ambient pressure X-ray photoelectron spectroscopy (NAP-XPS) setup. The powder pellet samples were initially kept at high vacuum. CO oxidation was carried out at a backpressure of 0.25 mbar, which is the same partial pressure as in the reactor experiment (see above). For introducing the reactive gas mixture to the measurement chamber, the sample was kept at high vacuum in a separate compartment with a smaller volume. As soon as the pressure in the main chamber stabilized, the sample was re-introduced, causing only a small change in pressure. The gas was continuously analyzed with a Prisma quadrupole mass spectrometer by Pfeiffer Vacuum GmbH (Asslar, Germany), which was connected to the main experiment chamber via a leak valve. The mass spectrometer was operated at ~$5 \cdot 10^{-7}$ mbar (**Figure S38**). The result is qualitatively the same as in the reactor experiment: AA-IrOx is active in CO oxidation at room temperature, IrOOH is not. Ten minutes after introducing AA-IrOx, it was retracted back into the load-lock, in which it regenerated. After a few minutes, it was re-introduced, which lead to increased production of CO_2 (bottom of **Figure S38A**).

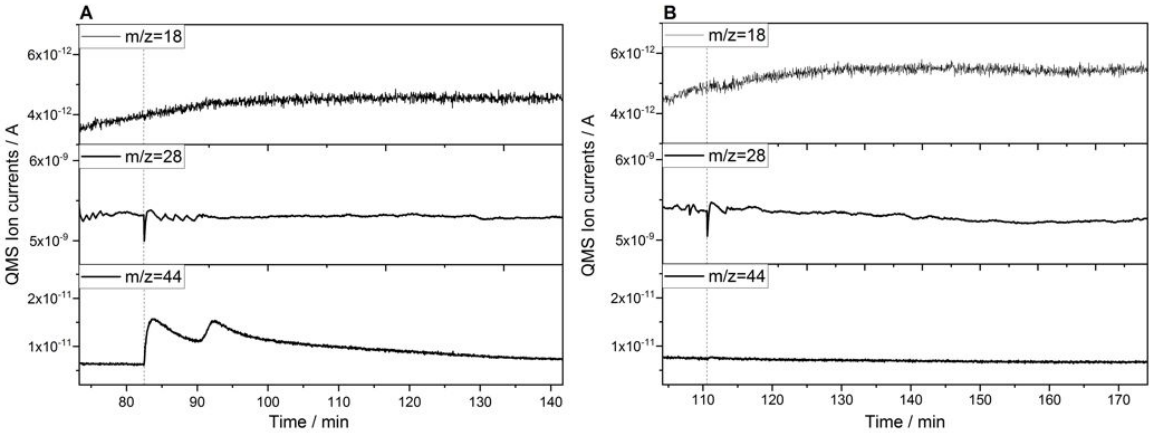

Figure S38: Online mass spectrometry of AA-IrOx in 0.25 mbar CO; dotted line indicates introduction of the sample to the measurement chamber.

In situ spectra in 0.25 mbar CO at room temperature are provided in **Figure S39**. The first spectrum in the CO atmosphere was subtracted from the consecutive spectra to show the changes more clearly. In situ X-ray absorption on the O K-edge of AA-IrOx (**Figure S39A**) indicate a strong loss of absorption at ~528.7 eV and 532 eV, which agrees well with the previous findings.[23] The loss of the electron deficient oxygen species seems to be connected to a loss of a broad feature at about 2 eV above the main lines in the Ir 4f XP spectra (**Figure S39B**). At the expense of this broad feature, a sharp doublet at 61.5 eV and 63.5 eV grows. Iridium bound to electron deficient oxygen species are reduced to metallic iridium. The same type of plot is shown for the O 1s XPS signal (**Figure S39C**). Signal intensity at 530 eV and 529 eV is reduced in CO atmosphere, while a new feature at ~533.5 eV develops. The latter is a contribution from adsorbed CO.

The same set of Ir 4f & O 1s spectra are provided for IrOOH in **Figure S39D-F** and for IrO_2 in **Figure S39G-I**, respectively. Almost no difference between the first and the second spectrum was detected. Although the changes were small, the trends for the XP spectra of IrOOH can be summarized as growing doublets at 61.5 eV and 64.5 eV at the expense of broad features at 63 eV and 66 eV, while features in the O 1s at 531.5 eV are slightly reduced. The O K-edge absorption shows losses at 529 eV and 532.5 eV, but they are weaker than for AA-IrOx. A slight surface reduction of the oxide to metallic iridium is the most likely explanation. In the case of IrO_2, a loss of a doublet at 61.8 eV and 64.8 eV is accompanied by an increasing, broader doublet at 62.5 eV and 65.5 eV. A possible explanation is the loss of the well-screened main-line, due to a reduction to an Ir^{III} species with less efficient screening. The O K-edge absorption loses some intensity characteristic for oxide features (530 eV and 532 eV), which is corroborated by the O 1s XP spectrum diminishing at 530 eV.

We can conclude from this first chemical test that AA-IrOx contains an oxygen species absorbing slightly below 529 eV, which is able to oxidize CO at room temperature. This oxygen species is electron-deficient and can be assigned to a bridging oxygens.[23] The oxygen species in IrOOH, while also appearing near 529 eV in the O K-edge, is of different chemical nature and is unable to oxidize CO at room temperature. The same is true for IrO_2.

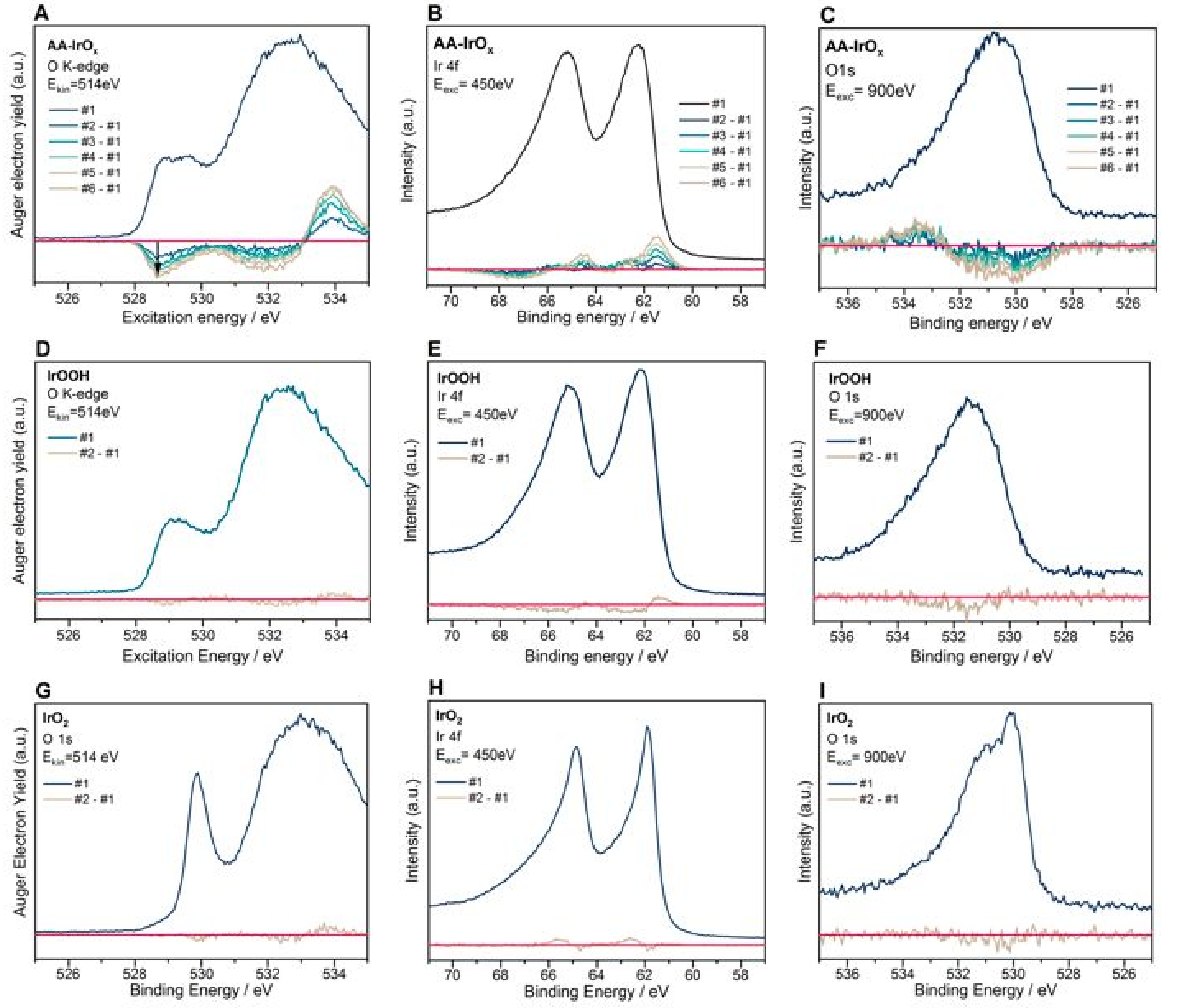

Figure S39: In situ a) d) g) O K-edge absorption, b) e) h) Ir 4f XP spectra, and c) f) i) O 1s XP spectra of a)-c) AA-IrOx, d)-f) IrOOH, and g)-i) rutile-type IrO_2 in 0.25 mbar CO at room temperature; the first spectrum in CO and the respective differences to the first are shown; spectra of the same type were recorded 8 minutes apart. **B** and **C** show the respective in situ O K-edge spectra of powder pellets in 250 mbar CO (equivalent partial pressure as 250ppm in Ar); consecutive O K-edge spectra are given as differences on the bottom; the time between consecutive measurement points is about 3 minutes for AA-IrO_x and 15 minutes for IrOOH

Electrochemical characterization

The electrochemical characterization was performed in clean glassware filled with Ar-saturated 0.1 M H_2SO_4 using a flamed Pt wire as counter electrode and a saturated $Hg/HgSO_4$ reference electrode. Catalyst inks were prepared using 5 mg ground catalyst powder in 490 µL H_2O, 490 µL EtOH, and 20 µL of a Nafion solution by Sigma Aldrich (5% Nafion in alcohols and water). Catalyst inks were sonicated for 15 mins and shortly before use. Inks older than one day were disposed. The rotating glassy carbon disk (5 mm in diameter) was polished subsequently with a 1 µm and 0.5 µm alumina suspension and cleaned before dropcasting 2 µL of the ink. The ink dried under rotation in air.

Electrochemical characterization was performed with a Biologic potentiostat before and after a stress test. The characterization consisted of impedance spectroscopy to determine the overall resistance of the system, cyclovoltammetry (CV) between 0.02 V_{RHE} and 0.45 V_{RHE} at 50 mV/s, CV series in windows of 0.05 V_{RHE} around 0.2 V_{RHE} and 1.0 V_{RHE} at 1, 2, 5, 10, 20, 50, 100, 200, and 500 mV/s, and linear sweep voltammetry (LSV) between 1.0 V_{RHE} and 1.6 V_{RHE} at 5 mV/s and CVs such as the ones shown in **FIgure S40** (equivalent to **Figure 9** in the main text). The degradation consisted of cycles starting with chrono-potentiometry at a potential at which the current density reached 2-3 mA/cm^2 in the LSV experiment followed by CVs between 0.3 V_{RHE} and 1.6 V_{RHE}. These cycles were repeated for about 3-5 hours before the final characterization was made (equivalent to initial characterization).

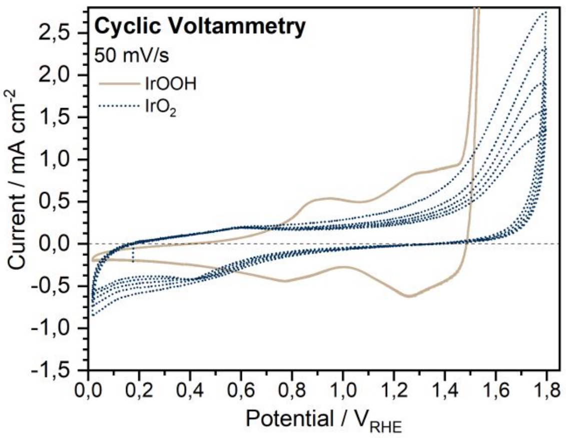

FIgure S40: Electrochemistry on a glassy carbon rotating disk: cyclic voltammetry of IrO_2 and IrOOH on a rotating glassy carbon disk electrode in Ar-saturated 0.1 M H_2SO_4, initial characterization.

The capacitance was evaluated from the CV series (see previous paragraph) using a self-written script averaging the difference in oxidative and reductive current for repetitions of CVs at a given speed (usually two repetitions). The catalyst mass was obtained from the volume that was dropcasted assuming homogeneous weight distribution in the ink. LSV measurements were then normalized by mass, geometric surface area, capacitance, or BET surface area, as given in **Table S6**. The results are given in **Figure S41**. All potentials are pH and iR corrected and are converted to the reversible hydrogen electrode (RHE).

Table S6: Catalyst mass, geometrical electrode area, initial capacitance, final capacitance, and BET surface area used for normalization of electrochemical currents of the rotating disk experiments.

	Mass *mg*	Area *cm²*	Initial Capacitance *mF/cm²*	Final Capacitance *mF/cm²*	BET m^2/g
**IrOOH**	10.2	0.196	3.8	0.9	0.56
**IrO_2**	10.0	0.196	0.5	0.3	2.1
**AA-IrO_x**	10.4	0.196	26.2	13.8	36.5

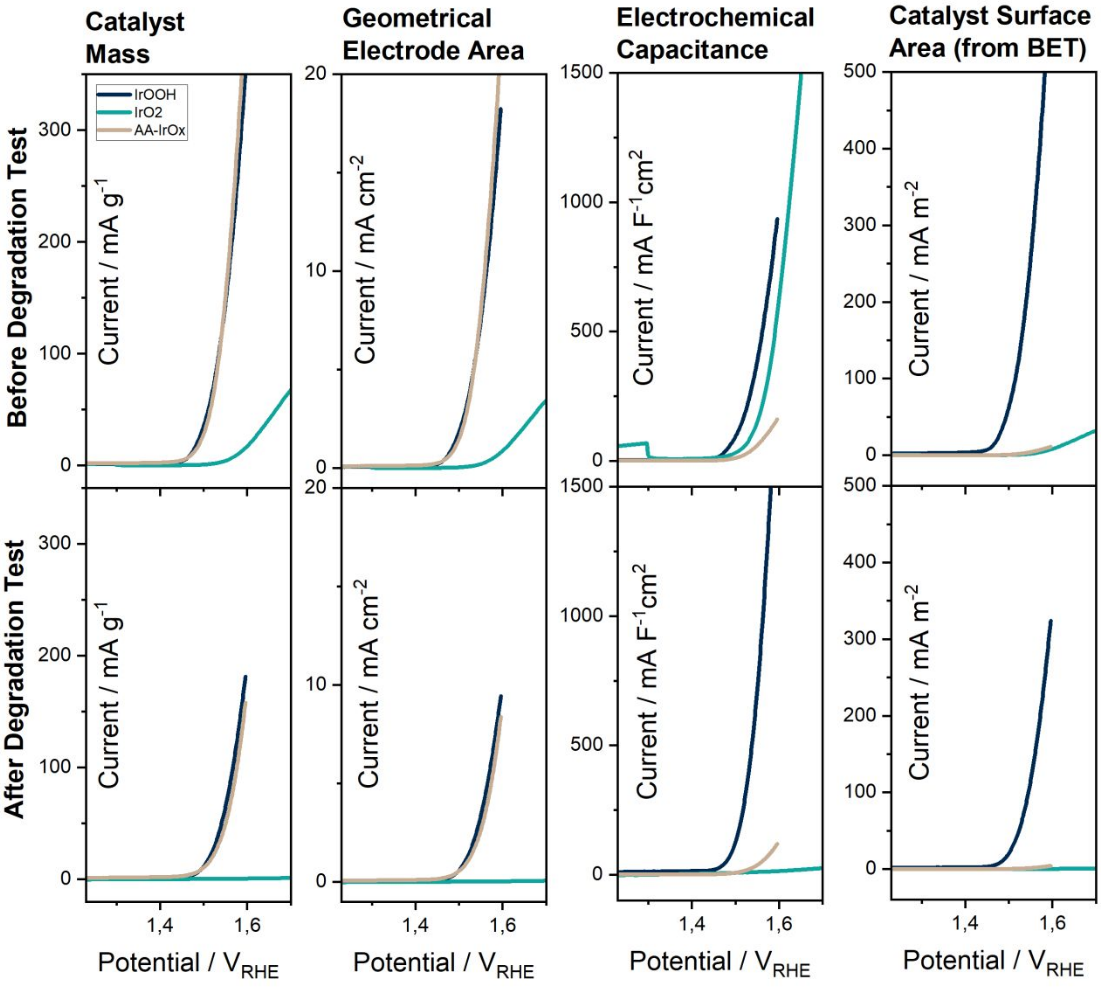

Figure S41: Linear sweep voltammetry before (top) and after (bottom) stress test, normalized to catalyst mass, geometrical surface area, capacitance, and BET surface area.

Sample preparation for ex situ TEM and operando X-ray spectroscopy

The in-situ samples were prepared from the proton exchange membrane FAD by Fumatech (Bietigheim-Bissingen). Circular disks (11 mm in diameter) were soaked for several days in 0.5 M Na_2SO_4 before transfer of graphene. Single and bilayers of graphene (SLG and BLG) were received from Graphenea (San Sebastian) on copper foil. Copper was etched in aqueous solution of $(NH_4)_2S_2O_8$ (40 g/L) overnight. After exchange of the etching solution to pure water, the swimming layer of graphene was transferred onto the substrate of choice (see **Figure S42**). Two substrates were used: gold grids from Plano

(Marburg) with circular holes of 1000 μm (Quantifoil grids R1.2/1.3 Au 200) and FAD membrane disks. IrOOH nanosheets (see above for exfoliation procedure) were deposited on SLG@Cu before etching of Cu and transfer onto the substrate. Powder samples, by contrast, were deposited directly onto FAD in form of a catalyst ink consisting of pure water and dispersed powder. In a consecutive step BLG was transferred on top of the catalyst layer.

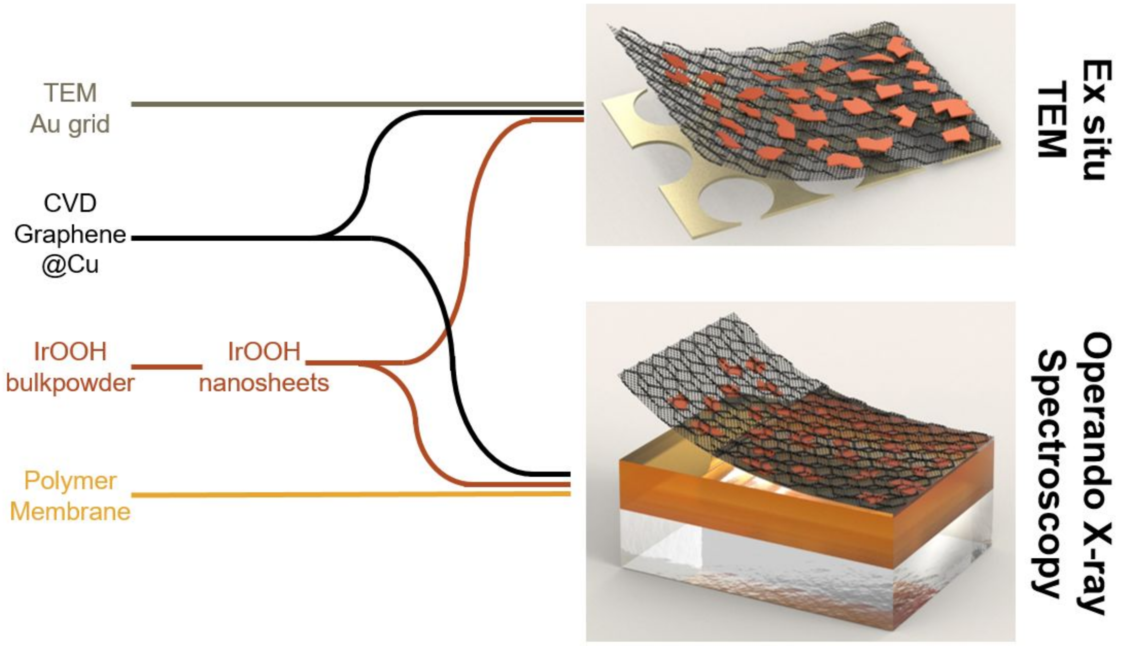

Figure S42: Schematic of the sample preparation using graphene and IrOOH nanosheets for ex situ TEM and operando X-ray spectroscopy under wet conditions.

Ex situ TEM

Transmission electron microscopy (TEM) measurements were conducted using a ThermoFisher Scientific Talos F200X, operated at 200 kV. The instrument was equipped with a ThermoFisher Scientific Ceta 16M camera and a Schottky source.

Although the crystallites are partly stacked and overlapping, the single sheets on free-standing graphene can still be seen clearly. Selected area electron diffraction (SAED, **Figure S44D**, which is reprinted from **Figure 2** in the main text for convencience) of the nanosheets as deposited shows that the crystal structure of the separate sheets is preserved in the wet chemical transfer method. The SAEDs, recorded from a single nanosheet, show a single-crystalline pattern both before (**Figure S44D**) and after (**Figure S44E**) the electrochemical treatment. Upon longer exposure with the electron beam, the SAEDs degrade to ring-like patterns, providing an excellent measure to avoid beam damage for the final SAEDs published. The nanosheets degrade to intergrown particles of a few nanometers (**Figure S43**).

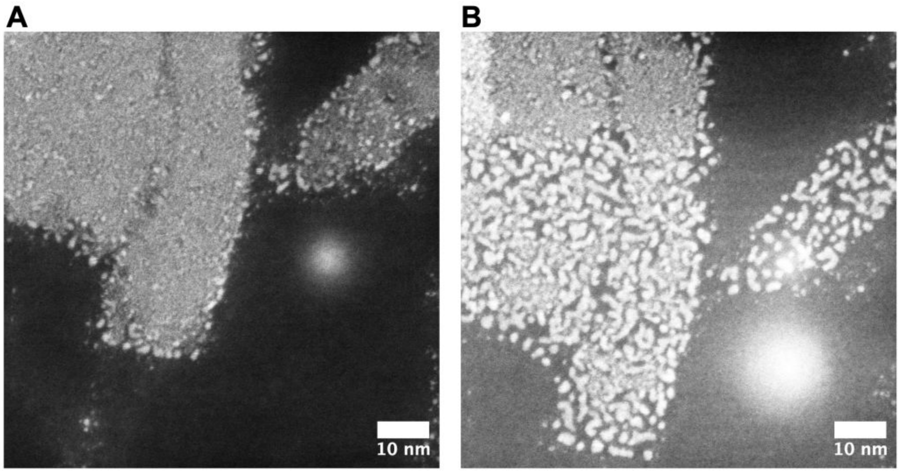

Figure S43: High-angle annular dark-field micrographs of two sheets at **A** half a minute and **B** 2 minutes exposure to the electron beam.

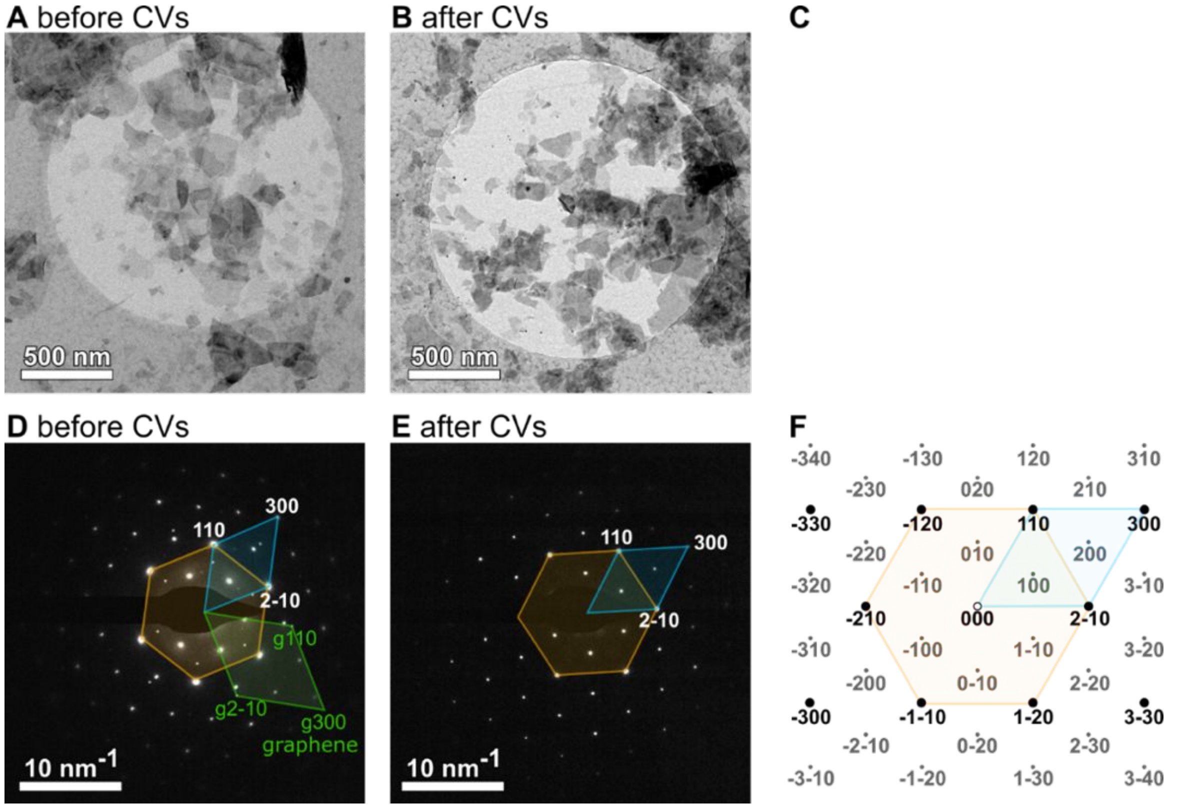

Figure S44: a)-b) Bright field micrographs and c)-d) electron diffractograms from a transmission electron microscope before and after 50 cyclovoltagrams (CVs) between 0.35 and 1.65 V_{RHE} of Au/SLG/IrOOH-ns/0.1MH_2SO_4; f) calculated map of reflection spots for the diffractograms; the polygons in d)-f) correspond to conventional (orange), primitive (blue) reciprocal unit cells of IrOOH and the primitive cell of graphene.

The recorded diffractograms agree well with the calculated [001] zone axis pattern (computed within the kinematic theory for the 2*H*-heterogenite structure, space group no 196, $P6_3/mmc$ using CrysTBox)[24] displayed in **Figure S44F**, both with respect to the lattice spacings (see **Table S7**) as well as in the relative

intensities of the spots. The latter can be explained by the thin nature of these nanosheets, rendering dynamic scattering events negligible. For easier comparison, polygons were drawn into the diffraction patterns (experimental and calculated) to represent the conventional (orange) and primitive (blue) reciprocal unit cells. In the diffraction pattern before electrochemistry, an additional lattice stemming from graphene is visible (the reciprocal unit cell is marked in green). This was used as an internal standard for the calibration of diffraction patterns.

Table S7: Measured lattice spacings from selected area electron diffraction patterns compared to calculated ones obtained from the crystallographic data.

(a) Before EC (Figure S44D)

d_{exp} / nm	d_{ref} / nm	Phase	*hkl*
0.273	0.2681 *	IrOOH	100
0.214	0.2134 [25]	Graphene	100
0.158	0.1548 *	IrOOH	110 | 2-10
0.136	0.1341 *	IrOOH	200
0.123	0.1232 [25]	Graphene	110 | 2-10
0.091	0.0894 *	IrOOH	300

* based on the XRD results obtained in the present work

(b) After EC (Figure S44E)

d_{exp} / nm	d_{ref} / nm	Phase	*hkl*
0.267	0.2681 *	IrOOH	100
0.213	0.2134 [25]	Graphene	100
0.156	0.1548 *	IrOOH	110 | 2-10
0.134	0.1341 *	IrOOH	200
0.123	0.1232 [25]	Graphene	110 | 2-10

* based on the XRD results obtained in the present work

The extracted lattice spacings are given in **Table S7** in the supporting information, which agree with literature values down to about 1%. From the {100} spots in the pattern, the unit cell size *a* (corresponding to the in-plane Ir–Ir distance) can be determined to be 0.315 nm, which is very close to the obtained XRD values. Due to the fact that the crystal is viewed in a [001] zone axis, no information

about distances along this axis is obtained and, thus, it is impossible to determine *c* or, subsequently, the Ir–O and out-of-plane Ir–Ir distances.

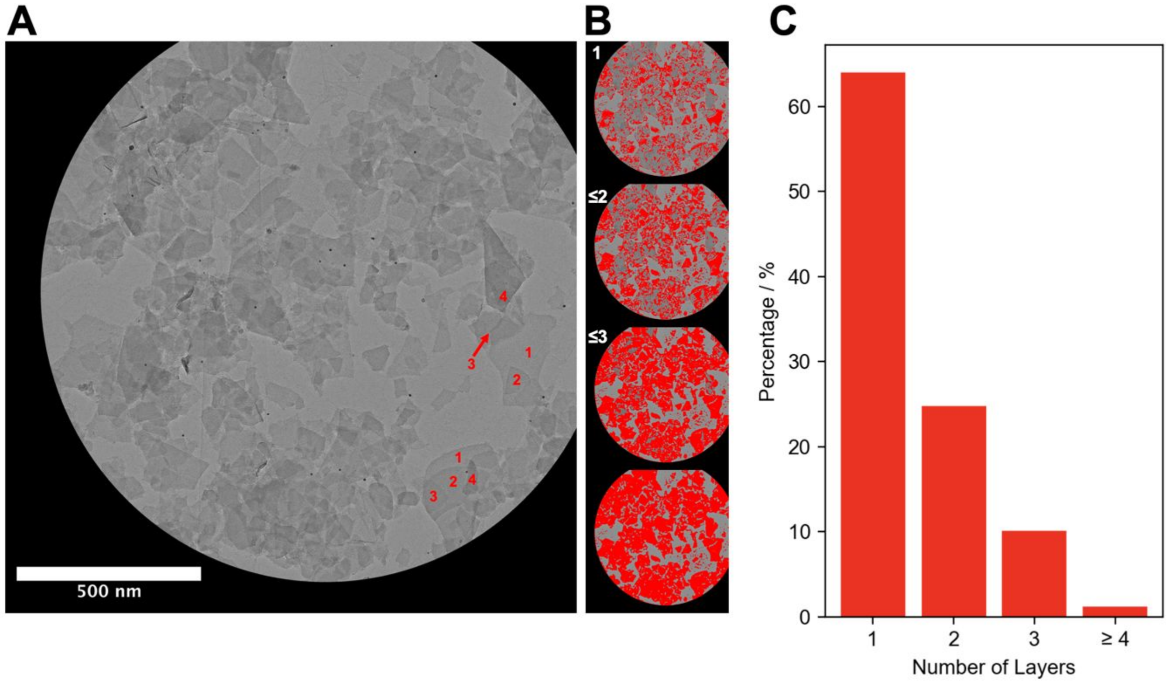

Figure S45: **A** TEM bright field micrograph of free-standing graphene and nanosheets of IrOOH; red numbers indicate areas with 1, 2, 3, or more than 4 layers; **B** copies of A, with the area for 1-4 layers successively added, judging by their contrast; **C** IrOOH coverage by layer thickness (100% on bottom image in B) as a histogram.

To quantify the stacking or folding of nanosheets, we analyzed TEM images of free-standing graphene with nanosheets (**Figure S45**) in the absence of particles. Due to the largely flat orientation of the sheets we assumed that the contrast is dominated by mass or thickness, which leads to different contrast for 1, 2, 3, and more layers (see red color for a varied thresholding in **Figure S45B**). A median smoothing of 20 pixels has been applied before thresholding to reduce effects of noise. According to the threshold analysis, about two thirds of the coverage are by monolayers (assuming the lightest contrast is corresponding to monolayers), bilayers make up a quarter of the coverage, and the rest is stacked in 4 or more layers.

Ex situ SEM

To quantify the amount of multi-layer particles after deposition of nanosheets, we used scanning electron microscopy (SEM) after dropcasting on copper-supported graphene (**Figure S46**), which was later used for in situ studies. We found the coverage of particles is around 10% by matching areas with particle morphology and bright contrast in backscattering. Upright standing aggregates that appeared with bright backscattering contrast and elongated shapes provided an insight into how many layers are stacked. These upright aggregates had between 35 and 55 nm thickness, including parallax errors and bend. Our in situ X-ray spectroscopy has a probing depth of around 3 nm, so at any of these thicknesess, the stacks

are not translucent to the measured electrons, shielding off – in a zero order approximation – half of the active centers from being measured. Accounting for these factors gives us a spectral contribution below 40% from stacked sheets.

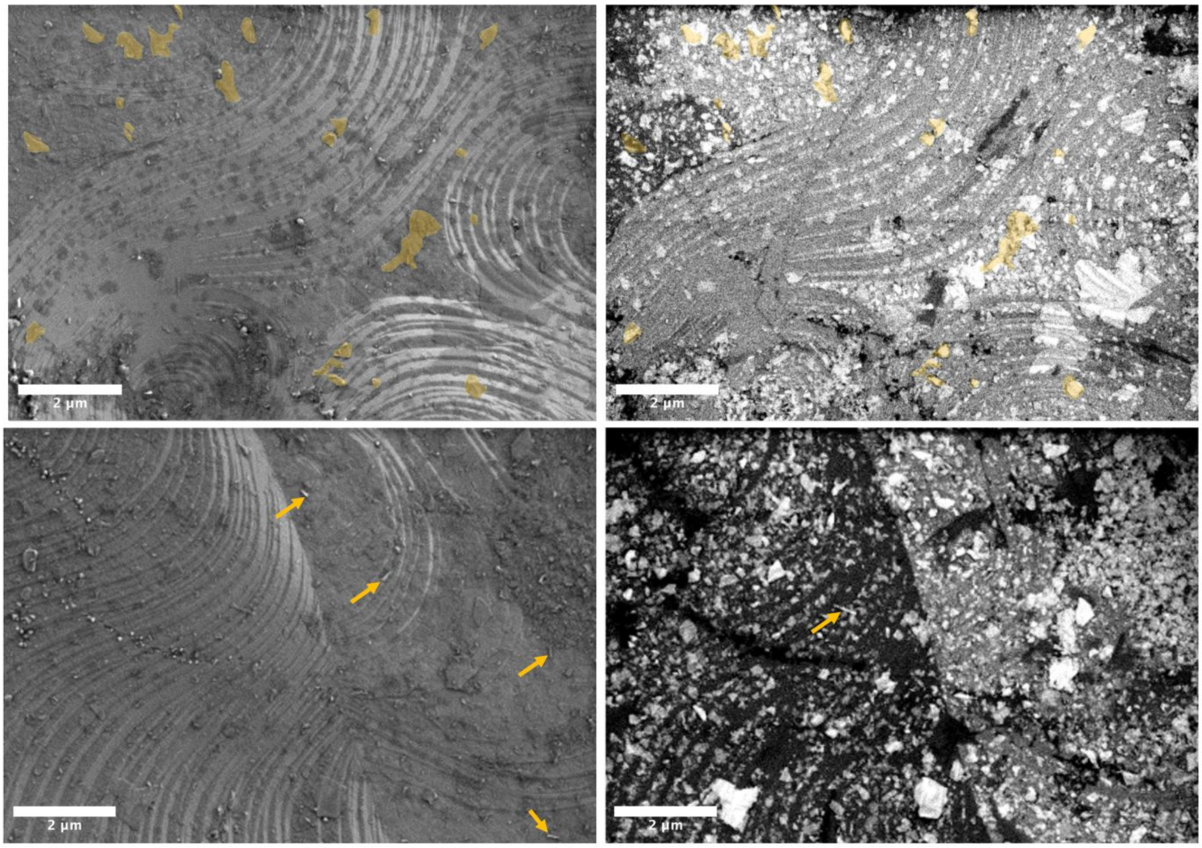

Figure S46: Scanning electron microscope micrographs of IrOOH nanosheets deposited on graphene on copper; the upper and lower row are two different areas of the sample while the right column shows images from backscattered electrons; the upper row has been used to evaluate multilayer particles (yellow areas) and their approximate thickness from upright particles (arrows in lower row) after nanosheet deposition.

Ex situ AFM

Ex situ atomic force microscopy (AFM) was used to evaluate the thickness of IrOOH sheets and the statistics of their stacking. We used monocrystalline silicon wafers to reduce the effects of the support effects. By evaluating substrate-to-sheet and sheet-to-sheet steps (see grey lines in **Figure S47**), we disentangled the offset caused by the substrate-sheet interaction from the layer height. The resulting average height of one IrOOH layer is 1.1±0.1 nm.

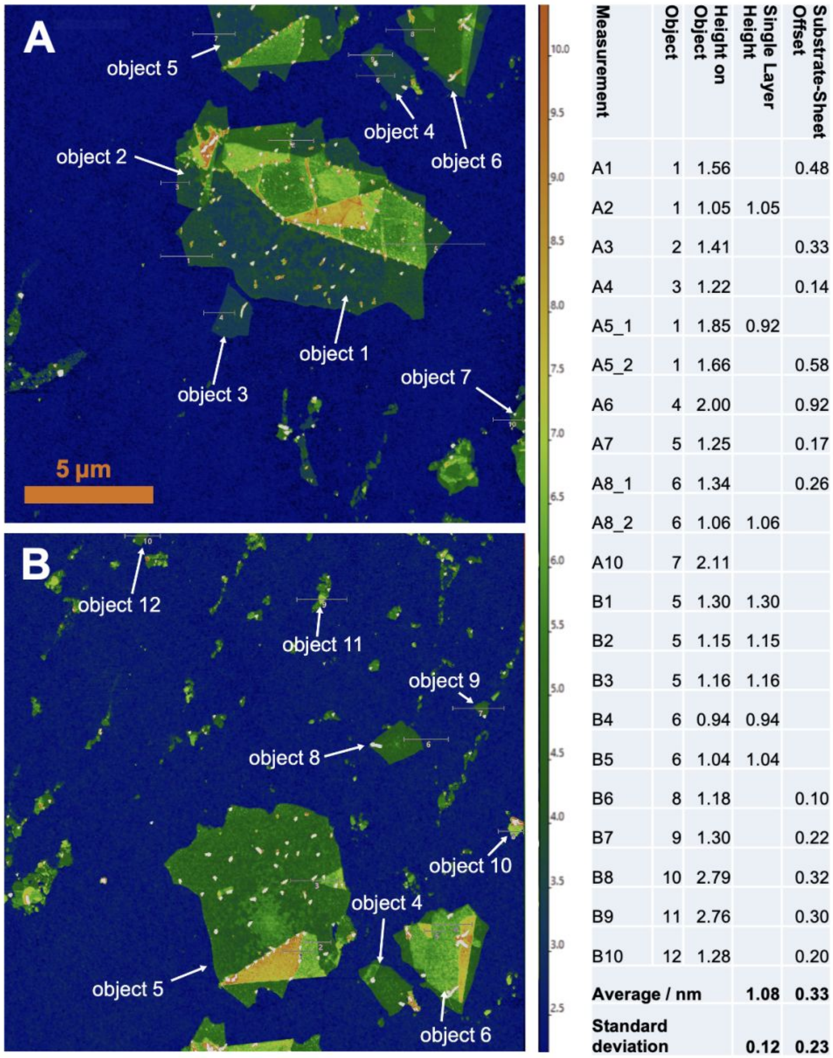

Measurement	Object	Height on Object	Single Layer Height	Substrate-Sheet Offset
A1	1	1.56		0.48
A2	1	1.05	1.05	
A3	2	1.41		0.33
A4	3	1.22		0.14
A5_1	1	1.85	0.92	
A5_2	1	1.66		0.58
A6	4	2.00		0.92
A7	5	1.25		0.17
A8_1	6	1.34		0.26
A8_2	6	1.06	1.06	
A10	7	2.11		
B1	5	1.30	1.30	
B2	5	1.15	1.15	
B3	5	1.16	1.16	
B4	6	0.94	0.94	
B5	6	1.04	1.04	
B6	8	1.18		0.10
B7	9	1.30		0.22
B8	10	2.79		0.32
B9	11	2.76		0.30
B10	12	1.28		0.20
**Average / nm**			**1.08**	**0.33**
**Standard deviation**			**0.12**	**0.23**

Figure S47: AFM micrographs of IrOOH nanosheets on silicon wafers with a color map ranging from about 2.5 to 10 nm, thin grey lines are used to mark the profiles used for the analysis; a table on the right indicates the results for the objects in images A and B.

To find the statistical coverages of monolayers, doublelayers, triplelayers and thicker stackings, the heights of the images were grouped according to layer thicknesses and the grouped areas relative to the total area covered by sheets is shown as a histogram in **Figure S48B**. It shows that almost 70% of the layers are monolayers, 20% are doublelayers, 8% are triple layers and the rest is thicker than that. From

the analysis we suspect that the folding of sheets and incomplete exfoliation contributes to this distribution.

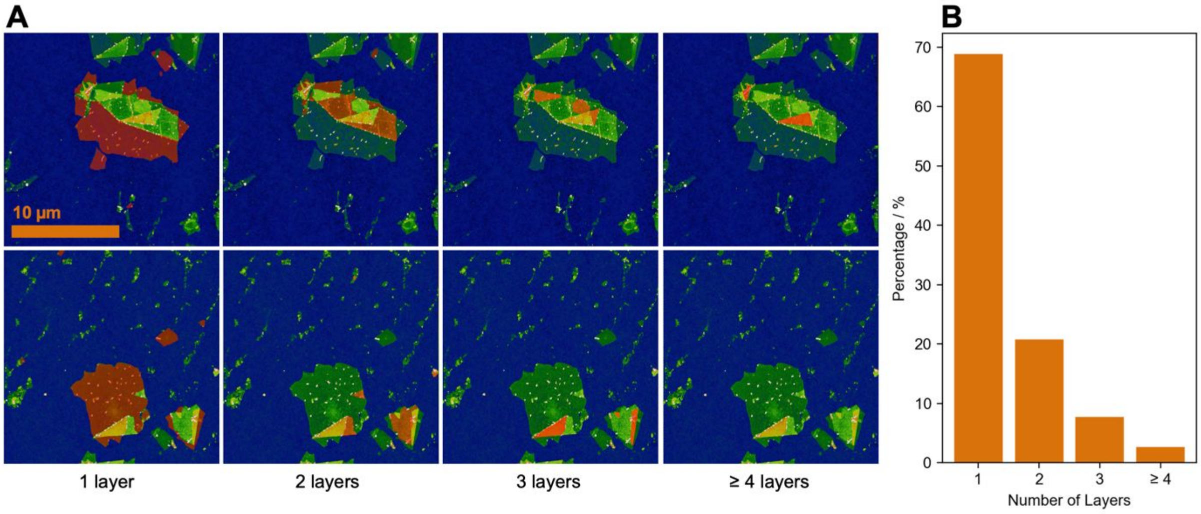

Figure S48: **A** Copies of the AFM micrographs in **Figure S47** with the difference that height responding to 1, 2, 3, and more than 4 layers are colored in orange brown; **B** Histogram of the colored areas relative to their sum.

Operando X-ray spectroscopy and electrochemistry

The in situ electrochemistry cell using polymer membranes in an electrochemical NAP-XPS module has been presented earlier.[26,27] We used these cells with a boron-doped diamond coated Nb lid contacting the sample from the top, while the backside of the membrane was in contact with a steady flow of 0.05 M H_2SO_4. A platinum wire and a Ag/AgCl reference electrode (DRIREF-2SH stored in saturated KCl solution) were immersed in the liquid electrolyte and used as counter and reference electrode, respectively. The samples were activated with 2 CVs between 0.5 V_{RHE} and 1.45 V_{RHE}. Exemplary CVs for IrOOH powder and nanosheets are provided in **Figure 6** in the main text.

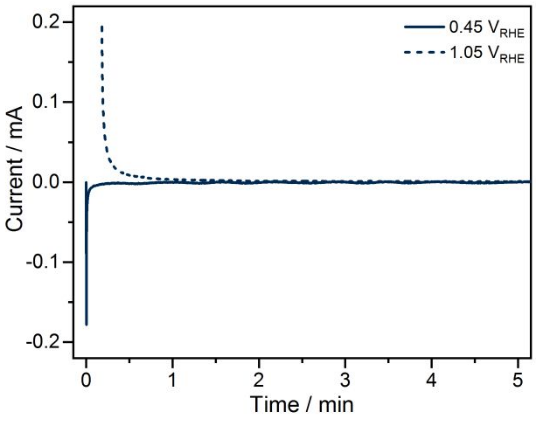

Figure S49: Chronoamperometry of (0.05 M H_2SO_4)/FAD/IrOOH-ns/SLG at 0.5 V_{RHE} and 1.1 V_{RHE}.

Operando X-ray spectroscopy was recorded at the ISISS beamline at the BESSY II synchrotron facility during low-alpha conditions. Spectra were recorded after stabilization of currents at a given potential (examples are given in **Figure S49**). Beam damage of the polymer membrane was minimized by moving to a new measurement position after each potential step. It was assured that the samples were homogeneous enough in the measured region to provide consistent data sets. Measurements recorded after more than 5 min on the same spot were not used for detailed analysis. The pressure in the main chamber was regulated by additional water dosing and a PID-controlled pressure valve by Pfeiffer Vacuum GmbH (Asslar, Germany). The target water pressure of 0.25 mbar was sustained throughout operando spectroscopy, except for the samples containing Ir NPs, for which the pressure ranged between 0.05 and 0.15 mbar.

Table S8: Ir 4f fit parameters of IrOOH-ns in 0.1 M H_2SO_4 at 0.5 V_{RHE} and 1.1 V_{RHE}; Line shapes are given in reference to the CasaXPS software,[28] an equal-sign indicates a condition for the fit.

$Ir^{III}OOH$ @ 0.5 V_{RHE}	A Ir 4f 7/2	B Ir 4f 5/2		
Line shape	SGL(35)	SGL(35)		
Center / eV	62.0	A + 3.0		
FWHM / eV	1.26	= A		
Area	58%	42%		
**$Ir^{IV}OO$ @ 1.1 V_{RHE}**	**A' Ir 4f 7/2**	**B' Ir 4f 5/2**	**C & D Sat 1**	**E & F Sat 2**
Line shape	DS(0.05, 200) SGL(35)	DS(0.05, 200) SGL(35)	SGL(35)	SGL(35)
Center / eV	61.7	A' + 3.0	D = C + 3.0 (A' or B') + 1.0	E = F + 3.0 (A' or B') + 2.2
FWHM / eV	1.26	= A'	C = D 1.62	E = F 1.2
Area	38%	A'/B' = 4/3 28%	C/D = 4/3 15%+11%	E/F = 4/3 4%+3%

For comparison to **Figure 7** in the main text, Ir 4f and O K-edge absorption of AA-IrOx (**Figure S50**), Ir nanoparticles (NPs) (**Figure S51**), and bulk IrOOH (**Figure S52**) are provided in the state of Ir^{III} and Ir ^{IV}. Fitting restrictions are provided in **Table S8**; with the exception of Ir NPs, for which metallic iridium was fitted in addition, with the Ir 4f 7/2 contribution appearing at 60.9 eV, a doublet splitting of 3.0 eV, and a Doniach-Ŝunjić line shape. The trends in the O K-edge absorption are the same for all samples. One main feature is observed for the e_g-contribution between 532 eV and 533 eV, which appears in all spectra, while a t_{2g}-feature appears at about 529 eV in the state of Ir^{IV}, or at ~1.2 V_{RHE}. An exception are contributions which are difficult to reduces, such as the rutile structure and possibly the hollandite structure found in AA-IrOx (see above), which absorb at 530 eV. The Ir 4f doublets at low potentials

can be fitted with two summed Gaussian-Lorentzian contributions. The Ir 4f 7/2 contribution is centered at a BE of about 62 eV. The full width half maximum (FWHM) of the contribution varies between 1.2 eV and 1.8 eV. The Ir 4f fit of AA-IrOx at 0.45 V_{RHE} (**Figure S50B**) lacks some high-BE broadening, which is again caused by irreducible Ir^{IV} oxide contributions. In the oxidized form, all three samples can be fitted with three doublets, one doublet with the Doniach-Ŝunjić line shape and two satellite features with a summed Gaussian-Lorentzian as their line shape. The distance to the main peak varies from 0.6-1.0 eV for the first satellite and the second satellite has a distance of 1.9-2.1 eV from the main line. The FWHM varies between 1.0 eV and 1.8 eV. This means that the more complex bulk with partly amorphous structures presented here are not precisely described by the fit, but the electronic structure appears similar.

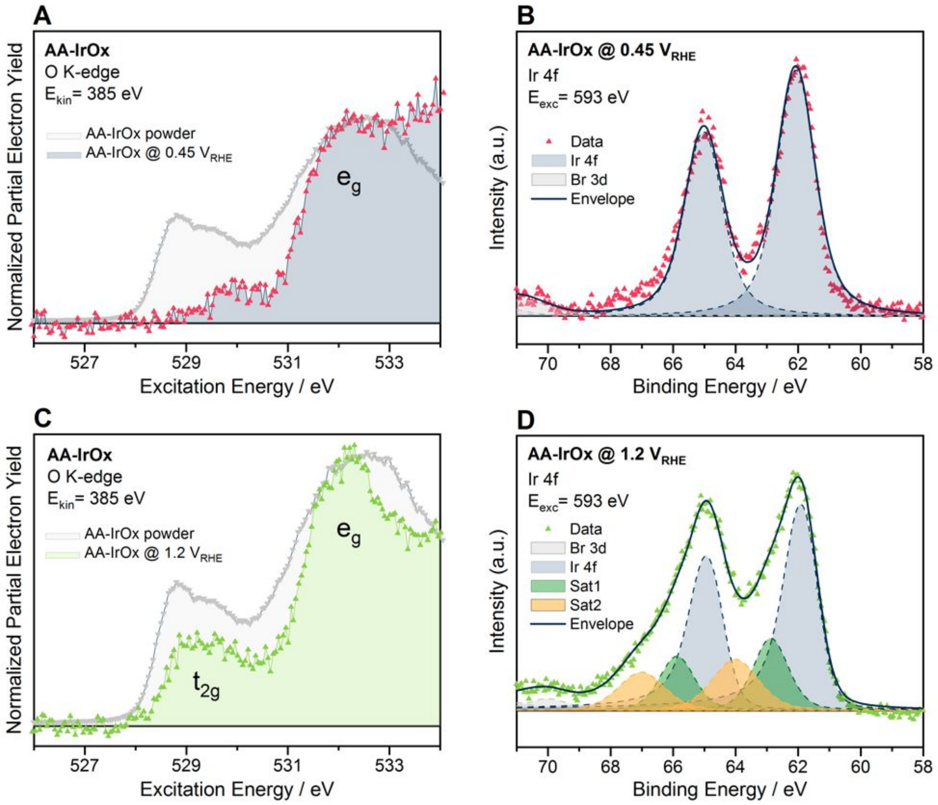

Figure S50: a) c) O K-edge and b) d) Ir 4f XP spectra of (0.05 M H_2SO_4)/FAD/AA-IrOx/BLG at a) b) 0.45 V_{RHE} and c) d) 1.2 V_{RHE}; a Shirley-type background was subtracted from the Ir 4f XP spectrum.

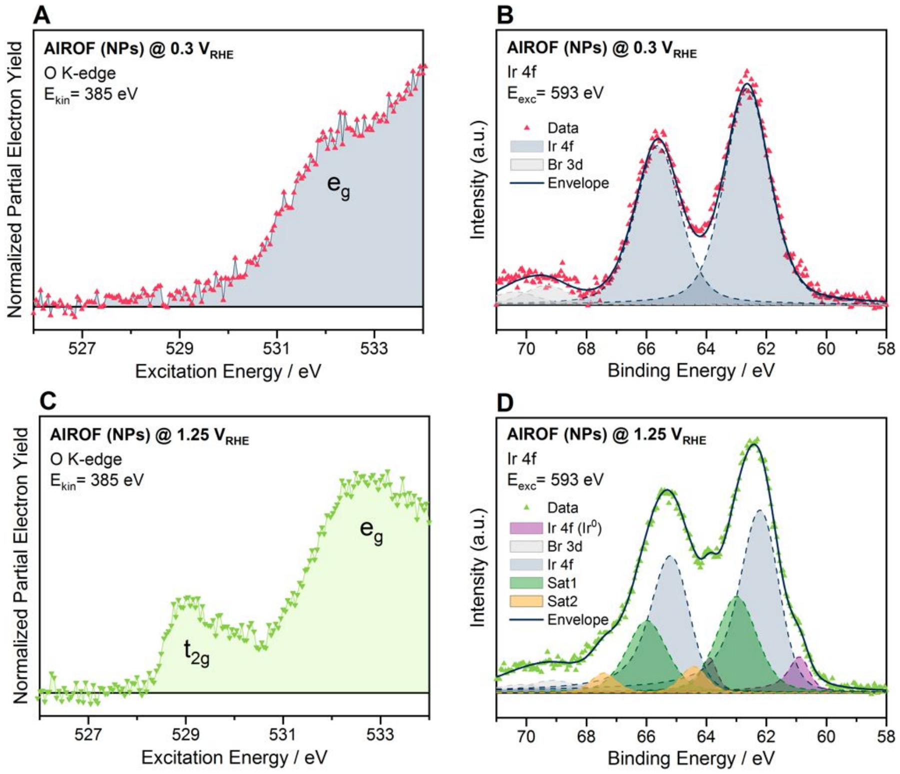

Figure S51: a) c) O K-edge and b) d) Ir 4f XP spectra of (0.1 M H_2SO_4)/FAD/Ir NPs/SLG at a) b) 0.3 V_{RHE} and c) d) 1.25 V_{RHE}; a Shirley-type background was subtracted from the Ir 4f XP spectrum.

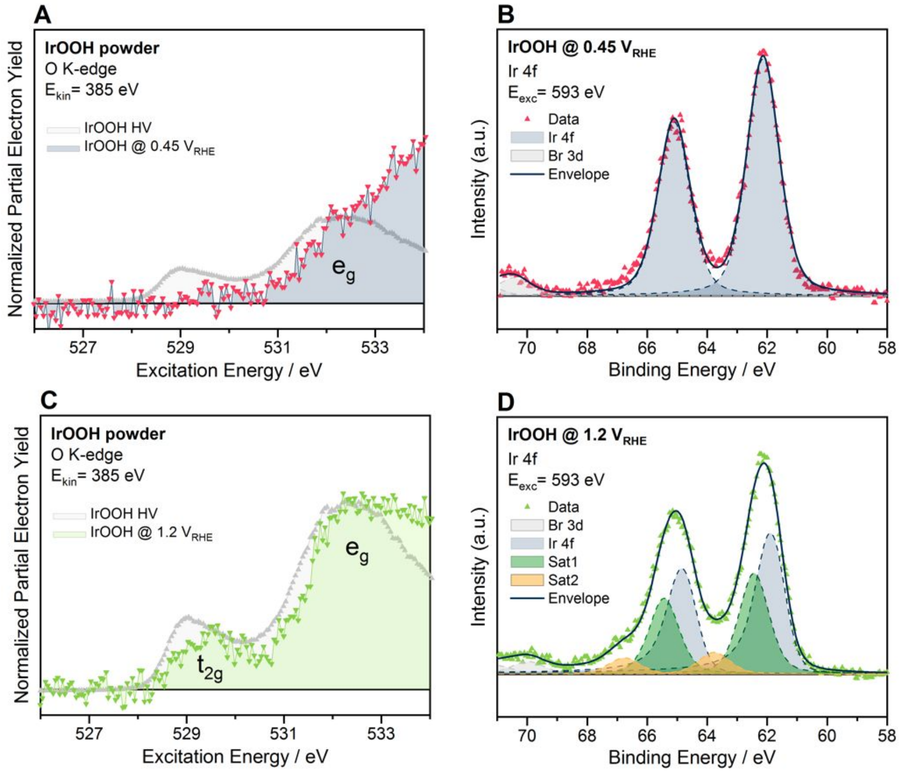

Figure S52: a) c) O K-edge and b) d) Ir 4f XP spectra of (0.05 M H_2SO_4)/FAD/IrOOH/BLG at a) b) 0.45 V_{RHE} and c) d) 1.2 V_{RHE}; a Shirley-type background was subtracted from the Ir 4f XP spectrum.

In **Figure S53** the Ir 4f XP spectra and the O K-edge absorption as well as their development towards OER relevant potentials are compared for IrOOH-ns, AA-IrOx, Ir NPs, and IrOOH. The Ir 4f spectra in all samples develop a more asymmetric line shape with higher potentials. This broadening towards higher binding energies is associated with higher oxidation states of iridium.[29] It is most pronounced in the oxidation from low potentials to ~1.2 V_{RHE}, but further asymmetric broadening is observed in potentials relevant to OER. However, a minor additional broadening is observed in AA-IrOx and Ir NPs, while IrOOH-ns and IrOOH powder continue to change beyond the potential of 1.4 V_{RHE}. However, the broadening could also originate from charging behavior, and this is why it was not quantified. The decreased signal to noise ratio of Ir 4f spectra towards higher potentials can either be explained by enclosure of catalyst particles by the polymers in the FAD membrane, or by charging.

The corresponding trend to the Ir 4f asymmetry in the O K-edge absorption is a shift of the white line towards lower excitation energies. The largest change occurs, again, in the oxidation up to ~1.2 V_{RHE}, in which an absorption feature between 529 eV and 530 eV appears. A second absorption feature slightly below 529 eV is present at 1.2 V_{RHE} in AA-IrOx and at ~1.4V_{RHE} in Ir NPs and IrOOH. At potentials relevant to the OER, further broadening to lower excitation energies is consistently observed in all samples of the set. It can be summarized that the Ir 4f XP and the O K-edge absorption spectra follow the same trends but vary in the details of energy position and intensity distribution, pointing towards a different electronic and chemical structure, i.e. a variation in the distribution of oxygen species (μ_1-O, μ_2-O, or μ_3-O). The O K-edge measurements in **Figure S53** should be influenced little by charging of the sample, nor by the enclosure of particles, due to their normalization to the white line intensity originating from largely Ir–O species.

A similar issue with normalization of the white line appears when attempting to quantify the interlayer proton concentration. **Figure S54** compares in-situ and ex-situ O K-edge absorption spectra of the IrOOH powder normalized at 532 eV. The similarity of the powder, where every second interlayer hydrogen is missing, and the in-situ measurement at 1.3 V is striking. Since we expect iridium to be at or beyond a formal oxidation state of +4 at 1.3 V, this could indicate that the surface layer of the powder is already oxidized to +4 in the powder and that most of the interlayer protons are trapped further inside the particles. Another valid explanation is that the normalization to 532 eV leads to an underestimation of the white line intensity in the in-situ measurement, because carbonaceous species contribute at 532 eV, too. So we cannot derive any interlayer proton density from our absorption measurements.

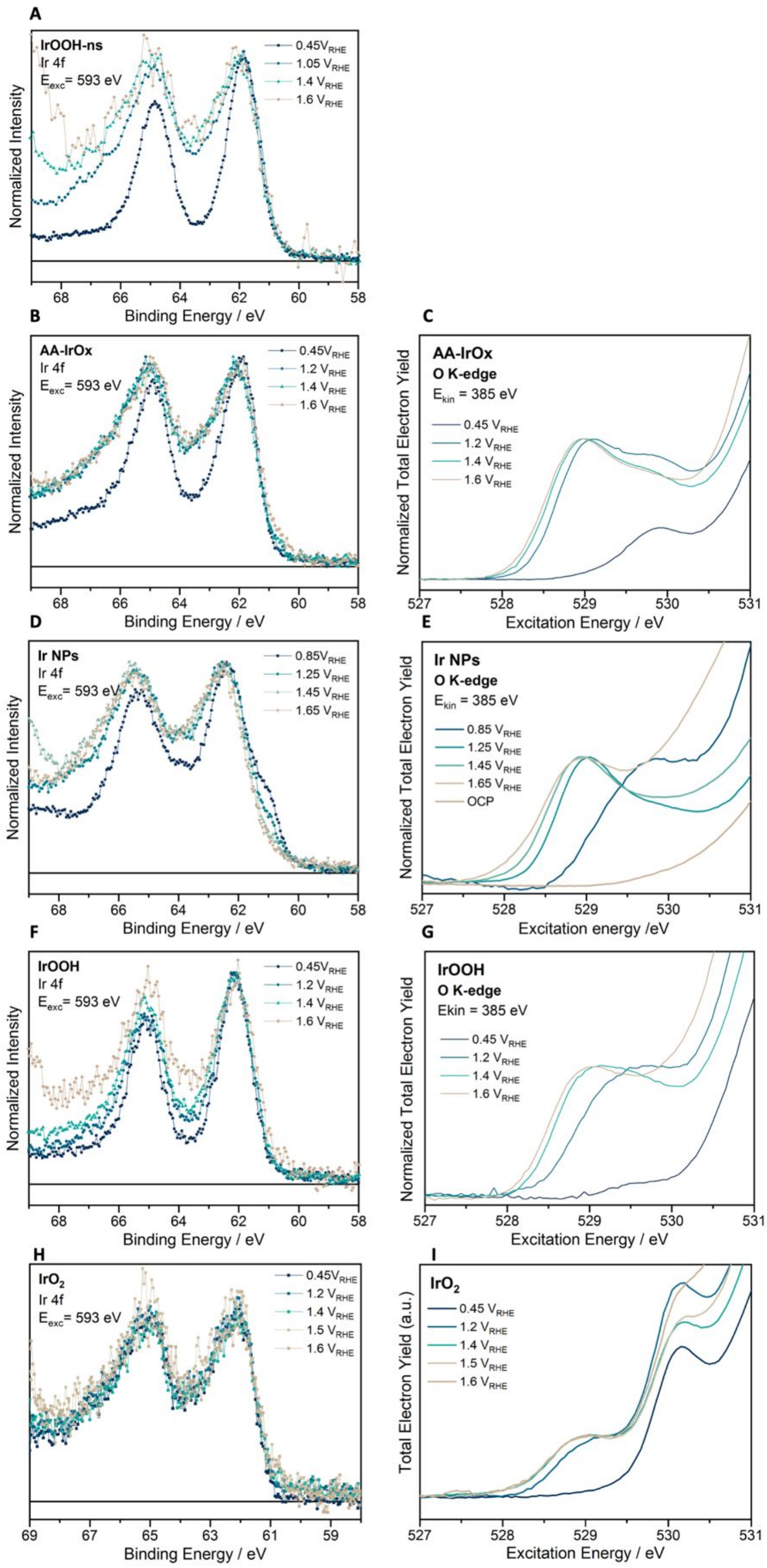

Figure S53: In situ and operando Ir 4f XP spectra (left) and O K-edge absorption spectra (right) of a) (0.05 M H_2SO_4)/FAD/IrOOH-ns/SLG, and b)-i) (0.05 M H_2SO_4)/FAD/*Ir catalyst*/BLG; XP spectra were normalized to the Ir 4f 7/2 peak intensity and absorption spectra were normalized to the peak intensity of the pre-edge; in the case of IrNPs the electrolyte was 0.1 M H_2SO_4.

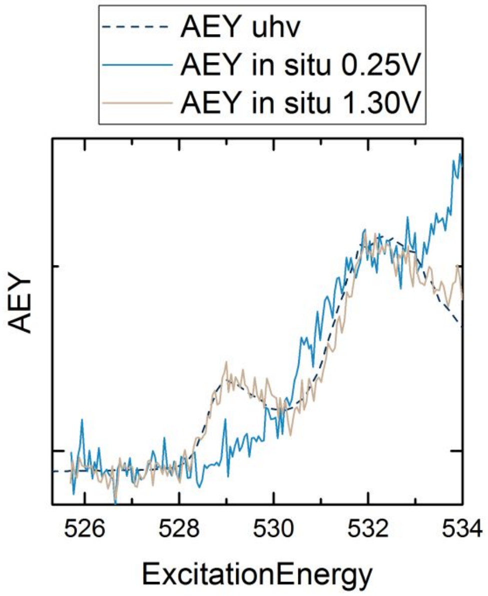

Figure S54: O K-edge absorption spectra of the IrOOH powder measured in Auger electron yield (AEY); solid lines represent in situ spectra and dashed line is a UHV experiment of a powder pellet; all spectra were normalized to the mean of 5 points centered at 532 eV.

Bibliography

(1) Weber, Daniel; Schoop, Leslie M.; Wurmbrand, Daniel; Nuss, Jürgen; Seibel, Elizabeth M.; Tafti, Fazel Fallah; Ji, Huiwen; Cava, Robert J.; Dinnebier, Robert E.; Lotsch, Bettina V. Trivalent Iridium Oxides: Layered Triangular Lattice Iridate K0.75 Na0.25 IrO2 and Oxyhydroxide IrOOH. *Chem. Mater.* **2017**, *29*, 8338–8345. https://doi.org/10.1021/acs.chemmater.7b02683.

(2) Yeh, J. J.; Lindau, I. Atomic Subshell Photoionization Cross Sections and Asymmetry Parameters: 1 < Z < 103. *At Data Nucl Data Tables* **1985**, *32* (1), 1–155. https://doi.org/10.1016/0092-640X(85)90016-6.

(3) Tanuma, Shigeo; Powell, Cedric J.; Penn, David R. Calculations of Electron Inelastic Mean Free Paths. V. Data for 14 Organic Compounds over the 50-2000 EV Range. *Surface and Interface Analysis* **1994**, *21* (3), 165–176. https://doi.org/10.1002/sia.740210302.

(4) Weber, Daniel; Schoop, Leslie M.; Wurmbrand, Daniel; Laha, Sourav; Podjaski, Filip; Duppel, Viola; Müller, Kathrin; Starke, Ulrich; Lotsch, Bettina V. IrOOH Nanosheets as Acid Stable Electrocatalysts for the Oxygen Evolution Reaction. *Journal of tMaterials Chemistry A* **2018**, *6* (43), 21558–21566. https://doi.org/10.1039/C8TA07950A.

(5) Bette, Sebastian; Hinrichsen, Bernd; Pfister, Daniela; Dinnebier, Robert, E. A Routine for the Determination of the Microstructure of Stacking-Faulted Nickel Cobalt Aluminium Hydroxide Precursors for Lithium Nickel Cobalt Aluminium Oxide Battery Materials Microstructure Determination for NCA Precursors. *Journal of Applied Crystallography* **2020**, *53*, 76–87. https://doi.org/10.1107/S1600576719016212.

(6) Stephens, Peter W. Phenomenological Model of Anisotropic Peak Broadening in Powder Diffraction. *Journal of Applied Crystallography* **1999**, *32* (2), 281–289. https://doi.org/10.1107/S0021889898006001.

(7) Hölderle, T.; Monchak, M.; Baran, V.; Dolotko, O.; Bette, S.; Mikhailova, D.; Voss, A.; Avdeev, M.; Ehrenberg, H.; Müller-Buschbaum, P.; Senyshyn, A. The Structural Behavior of Electrochemically Delithiated LixNi0.8Co0.15Al0.05O2 (X<1) Battery Cathodes. *Journal of Power Sources* **2023**, *564* (March), 232799. https://doi.org/10.1016/j.jpowsour.2023.232799.

(8) Juhás, P.; Davis, T.; Farrow, C. L.; Billinge, S. J. L. PDFgetX3 : A Rapid and Highly Automatable Program for Processing Powder Diffraction Data into Total Scattering Pair Distribution Functions. *Journal of Applied Crystallography* **2013**, *46* (2), 560–566. https://doi.org/10.1107/S0021889813005190.

(9) Yang, Xiaohao; Juhas, Pavol; Farrow, Christopher L.; Billinge, Simon J. L. XPDFsuite: An End-to-End Software Solution for High Throughput Pair Distribution Function Transformation, Visualization and Analysis. *Journal of Applied Crystallography* **2014**.

(10) Egami, Takeshi; Billinge, Simon. *Underneath the Bragg Peaks, Volume 16*, 2nd Editio.; Elsevier Ltd: Oxford, Amsterdam, San Diego, 2012.

(11) Lorch, E. Neutron Diffraction by Germania, Silica and Radiation-Damaged Silica Glasses. *Journal of Physics C: Solid State Physics* **1969**, *2* (2), 305. https://doi.org/10.1088/0022-3719/2/2/305.

(12) Soper, Alan K.; Barney, Emma R. On the Use of Modification Functions When Fourier Transforming Total Scattering Data. *Journal of Applied Crystallography* **2012**, *45* (6), 1314–1317. https://doi.org/10.1107/S002188981203960X.

(13) Farrow, C. L.; Juhas, P.; Liu, J. W.; Bryndin, D.; Božin, E. S.; Bloch, J.; Proffen, Th; Billinge, S. J. L. PDFfit2 and PDFgui: Computer Programs for Studying Nanostructure in Crystals. *Journal*

of Physics: Condensed Matter **2007**, *19* (33), 335219. https://doi.org/10.1088/0953-8984/19/33/335219.

(14) Coelho, Alan A. TOPAS and TOPAS-Academic : An Optimization Program Integrating Computer Algebra and Crystallographic Objects Written in C++. *Journal of Applied Crystallography* **2018**, *51* (1), 210–218. https://doi.org/10.1107/S1600576718000183.

(15) Willinger, Elena; Massué, Cyriac; Schlögl, Robert; Willinger, Marc Georg. Identifying Key Structural Features of IrOx Water Splitting Catalysts. *Journal of the American Chemical Society* **2017**, *139* (34), 12093–12101. https://doi.org/10.1021/jacs.7b07079.

(16) Tauc, J.; Grigorovici, R.; Vancu, A. Optical Properties and Electronic Structure of Amorphous Germanium. *Physica Status Solidi (B)* **1966**, *15* (2), 627–637. https://doi.org/10.1002/pssb.19660150224.

(17) Nilsson, A.; Nordlund, D.; Waluyo, I.; Huang, N.; Ogasawara, H.; Kaya, S.; Bergmann, U.; Näslund, L. Å.; Öström, H.; Wernet, Ph; Andersson, K. J.; Schiros, T.; Pettersson, L. G. M. X-Ray Absorption Spectroscopy and X-Ray Raman Scattering of Water and Ice; an Experimental View. *J. Electron Spectrosc. Relat. Phenom.* **2010**, *177*, 99–129. https://doi.org/10.1016/j.elspec.2010.02.005.

(18) Hitchcock, A. P.; Brion, C. E. K-Shell Excitation Spectra of CO, N2 and O2. *Journal of Electron Spectroscopy and Related Phenomena* **1980**, *18* (1), 1–21. https://doi.org/10.1016/0368-2048(80)80001-6.

(19) McDaniel, C. L.; Schneider, S. J. Phase Relations in the Systems TiO2-IrO2 and SnO2-IrO2 in Air. *Journal of Research of the National Bureau of Standards Section A: Physics and Chemistry* **1967**, *71A* (2), 119. https://doi.org/10.6028/jres.071a.016.

(20) Nørskov, J. K.; Rossmeisl, J.; Logadottir, A.; Lindqvist, L.; Kitchin, J. R.; Bligaard, T.; Jónsson, H. Origin of the Overpotential for Oxygen Reduction at a Fuel-Cell Cathode. *The Journal of Physical Chemistry B* **2004**, *108* (46), 17886–17892. https://doi.org/10.1021/jp047349j.

(21) Henkelman, Graeme; Uberuaga, Blas P.; Jónsson, Hannes. A Climbing Image Nudged Elastic Band Method for Finding Saddle Points and Minimum Energy Paths. *The Journal of Chemical Physics* **2000**, *113* (22), 9901–9904. https://doi.org/10.1063/1.1329672.

(22) Ping, Yuan; Nielsen, Robert J.; Goddard, William A. The Reaction Mechanism with Free Energy Barriers at Constant Potentials for the Oxygen Evolution Reaction at the IrO 2 (110) Surface.

Journal of the American Chemical Society **2017**, *139* (1), 149–155. https://doi.org/10.1021/jacs.6b07557.

(23) Pfeifer, Verena; Jones, Travis E.; Wrabetz, Sabine; Massué, Cyriac; Velasco Vélez, Juan J.; Arrigo, Rosa; Scherzer, Michael; Piccinin, Simone; Hävecker, Michael; Knop-Gericke, Axel; Schlögl, Robert. Reactive Oxygen Species in Iridium-Based OER Catalysts. *Chem. Sci.* **2016**, *7*, 6791–6795. https://doi.org/10.1039/c6sc01860b.

(24) Klinger, Miloslav; Jäger, Aleš. Crystallographic Tool Box (CrysTBox): Automated Tools for Transmission Electron Microscopists and Crystallographers. *Journal of Applied Crystallography* **2015**, *48* (6), 2012–2018. https://doi.org/10.1107/S1600576715017252.

(25) Trucano, Peter; Chen, Ruey. Structure of Graphite by Neutron Diffraction. *Nature* **1975**, *258* (5531), 136–137. https://doi.org/10.1038/258136a0.

(26) Knop-Gericke, Axel; Pfeifer, Verena; Velasco-Velez, Juan-Jesus; Jones, Travis; Arrigo, Rosa; Hävecker, Michael; Schlögl, R. In Situ X-Ray Photoelectron Spectroscopy of Electrochemically Active Solid-Gas and Solid-Liquid Interfaces. *J. Electron Spectrosc. Relat. Phenom.* **2017**, *221*, 10–17. https://doi.org/10.1016/j.elspec.2017.03.010.

(27) Carbonio, Emilia A.; Velasco-Velez, Juan-Jesus; Schlögl, Robert; Knop-Gericke, Axel. Perspective—Outlook on Operando Photoelectron and Absorption Spectroscopy to Probe Catalysts at the Solid-Liquid Electrochemical Interface. *Journal of The Electrochemical Society* **2020**, *167* (5), 054509. https://doi.org/10.1149/1945-7111/ab68d2.

(28) Fairley, Neal; Carrick, Alan. CasaXPS. 2017.

(29) Velasco-Vélez, Juan Jesús; Carbonio, Emilia A.; Chuang, Cheng Hao; Hsu, Cheng Jhih; Lee, Jyh Fu; Arrigo, Rosa; Hävecker, Michael; Wang, Ruizhi; Plodinec, Milivoj; Wang, Feng Ryan; Centeno, Alba; Zurutuza, Amaia; Falling, Lorenz J.; Mom, Rik Valentijn; Hofmann, Stephan; Schlögl, Robert; Knop-Gericke, Axel; Jones, Travis E. Surface Electron-Hole Rich Species Active in the Electrocatalytic Water Oxidation. *Journal of the American Chemical Society* **2021**, *143* (32), 12524–12534. https://doi.org/10.1021/jacs.1c01655.
